# Tracking polar solvation dynamics of a photoexcited organic chromophore with ultrafast X-ray scattering

**DOI:** 10.1038/s41467-026-71635-1

**Published:** 2026-04-13

**Authors:** Kerstin M. Mitterer, Elli Selenius, Morten L. Haubro, Magnus A. H. Christiansen, Verena Markmann, Bianca L. Hansen, Mikkel Krell-Jørgensen, Joseph G. F. Hoock, Victor Lorentzen, Emma V. Beale, Philip J. M. Johnson, David J. Gosztola, Claudio Cirelli, Camila Bacellar, Asmus O. Dohn, Luca Laraia, Klaus B. Møller, Kristoffer Haldrup, Gianluca Levi, Martin M. Nielsen

**Affiliations:** 1 Department of Physics, Technical University of Denmark, Kongens Lyngby, Denmark; 2 Science Institute and Faculty of Physical Sciences, University of Iceland, Reykjavík, Iceland; 3 Department of Chemistry, Technical University of Denmark, Kongens Lyngby, Denmark; 4 Paul Scherrer Institut, Villigen PSI, Switzerland; 5 Center for Nanoscale Materials, Argonne National Laboratory, Lemont, IL USA; 6 Department of Chemical and Pharmaceutical Sciences, University of Trieste, Trieste, Italy

**Keywords:** Excited states, Electron transfer, Reaction kinetics and dynamics, Electronic structure of atoms and molecules, Atomic and molecular interactions with photons

## Abstract

Solvation plays a key role in many photochemical and biological processes. Yet, direct atomic-scale observation of solvent reorganization around photoexcited molecules containing only light atoms has so far remained elusive. Here, we use time-resolved X-ray scattering at an X-ray free-electron laser to track with a  ~ 120 fs time resolution the photoinduced ultrafast rearrangement of polar acetonitrile solvent molecules around an organic hemithioindigo chromophore. The experiments reveal that the solvation shell reorganizes with a  ~ 0.3 ps time constant driven by photoinduced charge transfer in the chromophore, and then reequilibrates over  ~ 3 ps as the excited state population returns to the ground state. These results demonstrate the viability of using ultrashort X-ray pulses to directly visualize the motion of light atoms in solution, paving the way for atomic-scale understanding and control of photoinduced processes in biological and synthetic photoactive organic molecules.

## Abstract (web summary)

Solvation dynamics shape many light-driven reactions but are challenging to observe. Time-resolved Xray scattering reveals the atomic-scale ultrafast reorganization of a polar solvent driven by charge transfer in a photoexcited organic molecule.

## Introduction

Typically, photochemical and photophysical processes occur in a condensed-phase environment. In a liquid solution, the solvent molecules are not just spectators but play an active role, sometimes by directly participating in photochemical transformations, such as in photoinduced ligand substitution reactions of solvated metal complexes^1,2^. More generally, changes in the electronic and atomic distribution of a solute upon photoexcitation trigger the rearrangement of surrounding solvent molecules to minimize the overall free energy, thereby affecting the rates and outcomes of the excited state relaxation^3–6^. These nonequilibrium solvation dynamics are especially critical when they involve the response of a polar solvent to a photoinduced charge transfer. In this case, the solvent molecules adapt to the change in the local electric field of the excited solute by reorienting their dipole, a process known as polar solvation dynamics. This response in turn modulates the charge distribution, atomic structure and energetics of the photoexcited chromophore^5,7–10^. Understanding polar solvation dynamics at the atomic level is essential, as photoinduced charge transfer is a key step in fundamental natural processes such as photosynthesis^11^ and applications in, e.g., solar energy conversion^12^ and optoelectronics^13^.

Our current understanding of polar solvation dynamics stems largely from time-resolved optical spectroscopy experiments^5,8,14^ and theoretical studies, particularly molecular dynamics (MD) simulations^15–24^, conducted over the past few decades. The free energy minimization induced by the solvation-shell response gives rise to shifts of emission and absorption spectra. A common approach is to track the dynamic Stokes shift, i.e., the time-dependent redshift of fluorescence^25–27^. More recently, time-resolved terahertz spectroscopy and nonlinear spectroscopy techniques, particularly two-dimensional electronic and electronic-vibrational spectroscopies, have also been employed to investigate solvation dynamics triggered by photoinduced processes^28–30^. These approaches are important to gain insights into the excited state solute-solvent interactions driving solvation dynamics, but they do not directly provide an atomic-scale picture of the solvent rearrangement.

Ultrashort, highly brilliant X-ray pulses generated at X-ray free-electron lasers (XFELs) make it possible to track photoinduced processes at the atomic length scale. In particular, time-resolved X-ray solution scattering (TR-XSS) at XFELs^31,32^ can directly probe changes in atomic arrangements (time-resolved X-ray liquidography^32^), both in the solute^33–40^ and surrounding solvent, including rearrangements of the solvation shell^17,21,41–43^. TR-XSS has been used to elucidate the solvation-shell rearrangement of photoexcited diatomic halogen molecules^44^ and metal complexes^21,43^, the coherent solvent response to charge transfer in a bimetallic complex^17^, the solvent mediated photodissociation and associated atomic charge redistribution of triiodide^39,42^, the solvation dynamics following photoabstraction of an electron from halogen ions^16,41^. All these TR-XSS studies leveraged the presence of electron-rich atoms in the solute, which amplifies both the solute and the solvation-shell response signals relative to the large scattering contribution of the bulk solvent, facilitating signal acquisition and interpretation^45^. In refs. ^17,39^, this made it possible to infer the charge redistribution within the solute through the solvent response.

The direct observation of atomic motions involving organic molecules, containing only light, weakly scattering atoms, has so far been limited to the gas phase^32,46^. A recent study by Ihee and coworkers^47^ used TR-XSS to probe the kinetics and transient intermediates of a photoexcited solvated *p*-hydroxyphenacyl organic molecule indirectly, by monitoring the solvent heating signal due to energy transfer from the solute. However, to our knowledge, no experimental study to date has directly captured atomic-scale solvation dynamics around an organic chromophore. This represents a significant gap, given the importance of organic photoactive molecules for energy conversion, optoelectronics, photopharmacology, and biological systems^14^. Direct structural insights into the solvent response are essential for understanding how the local environment governs photorelaxation pathways. Such knowledge could ultimately enable control over the lifetime of charge-separated states and help reduce energy loss due to thermal dissipation, thereby improving efficiency and stability in photosensitization and photoconversion.

**Fig. 1:**
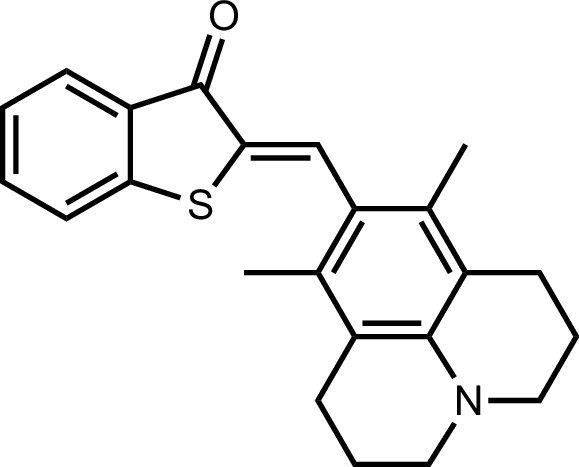
Structure of HTI-J. The hemithioindigo compound investigated in the present work, consisting of a thioindigo and a julolidine-based electron donating group.

Here, the ultrafast, atomic-scale solvation dynamics of the polar solvent acetonitrile triggered by charge transfer in a photoexcited hemithioindigo (HTI) organic chromophore are directly captured using TR-XSS measurements at the SwissFEL XFEL facility. HTIs^4,48–51^ consist of thioindigo and stilbene fragments connected by a double and a single carbon-carbon bond. Previously, it has been shown that the photorelaxation of HTIs with strong electron-donating dialkylamino substituents is dramatically influenced by the solvent: In apolar solvents, photoexcitation predominantly induces isomerization from a *Z* to an *E* isomer (see Suppl. Fig. 27) via double bond twisting, whereas in polar solvents, the formation of a twisted intramolecular charge transfer state has been proposed to dominate^4,49^. Evidence for this charge transfer state in polar environments includes a reduced *Z*/*E* photoisomerization yield ( < 1%), dual fluorescence, and red-shifted features in transient optical absorption, emission, and time-resolved vibrational spectra^4,49^. A ground state geometry partially twisted about the connecting single bond (see Suppl. Note 9) has been proposed as a prerequisite for formation of the intramolecular charge transfer state^50^, wherein the thioindigo and stilbene units may further twist, potentially adopting a near-perpendicular conformation. In the present work, measurements are performed on an HTI where the stilbene moiety is replaced by a strong electron donating julolidine group (hereafter referred to as HTI-J, see Fig. 1). A large increase in the dipole moment of HTI-J upon electronic excitation, from 5.3 to 27.3 D at the Franck-Condon geometry, is predicted by density functional theory (DFT) calculations (see Suppl. Note 9). Therefore, photoexcitation in acetonitrile is expected to induce a large reorganization of the solvent molecules around the solute.

**Fig. 2:**
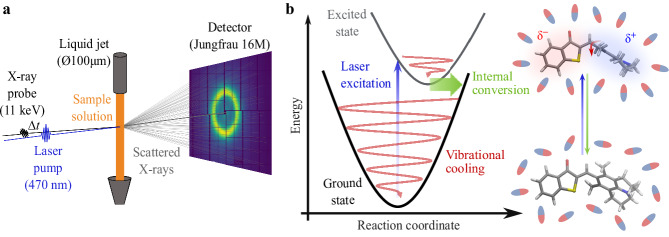
TR-XSS experimental setup and potential energy diagram for HTI-J. **a** Schematic of the setup for the time-resolved X-ray solution scattering experiments. The optical laser pump pulse excites the sample delivered by a liquid jet, which is then probed by an X-ray probe pulse after a variable time delay, Δ*t*. The forward scattering is recorded on an area detector. **b** Potential energy diagram and depiction of the the solvated HTI-J molecule, illustrating the photoinduced dynamics revealed by the present experiments and density functional theory (DFT) calculations (atom colors: C gray, H white, N blue, O red, S yellow, N and methyl ends of the acetonitrile solvent molecules are red and blue, respectively). Laser excitation induces intramolecular charge transfer followed by solvent reorganization and relaxation in the excited state through vibrational cooling and structural rearrangement. DFT calculations predict a twisting of HTI-J, while the present TR-XSS experiments reveal the solvation-shell rearrangement in response to intramolecular charge transfer. The HTI-J decays to the ground state via internal conversion, accompanied by thermal heating of the chromophore and reequilibration of the solvation shell, before ground state vibrational cooling.

Figure 2 illustrates the experimental setup for the TR-XSS measurements, along with the dynamical processes following photoexcitation of the HTI-J molecule, as revealed by the TR-XSS and supporting transient absorption spectroscopy (TAS) experiments and DFT calculations. By analyzing the time-resolved X-ray scattering signal with the support of MD simulations, the solvation-shell response is disentangled from the dominant scattering contribution of bulk solvent heating and other structural changes arising from vibrational relaxation. It is found that reorganization of the acetonitrile molecules around the solute is driven by intramolecular charge transfer and occurs with a timescale of  ~ 0.3 ps. The solvation shell then reequilibrates as the excited state population undergoes internal conversion returning to the ground electronic state on a  ~ 2.6 ps timescale.

This work demonstrates the feasibility of visualizing motions of light atoms in solution via ultrafast hard X-rays, highlighting their potential as a powerful tool for probing solvation phenomena in organic photochemistry at the atomic scale. By directly tracking the polar solvation dynamics following photoexcitation of HTI-J, it is shown that the solvent response is dominated by intramolecular charge transfer, which drives a pronounced reorganization of the surrounding solvation shell, rather than by a structural change of the chromophore. Thus, our results provide structural evidence for the formation of a charge transfer excited state in electron donor substituted HTIs in polar solvent, and yield insights into the microscopic mechanism underlying polar solvation dynamics in these systems.

## Results and discussion

### Excited state lifetime from optical transient absorption spectroscopy

**Fig. 3:**
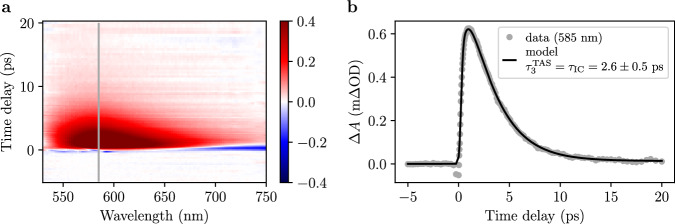
Transient optical absorption spectrum of HTI-J in acetonitrile. **a** The spectrum shows the change in optical density (ΔOD) as a function of time delay following excitation at 490 nm. The vertical line corresponds to the time trace shown in (b). **b** Kinetic trace of the transient absorption spectrum at 585 nm, around the maximum of the excited state absorption, and corresponding trace of a kinetic model globally fitted to the data. A decay component with time constant of 2.6  ±  0.5 ps is assigned to the lifetime of the charge transfer excited state of HTI-J in acetonitrile.

Femtosecond optical TAS provides information on the excited state population kinetics of HTI-J in acetonitrile, supporting the analysis and interpretation of the structurally sensitive TR-XSS measurements. Figure 3a shows the transient absorption spectrum as a function of the probe wavelength and time delay, recorded by exciting the sample at 490 nm, close to a maximum of the static absorption spectrum of HTI-J in acetonitrile (see Suppl. Note 2).

Data below 530 nm are affected by scattering of the pump light and are not considered in the analysis. The transient spectrum displays a broad excited state absorption band from around 540 to 690 nm that decays within 10 ps, and a shorter lived stimulated emission feature from around 670 to 750 nm, the red edge of the probe range. These features are consistent with those previously observed by Wiedbrauk et al.^4,50^ in transient absorption spectroscopy of HTI-J in polar solvents. The TA spectrum is found to be well described by a kinetic model consisting of a sum of three exponential decay components convolved with the instrument response function (IRF) and a constant offset. Global fitting with this kinetic model yields two spectral components that decay rapidly with time constants ${\tau }_{1}^{{\mathrm{TAS}}}=0.5\,\pm \,0.3$ ps and ${\tau }_{2}^{{\mathrm{TAS}}}=1.2\,\pm \,0.7$ ps, and a slower component decaying with ${\tau }_{3}^{{\mathrm{TAS}}}=2.6\,\pm \,0.5$ ps that accounts for most of the excited state absorption feature dominating the spectrum (more details on the TAS analysis can be found in Suppl. Note 3). Figure 3b shows a time trace of the spectrum for a wavelength of 585 nm, around the maximum of the excited state absorption, together with the corresponding trace of the fitted model. DFT calculations predict that excitation of HTI-J in acetonitrile occurs directly to the lowest excited state, which has a charge transfer character (see Suppl. Note 9.2). Based on this and following Wiedbrauk et al.’s assignment of TA spectral components of HTI compounds^4,50^, the fast kinetics are attributed to structural and vibrational relaxation of the solute in the excited state and decay of stimulated emission, while the 2.6  ±  0.5 ps decay is interpreted as the time *τ*_IC_ for internal conversion to the ground state, i.e., the excited state lifetime. The offset likely accounts for a minor population of the *E* isomer through photoisomerization^4,50^.

### Structural dynamics revealed by time-resolved X-ray solution scattering

The time-resolved X-ray scattering signal of a solution of HTI-J in acetonitrile was measured in a laser pump X-ray probe experiment at the SwissFEL XFEL to directly track the structural dynamics after photoexcitation. In the TR-XSS experiments, the ensemble of HTI-J molecules in acetonitrile was probed with X-ray pulses of 11 keV mean photon energy, either with (laser-on) or without (laser-off) prior excitation at 470 nm, corresponding to the maximum of the absorption spectrum, and an excitation fluence of approximately 1.2 mJ/mm^−2^, corresponding to the upper end of the linear-response region determined via a laser fluence scan (see Suppl. Note 4.1). Scattering patterns were recorded on the 2D detector as a function of the magnitude of the scattering vector, *q*, and the azimuthal angle, *ϕ*. Difference scattering images Δ*S*(Δ*t*, *q*, *ϕ*) = *S*_on_(Δ*t*, *q*, *ϕ*) − *S*_off_(*q*, *ϕ*) were then obtained by subtracting laser-off from laser-on images at each time delay Δ*t* between pump and probe pulse and decomposed into isotropic and anisotropic components via Legendre decomposition, yielding 1D difference scattering curves^52,53^. Further details on the reduction of the scattering data are provided in the Methods section and Suppl. Note 4.2, which includes plots of the reduced isotropic and anisotropic difference scattering signals.

The time-resolved isotropic difference scattering signal is analyzed using a stepwise global fitting approach that builds on global analysis strategies commonly used in time-resolved spectroscopy^50,54^. The difference scattering data are fitted with a bilinear model of the signal plus background artifact components (see Suppl. Note 4.6):

1 $$\Delta {S}^{{\mathrm{fit}}}(\Delta t,q)	=\Delta {S}^{{\mathrm{model}}}(\Delta t,q)+\Delta {S}^{{\mathrm{bkg}}}(\Delta t,q)\\ 	={\sum }_{k}{A}_{k}(\Delta t;\{{a}_{i}\},\{{\tau }_{i}\}){C}_{k}(q)+{\sum }_{l}{B}_{l}(\Delta t){D}_{l}(q).$$

Here, *A*_*k*_ are kinetic profiles consisting of rising and decaying exponential functions parameterized with a set of amplitudes {*a*_*i*_} and time constants {*τ*_*i*_} and convolved with the IRF, *C*_*k*_ are the corresponding scattering *q*-profiles, while *B*_*l*_ and *D*_*l*_ are amplitudes and corresponding *q*-profiles of background (bkg) components. The global fitting approach is described in detail in the Methods section and Suppl. Note 6. Briefly, in sequential steps, the amplitudes of the background components, *B*_*l*_, as well as the kinetic profiles and corresponding *q*-profiles, *A*_*k*_ and *C*_*k*_, are optimized based on linear least squares minimization^54,55^.

**Fig. 4:**
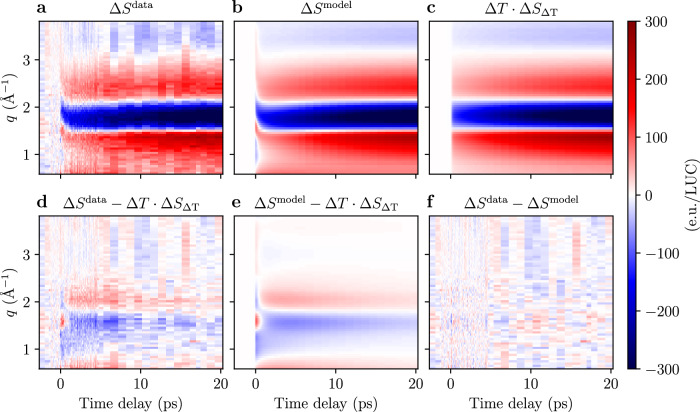
Time-resolved isotropic difference scattering data and model. **a** Time-resolved isotropic difference scattering signal of the organic HTI-J chromophore photoexcited in acetonitrile, with background removed, in electron units per liquid unit cell (e.u./LUC; see Suppl. Note 4.5). **b** Best-fit model difference scattering signal including contributions from (i) optical Kerr effect (OKE), (ii) solvation-shell structural rearrangement (SSR), (iii) vibrational relaxation (VR) of the solute, and (iv) bulk solvent heating (see eq. 2). **c** The bulk solvent heating contribution, which dominates the signal. **d** Time-resolved isotropic difference scattering signal after subtraction of the bulk solvent heating, highlighting the underlying contributions. **e** The heat-subtracted model including contributions (i-iii). The respective *q*-profiles are shown in Fig. 5 together with associated kinetic profiles and schematics of the corresponding molecular processes. Heat-subtracted difference signals and model contributions at selected time delays are shown in Suppl. Fig. 23. **f** Subtracting the model from the data results in a relatively flat residual apart from random noise.

Figure 4a shows the isotropic difference scattering signal of HTI-J in acetonitrile, Δ*S*^data^, after subtraction of the background components obtained in the global fitting.

The onset of the signal following photoexcitation at time zero can be clearly seen. The signal is noticeably dominated by two positive features, around 1.4 and 2.4 Å^−1^, and a negative feature, around 1.8 Å^−1^, which evolve on sub-picosecond and picosecond timescales. These features are consistent with the scattering changes arising from a temperature increase in bulk acetonitrile at constant volume, resulting from dissipation of energy from the photoexcited HTI-J solute^56^. However, the shape of the signal in the first picosecond after photoexcitation deviates from that of bulk solvent heating, indicating the presence of signal from additional structural processes.

Within the global fitting analysis, the measured difference scattering signal, Δ*S*^data^, is found to be well reproduced by a linear combination of four physically justified signal contributions (see Suppl. Note 5):

2 $$\Delta {S}^{{\mathrm{model}}}(\Delta t,q)=	\alpha (\Delta t,{\tau }_{{\rm{OKE}}})\Delta {S}_{{\rm{OKE}}}(q)\\ 	+\beta (\Delta t,{\tau }_{{\rm{SSR}}}^{1},{\tau }_{{\rm{SSR}}}^{2})\Delta {S}_{{\rm{SSR}}}(q)\\ 	+\gamma (\Delta t,{\tau }_{{\rm{VR}}}^{1},{\tau }_{{\rm{VR}}}^{2})\Delta {S}_{{\rm{VR}}}(q)\\ 	+\Delta T(\Delta t,{\tau }_{\Delta {{\rm{T}}}}^{1},{\tau }_{\Delta {{\rm{T}}}}^{2})\Delta {S}_{\Delta {{\rm{T}}}}(q).$$

**Fig. 5:**
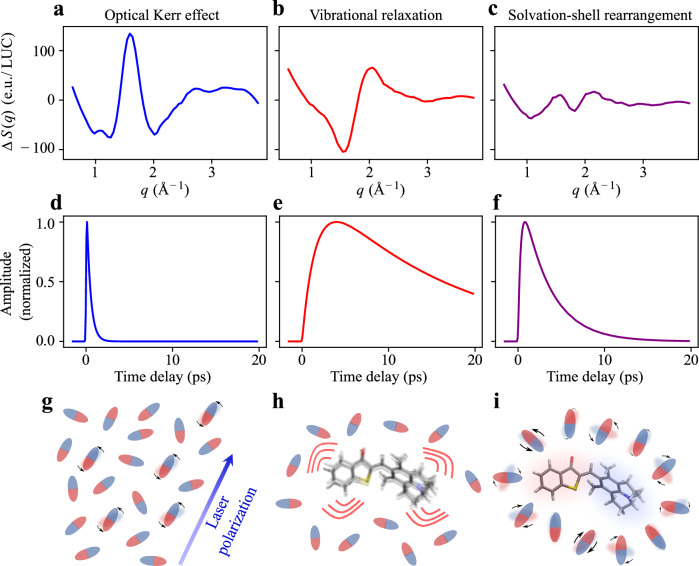
Results of a global fitting of the TR-XSS data and schematics of the molecular processes. **a**–**c** Optimized *q*-profiles in electron units per liquid unit cell (e.u./LUC) of kinetic components obtained from a global fitting of the time-dependent isotropic difference scattering signal of HTI-J photoexcited in acetonitrile, scaled by the fitted amplitudes. **d**–**f** Associated kinetic profiles. **g**–**i** Illustrations of the molecular processes assigned to each component, with the nitrogen and methyl ends of acetonitrile molecules colored in red and blue, respectively. **g** The optical Kerr effect arises through alignment of solvent molecules along the polarization axis of the electric field of the pump laser pulse. Translational motion of the solvent molecules to restore equilibrium results in the observed isotropic signal^61^. **h** Vibrational heating of the solute during internal conversion to the ground state leads to solvent rearrangements. **i** Photoexcitation of HTI-J leads to a structural rearrangement of the solvation shell in response to intramolecular charge transfer.

According to the assignment described in detail below, these four contributions represent (i) solvent motions associated with the optical Kerr effect (OKE), (ii) solvation-shell structural rearrangement (SSR) driven by changes in the electronic structure of the excited solute, (iii) solvent rearrangements due to vibrational relaxation of the solute (VR), and (iv) bulk solvent heating due to a temperature increase of Δ*T*. *α*, *β*, *γ*, and Δ*T* with associated time constants *τ* define the kinetic profiles of the four signal components. The resulting best-fit model scattering signal is shown in Fig. 4b, while the best-fit time constants are reported in Table 1. A plot of the optimized kinetic profiles is provided in Fig. 5d-f and Suppl. Note 6.2.

**Table 1:** Time constants from global fitting of the TR-XSS and TAS data

*τ*_OKE_	0.4 ± 0.1	Solvent structural changes due to optical Kerr effect
${\tau }_{{\mathrm{SSR}}}^{1}$	0.3 ± 0.1	Solvation-shell structural rearrangement (excited state)
${\tau }_{{\mathrm{SSR}}}^{2}$	3 ± 1	Solvation-shell structural reequilibration
*τ*_IC_	2.6 ± 0.5	Internal conversion from excited to ground state^*a*^
${\tau }_{{\mathrm{VR}}}^{1}$	1.6 ± 0.3	Solute vibrational heating
${\tau }_{{\mathrm{VR}}}^{2}$	15 ± 2	Solute vibrational cooling
${\tau }_{\Delta {\mathrm{T}}}^{1}$	0.2 ± 0.1	Fast solvent heating
${\tau }_{\Delta {\mathrm{T}}}^{2}$	13.5 ± 0.5	Slow solvent heating

Time constants (in ps) obtained from a global fitting of the model in eq. (2) to the time-resolved isotropic difference scattering signal of the HTI-J molecule photoexcited in acetonitrile and internal conversion time constant from transient absorption spectroscopy. The assignment of the dynamics is discussed in the next sections.

^*a*^From optical transient absorption spectroscopy measurements.

#### Solvent heating, vibrational relaxation, and optical Kerr effect

The contribution of the bulk solvent heating, Δ*T* ⋅ Δ*S*_ΔT_, is shown in Fig. 4c. It accounts for the majority of the observed signal after the first picosecond. The kinetics of this component are best described by a double exponential rise. The initial rise has a time constant of 0.2  ±  0.1 ps. Since the TAS measurements reveal an internal conversion time of *τ*_IC_ = 2.6  ±  0.5 ps, the rapid onset reflects energy transfer from the solute in the excited state. We conclude that this rapid solvent response arises from fast non-radiative relaxation following multiphoton excitation of a fraction of molecules to higher-lying states, as discussed in more detail in Suppl. Note 8. A similar fast rise of the solvent temperature was also observed in a recent TR-XSS study of a photoexcited metal complex in acetonitrile^38^. Except for the rapid onset of the heating signal (${\tau }_{\Delta {\mathrm{T}}}^{1} \sim 0.2$ ps) no contributions from the higher laser fluence in the TR-XSS experiments compared to the TAS could be discerned in our analysis. The second, slower exponential rise has a time constant of ${\tau }_{\Delta {\mathrm{T}}}^{2}=13.5\,\pm \,0.5$ ps. This component is attributed to bulk solvent heating resulting from vibrational cooling of the solute in the ground state and is consistent with the decay time of the vibrational relaxation component reported in Table 1 and discussed below. Nonequilibrium MD simulations of the vibrational cooling of the solute and heating of the solvent performed by vibrationally exciting the HTI-J molecule in the ground electronic state give a slow exponential rise of the solvent kinetic energy of  ~ 14.2 ps (see Suppl. Note 10.2), in good agreement with the experimental observation.

Figure 4d shows the time-resolved isotropic difference scattering signal after subtraction of the background artifact and bulk solvent heating components, highlighting the underlying signal contributions. This signal is compared to the sum of the remaining components of the model in Fig. 4e. The corresponding *q*-profiles for each of the remaining components are presented in Fig. 5a-c together with associated kinetic profiles in Fig. 5d-f and schematics of the assigned molecular processes in Fig. 5g-i. Heat-subtracted isotropic difference scattering traces at selected delay times, chosen to highlight where specific processes dominate, are shown in Suppl. Fig. 23 together with the corresponding individual model components.

As can be seen in Figs. 4d-e and 5b, the time-resolved isotropic difference scattering signal contains a component with a negative feature around *q* = 1.5 Å^−1^ and positive features around *q* = 2 Å^−1^ and below *q* = 1 Å^−1^, which rises in the first few picoseconds and then decays on a longer timescale. Global fitting including a single-exponential rise and decay for this component yields time constants of ${\tau }_{{\mathrm{VR}}}^{1}=1.6\,\pm \,0.3$ ps and ${\tau }_{{\mathrm{VR}}}^{2}=15\,\pm \,2$ ps, respectively. Since the decay time is comparable to the rise time of the bulk solvent heating signal, this component is attributed to structural rearrangements due to vibrational relaxation of the photoexcited solute. While the solute is vibrationally hot, it displaces or excludes the solvent from a larger volume compared to thermal equilibrium. This leads to changes in the solvent-solvent atom distances, which are reflected in an increase of the scattering signal at low-*q*^41,57^. The positive and negative peaks at higher *q* are assigned to a shift of the liquid peak to higher *q*, consistent with a local density increase of the acetonitrile solvent around the solute. The initial rise of this component reflects an increase of the excluded volume during internal conversion, consistent with vibrational heating of the solute and molecular dynamics simulations indicating a smaller excluded volume in the excited state relative to the ground state (see Suppl. Note 10.1). The decay time of 15  ±  2 ps is assigned to vibrational cooling in the ground state. This is in agreement with previous time-resolved optical and infrared transient absorption studies of organic molecules in acetonitrile, which report vibrational cooling times of 10-15 ps, largely independent of the solute^58–60^.

The fast decaying component visible before 1 ps after subtraction of the background and bulk solvent heating component, with a prominent peak around *q* = 1.6 Å^−1^, has a shape (see Fig. 5a) similar to that attributed by Ki et al.^61^ to structural dynamics in liquid acetonitrile triggered by the optical Kerr effect. The electric field of the pump laser induces photoalignment of solvent molecules, and the subsequent structural relaxation through translational motions to restore equilibrium produces an isotropic scattering signal^61^. Here, the OKE contribution to the time-dependent isotropic X-ray scattering signal of HTI-J in acetonitrile is found to decay with a time constant of *τ*_OKE_ = 0.4  ±  0.1 ps, in agreement with the value of 0.35  ±  0.21 ps reported by Ki et al.^61^ for an azo dye solution.

#### Solvation dynamics

Figure 5c shows the *q*-profile of the component of the global fitting of the TR-XSS data attributed to solvation-shell structural rearrangement (SSR) upon photoexcitation of HTI-J in acetonitrile. The presence of the SSR contribution can be inferred from the modulation of the shape of the difference scattering signal on the  < 10 ps timescale (see, e.g., the negative feature around *q* = 1 Å^−1^ in Fig. 4d). Although small in amplitude, this contribution is found to be necessary for adequately reproducing the observed time-dependent scattering signal, as confirmed by analysis of the residual of a model including only contributions of bulk solvent heating and vibrational relaxation (see Suppl. Note 5.4).

**Fig. 6:**
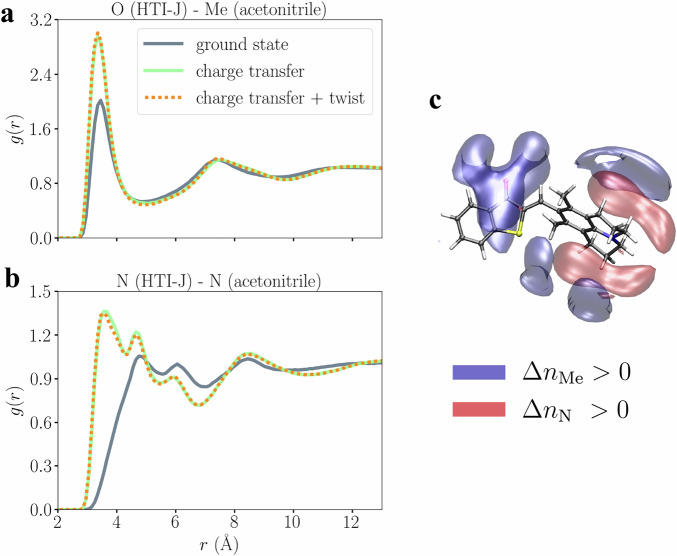
Results of molecular dynamics simulations of HTI-J in acetonitrile. **a**, **b** Solute-solvent radial distribution functions calculated from molecular dynamics simulations of HTI-J in acetonitrile for (i) the ground state, (ii) the excited state with changed partial charges and ground state geometry, and (iii) the excited state with both partial charges and geometry changed. **c** Positive difference in number density (*Δn*) for the methyl and N sites of acetonitrile between the excited state with only partial charges changed and the ground state. The isosurface corresponds to an average increase of one atom. The simulations predict a dipolar solvation-shell reorganization driven by intramolecular charge transfer.

The assignment of the SSR component is supported by DFT calculations and MD simulations (see Computational methods). The DFT calculations predict that excitation leads to a twist around the single bond that connects the thioindigo with the julolidine groups from  ~ 60^∘^ to  ~ 90^∘^, and a change in the magnitude of the dipole moment of 26.3 D, corresponding to the transfer of  ~ 0.7 electrons from the julolidine to the thioindigo group (see Suppl. Note 9.2). Figure 6a-b show representative radial distribution functions (RDFs) obtained from equilibrium MD simulations in both the ground and excited state, with force-field parameters for the solute derived from the DFT calculations. Figure 6c shows isosurfaces of the change in number density of methyl and nitrogen sites of the solvent molecules obtained from the simulations. The plots show that upon photoexcitation the average density of solvent methyl sites increases around the thioindigo fragment, which gains electron density upon excitation. Conversely, around the julolidine fragment, there is an increase in the density of nitrogen atoms. These results illustrate how the dipoles of the acetonitrile molecules reorient to adapt to the changes in electric field arising from the intramolecular charge transfer. Excited state MD simulations where only the partial charges are changed in the excited state compared to the ground state give very similar results, indicating that the observed solvation-shell reorganization is dominated by the response to charge transfer, rather than rearrangements due to twisting of the chromophore. Key structural parameters of the solvation shell in the ground and excited states computed from the solute-solvent RDFs are reported in Suppl. Note 10.1.

**Fig. 7:**
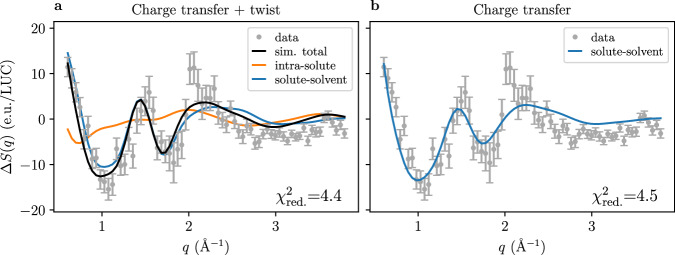
Comparison between experimental and simulated signal. The gray dots represent the average between 0 and 5 ps of the difference scattering signal in electron units per liquid unit cell (e.u./LUC) of HTI-J photoexcited in acetonitrile after subtracting the contributions from background artifacts, bulk solvent heating, vibrational relaxation and the signal response to the optical Kerr effect. The error bars represent the standard deviation of a third order polynomial fit in a 16 points interval around each data point^78^ (see Suppl. Note 4.7). **a** Comparison with a simulated curve (black) consisting of two contributions obtained from intra-solute (orange) and solute-solvent (blue) radial distribution functions of molecular dynamics simulations with excited state charge transfer and twisting. **b** Comparison with a solute-solvent simulated curve (blue) obtained from molecular dynamics simulations with excited state charge transfer only. In each case, the simulated curve is fitted to the experimental signal. The close agreement with the solute-solvent simulated curve shows that the time-resolved X-ray scattering signal directly reflects the reorganization of solvent molecules driven by charge transfer excitation, i.e., the polar solvation dynamics. The quality of the fit is similar in both cases, indicating that the current signal-to-noise ratio prevents a quantitative determination of the solute structural change.

Figure 7 shows an average of the experimental data between 0 and 5 ps after subtracting contributions from background artifacts, bulk solvent heating, vibrational relaxation and the signal response to the optical Kerr effect obtained from global fitting with charge transfer and twist as initial guess for the SSR component (see Suppl. Fig. 24). This signal is compared to a difference scattering signal obtained from the MD simulations where both the molecular geometry and partial charges are changed in the excited state (Fig. 7a), and where only the partial charges are changed in the excited state while the molecular geometry is the same as in the ground state (Fig. 7b).

The simulated signal in Fig. 7a consists of two contributions, one due to changes in intramolecular distances obtained from the intra-solute RDFs, and one due to changes in solute-solvent atom pair distances obtained from the solute-solvent RDFs. The latter, often referred to as cross term, represents solvent-shell rearrangements encoded in variations of solute-solvent distances. Over almost the entire *q*-range up to around 3 Å^−1^, the solute-solvent term dominates over the intra-solute term. The simulated signal shown in Fig. 7b includes only a solute-solvent term due to the solvent response to charge transfer. As expected based on the comparison of RDFs in Fig. 6, the solute-solvent simulated signal due to charge transfer and twisting and due to only charge transfer are very similar. The average experimental signal closely matches the simulated signal, with both models giving a similar quality of the fit (reduced *χ*^2^ = 4.4 and 4.5, respectively). For charge transfer and twisting, the fit is marginally improved between 2 to 3 Å^−1^, but the signal-to-noise ratio does not allow for quantitative discrimination between the two models, meaning that the current data can resolve only the transient solvation-shell structure, not the transient solute structural changes.

As the simulated signals for charge transfer with twisting and charge transfer only are similar (see Suppl. Note 10.1), comparable results are obtained when the latter is used as initial guess for the SSR component in the global fitting (see Suppl. Fig. 24). In contrast, simulations accounting only for the geometric rearrangement of the solute without charge transfer fail to reproduce the experimental SSR component, even when the simulated signal is used as an initial guess for the global fitting (Suppl. Fig. 25). Altogether, these results show the observed solvation-shell reorganization is primarily driven by charge transfer, rather than the rearrangement due to twisting of the chromophore. The structural rearrangements in the solvation shell underlying the observed scattering signal are analyzed in greater detail in Suppl. Note 10.1 through a quantitative analysis of the calculated solute-solvent RDFs.

Having established the origin of the SSR component, we finally turn to the interpretation of the observed kinetics. The kinetic model yields a single-exponential rise with best-fit time constant of ${\tau }_{{\mathrm{SSR}}}^{1}=0.3\,\pm \,0.1$ ps and a decay with ${\tau }_{{\mathrm{SSR}}}^{2}=3\,\pm \,1$ ps, respectively. The first time constant corresponds to the rise of the SSR signal due to solvent reorganization driven by photoinduced intramolecular charge transfer of the solute, lowering the free energy of solvation. Measurements of the dynamic Stokes shift^14,25^ and previous MD simulations^18,23^ found that the minimization of the free energy of solvation following intramolecular charge transfer in dipolar solvents has a biexponential time evolution. These timescales were attributed to an initial ultrafast response, driven by the torque exerted by charge redistribution on nearby solvent molecules, and a slower, diffusive reorganization of the solvent, respectively. In the case of acetonitrile, time-resolved emission measurements of coumarin 153, a molecule that also exhibits a significant change in dipole moment upon photoexcitation, yield timescales of approximately 0.09 ps for the initial, fast response and around 0.6 ps for the slower diffusive motion, with an average solvation time of around 0.25 ps^23,25^. Due to a time resolution of  ~ 120 fs full width at half maximum (FWHM) and limited *q*-range of the TR-XSS measurements, only a single time constant of 0.3  ±  0.1 ps for the solvation-shell rearrangement could be resolved in the present study, in agreement with the average timescale reported in the previous studies on coumarin 153^23,25^. The slower time constant of 3  ±  1 ps reflects the reequilibration of the solvation-shell structure as the excited state population undergoes internal conversion returning to the ground state. This is supported by the close agreement with the excited state lifetime of 2.6  ±  0.5 ps obtained from the TAS measurements.

Overall, the TR-XSS measurements capture with atomic resolution both the ultrafast reorganization of acetonitrile molecules around the photoexcited HTI-J compound and the subsequent reequilibration as the system relaxes to the ground state. The solvent shell reorganization is found to be governed by the response to intramolecular charge transfer, while the response to the structural change of the solute is minor. This polar solvation dynamics provide compelling evidence for the formation of a charge transfer excited state in electron donor substituted HTIs in polar solvents, which has previously been proposed based on the indirect observation of shifts in time-resolved optical and vibrational spectra^4,49^.

### Summary of results and outlook

The nonequilibrium response of a polar solvent to photoinduced charge transfer in organic molecules is widely recognized as a key factor in stabilizing transient charge separated states and modulating photorelaxation pathways^5,10,14^. Time-resolved optical experiments, such as measurements of the dynamic Stokes shift, have been used to probe the timescales of the change in solvation free energy associated with polar solvation dynamics^8,25,26^. However, the transient reorganization of polar solvent molecules around a purely organic chromophore as charge is redistributed upon photoexcitation has so far not been observed with atomic resolution.

Here, we have exploited the structural sensitivity of the time-resolved X-ray solution scattering technique with a  ~ 120 fs time resolution and the high X-ray photon flux at SwissFEL to resolve transient changes in the solvation shell of an organic hemithioindigo compound photoexcited in acetonitrile. Upon excitation, intramolecular charge transfer occurs from the julolidine to the thioindigo group, inducing a dipolar rearrangement of the surrounding solvent molecules. The TR-XSS measurements capture the formation of the transient solvation-shell structure on a  ~ 0.3 ps timescale and its reequilibration as the system returns to the ground state with a time constant of  ~ 2.6 ps. The X-ray scattering signature of the transient solvation-shell reorganization was separated from the bulk solvent heating and other contributions, and compared with the scattering signal predicted by MD simulations, revealing that the solvent reorganizes predominantly in response to the intramolecular charge transfer rather than to the atomic structural change of the excited solute structure.

In the present study, disentangling the solvation dynamics component in the TR-XSS data is facilitated by the large photoinduced change in the dipole moment of the HTI-J molecule (~ 27 D at the relaxed excited state geometry), which triggers a correspondingly large restructuring of the solvation shell. The experiments provide a clear atomic-scale signature of the transient solvation shell. However, neither the time evolution of specific solvent motions underlying the solvation-shell rearrangement nor any accompanying solute structural change could be resolved. As the repetition rate, temporal resolution, and X-ray photon energy available at XFEL facilities continue to improve^62–65^, and as robust analysis methods^52,57,66–69^ and advanced multiscale simulation techniques^70–72^ are developed, we anticipate that ultrafast X-ray scattering will increasingly provide direct, atomically resolved observations of coupled solute-solvent dynamics involving light atoms across a broad range of photoreactions, including the identification of the specific atomic motions involved. Moreover, while in the present study the photoinduced charge transfer is inferred indirectly through the solvent response, we anticipate that the aforementioned advancements will make it possible to achieve direct X-ray scattering observations of pure electronic rearrangements in solution, as already demonstrated for isolated molecules^73–75^. It will then be possible to achieve detailed mechanistic insights into the solvent influence on photoinduced processes and, ultimately, more effective control and exploitation of charge and atomic motion in complex molecular systems.

## Methods

### Sample synthesis and handling

HTI-J was synthesized as detailed in Suppl. Note 1. The synthesized compound was transported to the experimental facilities in powder form, where it was dissolved in acetonitrile to prepare sample solutions for the measurements. To prevent photoisomerization of HTI-J under ambient light, the solute samples were stored in the dark and handled exclusively under red light.

### Optical transient absorption spectroscopy

Femtosecond optical transient absorption spectroscopy measurements were carried out at the Center for Nanoscale Materials at the Argonne National Laboratory using a commercially available femtosecond laser system (Spectra-Physics) and spectrometer (Ultrafast Systems). Pump and probe pulses were delivered by an amplified Ti:sapphire laser capable of providing pulses with a width of 80 fs. An excitation wavelength of 490 nm was achieved through the use of an optical parametric amplifier and a white-light continuum in the visible range was used to probe the sample solution of HTI-J in acetonitrile with a concentration of 0.3 mM. The measurements were performed with the sample contained in a 2 mm quartz cuvette and stirred continuously. The excitation power was set to 300 μW on a 200 μm spot size and the measurement was repeated with a rate of 2.5 kHz. 19 recorded spectra were averaged and corrected for chirp and time zero. Background signals were removed by subtracting the average before time zero. The borders and pump scatter region were cut and the remaining spectra were binned by a factor of two along the wavelength axis and globally fitted with a kinetic model including a sum of three exponential functions and an offset using the python package KiMoPack (version 7.12.14)^54^. Details on the analysis of the optical transient absorption spectra can be found in Suppl. Note 3.

### Time-resolved X-ray solution scattering

Laser pump, X-ray probe experiments were conducted at the Alvra endstation of the SwissFEL X-ray free electron laser. A 3 mM sample solution of HTI-J in acetonitrile was supplied by a liquid jet with a diameter of 100 μm and a flow speed sufficient to replenish the sample for each pump-probe event at 100 Hz repetition rate. An optical laser with an excitation wavelength of 470 nm and focused to a spot size of 73 × 95 μm (FWHM) was used to excite HTI-J at the absorption maximum (*Z* isomer) with a laser energy of 12 μJ per pulse (for a laser fluence scan see Suppl. Note 4.1). Probing of the sample solution after a variable time delay Δ*t* was achieved with X-ray radiation with a photon energy of 11 keV and a pulse energy of around 1 mJ/pulse focused to a spot size of 30 × 30 μm (FWHM). A 6:1 pulse scheme was used, meaning that every seventh measurement was performed without prior laser excitation. A Jungfrau 16M two-dimensional detector in fixed gain mode with medium gain for the center modules and high gain for the edge modules was used to record the forward scattering at a sample-detector distance of approximately 9.7 cm, which was calibrated using a LaB6 reference measurement. The arrival time of the X-ray pulse relative to the optical laser was monitored by a timing tool^76^. Suppl. Table 2 summarizes key experimental parameters. The acquired raw 2D scattering images were corrected for the setup geometry and masked. Images deviating from the median measurement conditions were rejected based on an intensity filter and the correlation between the measured intensity and an I0 intensity monitor, resulting in rejection of approximately 30% of the images. The remaining images were integrated in 13 azimuthal bins^52,77^, normalized and the difference scattering signal was calculated by subtracting laser-off signals obtained without prior laser excitation from each laser-on signal. The generated difference scattering signals were re-binned into equistatistical time bins based on the timing tool information and averaged. The averaged difference scattering signal was decomposed into its isotropic and anisotropic components^52,53^ and scaled to the smallest stoichiometrically representative unit, the liquid unit cell (LUC). A detailed description of the data reduction can be found in Suppl. Note 4. The analysis presented here focuses on the isotropic difference scattering signal, which is referred to as Δ*S*(Δ*t*, *q*) throughout the article.

### Global fitting of time-resolved X-ray solution scattering data

After reduction, the time-resolved isotropic difference scattering signal is given as a function of a set of *M* values of time delay {Δ*t*_1_,  Δ*t*_2_,  …,  Δ*t*_*m*_} and *N* values of scattering vector magnitude {*q*_1_,  *q*_2_,  …,  *q*_*n*_}. A stepwise global fitting procedure was used to analyze the data, employing a bilinear model of the signal plus background (bkg) artifact components (see also eq. (1):

3 $$\Delta {S}^{{\mathrm{fit}}}(\Delta {t}_{m},{q}_{n})	=\Delta {S}^{{\mathrm{model}}}(\Delta {t}_{m},{q}_{n})+\Delta {S}^{{\rm{bkg}}}(\Delta {t}_{m},{q}_{n})\\ 	={\sum }_{k}{A}_{k}(\Delta {t}_{m};\{{a}_{i}\},\{{\tau }_{i}\}){C}_{k}({q}_{n})+{\sum }_{l}{B}_{l}(\Delta {t}_{m}){D}_{l}({q}_{n}).$$

In matrix form,

4 $$\Delta {{{\bf{S}}}}^{{\mathrm{fi}}t}=\Delta {{{\bf{S}}}}^{{\rm{model}}}+\Delta {{{\bf{S}}}}^{{\rm{bkg}}}={{\bf{A}}}\cdot {{\bf{C}}}+{{\bf{B}}}\cdot {{\bf{D}}},$$

where **A** is an *M* × *K* matrix with columns representing *K* kinetic profiles, dependent on amplitudes *a*_*i*_ and time constants *τ*_*i*_ and convolved with the IRF, **C** is a *K* × *N* matrix with rows containing the corresponding scattering profiles, **B** is an *M* × *L* matrix of time-dependent amplitudes for *L* background components with *q*-profiles contained in the rows of the *L* × *N* matrix **D**. Suppl. Note 7 describes the kinetic functions used in the model, while no kinetic form is assumed for the amplitudes of the background components. In the global fitting, **A,** **B** and **C** are optimized by minimizing the square of the residual between the data and the fit function, while the background artifact profiles contained in **D** are fixed to left singular vectors of an SVD of the difference scattering data at negative time delays (see Suppl. Note 4.6).

First, both the kinetic profiles of the model and the amplitudes of the background components are optimized by solving

5 $${\min }_{{{\bf{A}}}}{\min }_{{{\bf{B}}}}{\bigg|\bigg| {{\mathcal{L}}}\left(\frac{\Delta {{{\bf{S}}}}^{{\rm{data}}}-{{\bf{A}}}\cdot {{{\bf{C}}}}_{{{\bf{0}}}}-{{\bf{B}}}\cdot {{\bf{D}}}}{\sigma }\right)\bigg|\bigg|}^{2},$$

where **C**_**0**_ contains initial *q*-profiles that are kept fixed in this step and *σ* is the noise estimated using a piecewise polynomial fitting^78^ (see Suppl. Note 4.7). ${{\mathcal{L}}}$ denotes the loss function. In the present global fitting, the more outlier-resistant arctan loss function was used. The model includes four signal components (see eq. (2)). The initial *q*-profile for the component representing bulk solvent heating is obtained as an average of the experimental data after 55 ps, Δ*S*_ΔT_ = < Δ*S*^data^ > _55<Δ*t*<60ps_, when the signal has stabilized, indicating that the solute has dissipated all excess energy, reaching thermal equilibrium in the ground state. The initial *q*-profile of the other components is obtained either from an SVD of the data (optical Kerr effect and vibrational relaxation terms, Δ*S*_OKE_ and Δ*S*_VR_), or as the difference scattering signal calculated from intra-solute and solute-solvent RDFs of equilibrium MD simulations (solvation-shell structural rearrangement term, Δ*S*_SSR_), as described in more detail in Suppl. Notes 5 and 10.1.

In a second step, the *q*-profiles are optimized by solving

6 $${\min }_{{{\bf{C}}}}\{\parallel \Delta {{{\bf{S}}}}^{{\prime} }-{{\bf{A}}}\cdot {{\bf{C}}}{\parallel }^{2}+\parallel {{\bf{W}}}\cdot ({{\bf{C}}}-{{{\bf{C}}}}_{0}){\parallel }^{2}\},$$

where $\Delta {{{\bf{S}}}}^{{\prime} }=\Delta {{\bf{S}}}-{{\bf{B}}}\cdot {{\bf{D}}}$ are the data after subtraction of the background components obtained in the first step. The optimization can be regularized by penalizing deviations from the initial *q*-profiles through the term $\parallel {{\bf{W}}}\cdot ({{\bf{C}}}-{{{\bf{C}}}}_{0}){\parallel }^{2}$, where **W** is a diagonal matrix with the square roots of the regularization parameters on the diagonal (see Suppl. Note 6.1 for more details). In the present analysis, the *q*-profile of the bulk solvent heating is fully constrained as the average at late time delays is expected to accurately represent this contribution, while no constraint was placed on the other components.

In the final step, the kinetic profiles are reoptimized by fitting the data after subtraction of the background components, while keeping the *q*-profiles fixed at the values obtained in the previous step:

7 $${\min }_{{{\bf{A}}}}{\bigg| \bigg| {{\mathcal{L}}}\left(\frac{\Delta {{{\bf{S}}}}^{{\prime} }-{{\bf{A}}}\cdot {{\bf{C}}}}{\sigma }\right)\bigg|\bigg|}^{2}.$$

### Computational methods

The DFT calculations used to obtain equilibrium geometries and partial charges for the solute in the MD simulations employ the range-separated hybrid functional CAM-B3LYP^79–81^ and an ma-def2-TZVP basis set^82,83^. Excited state calculations are performed using linear-response time-dependent DFT (TDDFT)^84^ as well as a time-independent density functional approach where the orbitals are variationally optimized for the excited state^85,86^. The orbital-optimized calculations are performed within the spin-unrestricted formalism by optimizing the orbitals after promotion of an electron from an occupied orbital of the ground state to an unoccupied one. These orbitals are identified as the orbitals of the electron-hole pair that contributes most significantly to the lowest excited state with significant oscillator strength as obtained in the TDDFT calculations (in this case, this state also corresponds to the lowest excited state overall). All DFT calculations include implicit solvent effects through the conductor-like polarizable continuum model (CPCM)^87^ for acetonitrile. Previous studies^19,23^ have shown that the excitation and solvation energy obtained with polarizable continuum models are consistent with the corresponding ensemble-averaged values from fully quantum-mechanical calculations including explicit solvent molecules. The structural parameters and partial charges of the HTI-J molecule in the excited state used in the MD simulations are obtained from the orbital-optimized calculations, since this approach has been shown to provide more accurate properties, such as excitation energy^88^ and dipole moment^89^, for intramolecular charge transfer excitations than TDDFT. To validate the DFT calculations, additional calculations were carried out using the domain based local pair-natural orbital (DLPNO) coupled cluster method with singles, doubles and perturbative triples (CCSD(T))^90,91^, including implicit solvent effects throught CPCM. All DFT and CCSD(T) calculations were performed using the ORCA software version 6.0^92,93^. Further aspects of the DFT and CCSD(T) calculations and the corresponding results are presented in Suppl. Note 9.

The MD simulations use the generalized Amber force field (GAFF)^94^. For the HTI-J molecule, equilibrium bond lengths, angles, and selected dihedral angles, as well as partial charges, are assigned based on the ground and excited state structures optimized relaxing the atomic forces described using DFT with the CAM-B3LYP functional and CPCM. The HTI-J molecule and the acetonitrile molecules are flexible in the simulations. To prevent non-bonding interactions to make the HTI-J single-bond twist angle deviate too much from the DFT equilibrium value, this angle is restrained by using larger force constants than the ones provided by GAFF. Other force constants are kept at the default GAFF values. The HTI-J partial charges are derived from fitting the molecular electrostatic potential obtained in the DFT calculations using the CHELPG method^95^. The MD simulation box includes one HTI-J molecule and 717 acetonitrile molecules. MD simulations with fixed-value partial charges have been shown to describe the orientational response of a polar solute to charge transfer in the solute^19,23^. Moreover, using a flexible solute with fixed partial charges has been shown to capture some polarization effects, since the solute can distort in response to solute-solvent interactions and thereby change its instantaneous and average dipole moment relative to the equilibrium geometry in an implicit solvent^23^.

Four sets of equilibrium MD simulations were performed, for the ground state and for three different models of the excited state: charge transfer and twist, only charge transfer, and only twist. In the first model, both the geometry and partial charges are changed in the excited state simulations. In the second, only the partial charges are changed while the ground state geometry is retained. In the third, only the geometry is changed while the ground state partial charges are retained. These simulations correspond to the HTI-J molecule equilibrated in the excited state with a fully formed solvation shell, and do not account for nonequilibrium solvent motions during the solvation shell rearrangement. First, a 5 ns NPT run at room temperature (298.15 K) and pressure of 1 atm was performed for the ground state to find the equilibrium volume of the box, resulting in a side length of approximately 40.1 Å. Using this box size, the system was further equilibrated in the NVT ensemble for 5 ns for the ground state and the three models of the excited state. For each system, after the equilibration, an NVT production run of 400 ns was performed, collecting one frame every picosecond, in total 400 000 equidistant frames. All of these frames were used in the subsequent calculations of the equilibrium RDFs. The timestep used was 1 fs in all equilibration runs and equilibrium MD simulations.

For the nonequilibrium MD simulations of the vibrational cooling of the HTI-J and the heating of the solvent, approximately 4000 frames were selected from the equilibrated NVT trajectory. The velocities of HTI-J atoms were then rescaled according to a Maxwell-Boltzmann distribution to achieve a total kinetic energy increase of 2.64 eV, corresponding to the photon energy of the laser pump pulse used in the TR-XSS experiments. Each nonequilibrium trajectory was then propagated in the NVE ensemble for 60 ps with a timestep of 0.5 fs. All MD simulations were performed using the OpenMM 8.2.0 software package^96^.

The RDFs were computed from the equilibrium and nonequilibrium NVT trajectories using the molecular graphics software VMD^97^. The scattering signals were then calculated from the RDFs as described in Dohn et al.^98^. Prior to calculation of the scattering signal, the RDFs are corrected for finite-size effects by applying the Ganguly and van der Vegt correction^57,99^ as well as a smooth step damping function with cutoff 17 Å as described by Zederkof et al.^15^ (equilibrium RDFs) or a 20 Å Lorch-like damping window function^100^ (nonequilibrium RDFs). The difference scattering signal was obtained by subtracting the scattering signal of the ground state from that of the excited state as calculated from ground and excited state RDFs, respectively.

## Supplementary information

Supplementary Information

Transparent Peer Review file

## Source data

Source Data

## Data Availability

Source data are provided with this paper. Large data sets are available in a Figshare repository^101^. The spectroscopy data, reduced X-ray scattering data, and simulation data generated in this study that are required to reproduce the findings are available in the Source Data files and in the Figshare repository. Raw X-ray scattering data generated at the SwissFEL facility are stored on the archive servers of the facility, and can be publicly accessed through Paul Scherrer Institute remote data services^102^. Source data are provided with this paper.

## Code availability

The analysis of the time-resolved X-ray solution scattering data uses custom code publicly available at https://gitlab.com/asod/gxssfit/-/tree/hti_swissfel_global_analysis and on Zenodo^103^.
