# Supplementary material for: Tracking polar solvation dynamics of a photoexcited organic chromophore with ultrafast X-ray scattering
Source: Nat Commun. 2026 Apr 13;17:5080. doi: 10.1038/s41467-026-71635-1 (PMC13247046; doi:10.1038/s41467-026-71635-1)
Supplement: Supplementary file 1 — Supplementary Information [file 41467_2026_71635_MOESM1_ESM.pdf]

Supplementary Information for: Tracking Polar Solvation Dynamics of a Photoexcited Organic Chromophore with Ultrafast X-ray Scattering

Kerstin M. Mitterer[1*], Elli Selenius[2], Morten L. Haubro[1], Magnus A. H. Christiansen[2], Verena Markmann[1], Bianca L. Hansen[1], Mikkel Krell-Jørgensen[3], Joseph G. F. Hoock[3], Victor Lorentzen[1], Emma V. Beale[4], Philip J. M. Johnson[4], David J. Gosztola[5], Claudio Cirelli[4], Camila Bacellar[4], Asmus O. Dohn[1], Luca Laraia[3], Klaus B. Møller[3], Kristoffer Haldrup[1], Gianluca Levi[2,6*†], Martin M. Nielsen[1*†]

[1]Department of Physics, Technical University of Denmark, 2800 Kongens Lyngby, Denmark.
[2]Science Institute and Faculty of Physical Sciences, University of Iceland, Reykjavík, Iceland.
[3]Department of Chemistry, Technical University of Denmark, 2800 Kongens Lyngby, Denmark.
[4]Paul Scherrer Institut, Villigen PSI, Switzerland.
[5]Center for Nanoscale Materials, Argonne National Laboratory, 9700 S Cass Ave, Lemont, Illinois 60439, United States.
[6]Department of Chemical and Pharmaceutical Sciences, University of Trieste, 34127 Trieste, Italy.

*Corresponding author(s). E-mail(s): kmitt@dtu.dk; giale@hi.is; mmee@fysik.dtu.dk;
†These authors contributed equally to this work

Supplementary Notes

1 Suppl. Note 1. Sample synthesis and preparation 3

2 Suppl. Note 2. Stationary absorption spectrum of HTI-J in acetonitrile 10

3 Suppl. Note 3. Optical transient absorption spectroscopy 10

4 Suppl. Note 4. Time-resolved X-ray solution scattering 13
4.1 Suppl. Note 4.1. Laser fluence scan 14
4.2 Suppl. Note 4.2. Reduction of TR-XSS data 15
4.3 Suppl. Note 4.3. Singular value decomposition of the anisotropic difference scattering signal 17
4.4 Suppl. Note 4.4. Time zero and time resolution 19
4.5 Suppl. Note 4.5. Liquid unit cell scaling 20
4.6 Suppl. Note 4.6. Identification of background artifact components . . . 22
4.7 Suppl. Note 4.7. Noise estimation 23

5 Suppl. Note 5. Identification of signal components in the TR-XSS data 24
5.1 Suppl. Note 5.1. Bulk solvent heating 24
5.2 Suppl. Note 5.2. Vibrational relaxation of the solute 26
5.3 Suppl. Note 5.3. Optical Kerr effect 27
5.4 Suppl. Note 5.4. Solvation-shell structural rearrangement 27

6 Suppl. Note 6. Global fitting of the TR-XSS data 29
6.1 Suppl. Note 6.1. Regularization 30
6.2 Suppl. Note 6.2. Global fitting results 31
6.2.1 Suppl. Note 6.2.1. Heat-subtracted difference signal at selected time delays 32
6.2.2 Suppl. Note 6.2.2. Charge transfer initial guess for SSR component 33
6.2.3 Suppl. Note 6.2.3. Twist-only initial guess for SSR component 35

7 Suppl. Note 7. Kinetic functions 35
7.1 Suppl. Note 7.1. Double exponential rise 36
7.2 Suppl. Note 7.2. Single exponential rise and decay 36
7.3 Suppl. Note 7.3. Step-function rise and single exponential decay 37

8 Suppl. Note 8. Energy deposition into the solvent 37

9 Suppl. Note 9. Electronic structure calculations 38
9.1 Suppl. Note 9.1. Basis set convergence 38
9.2 Suppl. Note 9.2. DFT calculations 39
9.3 Suppl. Note 9.3. CCSD(T) calculations 42

10 Suppl. Note 10. Molecular dynamics simulations 43
10.1 Suppl. Note 10.1. Equilibrium molecular dynamics simulations 43
10.2 Suppl. Note 10.2. Nonequilibrium molecular dynamics simulations . . 47

1 Suppl. Note 1. Sample synthesis and preparation

General directions

All reactions were run under a N_2 atmosphere unless otherwise specified and were monitored by thin layer chromatography (TLC) and/or reversed-phase ultra-performance liquid chromatography mass spectrometry (RP-UPLC-MS). Commercially available reagents were purified according to standard procedures or were used as received from Sigma Aldrich, Alfa Aesar, Acros Organics, Combi-Blocks, Fisher Scientific, Strem, and Merck. All solvents used were of High-Performance Liquid Chromatography (HPLC) quality and dry solvents (DCM, Et_2O, THF, and Toluene) were obtained from a Pure-Solv system (Innovative Technology, Tronyx). Methanol was stored over activated 3 Å molecular sieves before use. Analytical TLC was conducted on Merck aluminium sheets covered with silica (C60). The plates were either visualized under UV-light or stained by dipping in a developing agent followed by heating. $KMnO_4$ [$KMnO_4$ (3 g) in water (300 mL), K_2CO_3 (20 g) and 5% aqueous NaOH (5 mL)] and cerium molybdate [$Ce(NH_4)_2(NO_3)_6$ (0.5 g), $(NH_4)_6Mo_7O_{24}\cdot 4H_2O$ (24.0 g), and H_2SO_4 (24.0 g)] were used as developing agents. Flash column chromatography was performed using Merck Geduran® Si 60 (40-63 µm) silica gel.

All new compounds were characterized by Nuclear Magnetic Resonance (NMR), Mass Spectrometry (Electrospray Ionization) (MS (ESI)), and High-Resolution Mass Spectrometry (HRMS) (ESI) (byproducts were not fully characterized). Structural assignments were made when possible for new compounds using COSY (Correlation Spectroscopy), HSQC (Heteronuclear Single Quantum Coherence), NOESY (Nuclear Overhauser Effect Spectroscopy), HMBC (Heteronuclear Multiple Bond Correlation), H2BC (Heteronuclear Two Bond Correlation) spectra where appropriate. For the recording of ^1H NMR and ^{13}C NMR spectra, a Bruker Ascend with a Prodigy cryoprobe (operating at 400 MHz for proton and 100 MHz for carbon) was used. The chemical shifts (δ) are reported in parts per million (ppm) and the coupling constants (J) in Hz. Spectra were referenced using the residual solvent peaks of the respective solvent; DMSO (δ 2.50 ppm for ^1H NMR and δ 39.52 ppm for ^{13}C NMR), $CDCl_3$ (δ 7.26 ppm for 1H NMR and δ 77.16 ppm for ^{13}C NMR), CD_3OD (δ 3.31 ppm for ^1H NMR and δ 49.00 ppm for ^{13}C NMR). The following abbreviations were used to report peak multiplicities: s = singlet, d = doublet, t = triplet, q = quartet, dd = doublet of doublets, sept = septet, m = multiplet, bs = broad singlet. Analytical RP-UPLC-MS (ESI) analysis was performed on a S2 Waters AQUITY RP-UPLC system equipped with a diode array detector using an Thermo Accucore C18 column (d 2.6 µm, 2.1 x 50 mm; column temp: 50 °C; flow: 1.0 mL/min). Eluents A (0.1% HCO_2H in H_2O) and B (0.1% HCO_2H in MeCN) were used in a linear gradient (5% B to 100% B) in 2.4 min and then held for 0.1 min at 100% B (total run time: 2.6 min). The LC system was coupled to a SQD mass spectrometer. Analytical LC-HRMS (ESI) analysis was performed on a Waters Alliance 2695 system. Samples were injected directly and the LC system was coupled to a Waters LCT Premier XE Micromass equipped with a Lock Mass probe operating in positive electrospray mode. Eluents A (0.1% HCO_2H in H_2O) and B (0.1% HCO_2H in MeCN) were used in a 1:1 ratio for a total run time of 2 min.

Suppl. Fig. 1 depicts the general synthetic scheme used in this work for the synthesis of (*Z*)-2-((8,10-dimethyl-2,3,6,7-tetrahydro-1*H*,5*H*-pyrido[3,2,1-*ij*]quinolin-9-yl)methylene)benzo[*b*]thiophen-3(2*H*)-one (referred to as HTI-J in the main article).

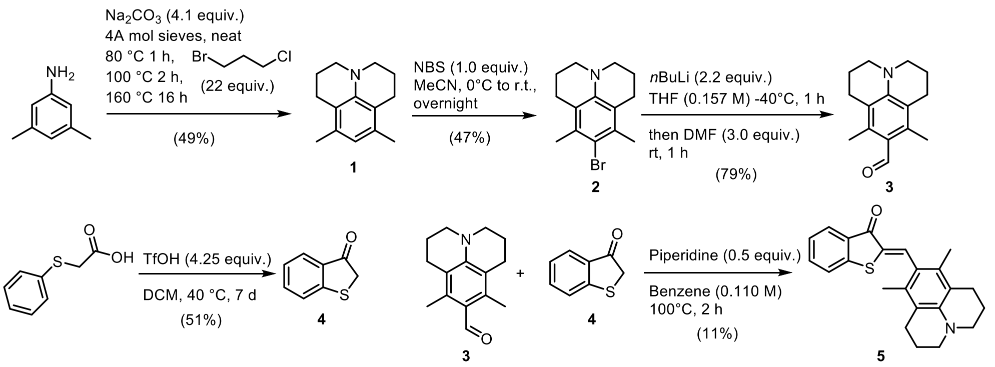

Suppl. Fig. 1 Synthetic scheme for the synthesis of (*Z*)-2-((8,10-dimethyl-2,3,6,7-tetrahydro-1*H*,5*H*-pyrido[3,2,1-*ij*]quinolin-9-yl)methylene)benzo[*b*]thiophen-3(2*H*)-one (**5**, referred to as HTI-J in the main article). Each step was adapted for larger scale synthesis from literature reports as indicated and repeated multiple times. The yields of one specific experiment are shown. NBS = N-bromosuccinimide; nBuLi = n-butyllithium; TfOH = trifluoromethanesulfonic acid (triflic acid); MeCN = acetonitrile; THF = tetrahydrofuran; DMF = N,N-dimethylformamide; DCM = dichloromethane.

Building block syntheses

8,10-dimethyl-2,3,6,7-tetrahydro-1*H*,5*H*-pyrido[3,2,1-*ij*]quinoline (3,5-dimethyljulolidine) (**1**) [1, 2]

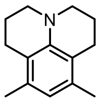

3,5-Dimethylaniline (14.4 g, 116.10 mmol, 1.0 equiv.), 1-bromo-3-chloropropane (397.4 g, 2.52 mol, 21.75 equiv.), sodium carbonate (57.7 g, 482.8 mmol 4.2 equiv., oven dried at 300 °C for 4 hours) and 4 Å molecular sieves (12.25 g activated at 300 °C over night) were added to a flame dried 1 L round bottom flask under a nitrogen atmosphere. The following heating rate was applied: 1 hr. at 80 °C, 2 hr. at 100 °C, 12 hr. at 160 °C. The reaction mixture was cooled to room temperature, diluted with DCM, then washed successively with: 10% HCl, twice with water, once with 10% NaOH and twice with water. The organic fraction was dried over $NaSO_4$, evaporated to dryness and then purified by flash column chromatography on silica gel (hexanes/DCM 60% to 100%), which afforded the title compound **1** as a white crystalline powder (15.7 g, 49% yield). Characterization data matched the previously

reported data: **^{1}H NMR** (400 MHz, $CDCl_3$): δ 6.39 (s, 1H), 3.11 – 3.02 (m, 4H), 2.64 (t, J = 6.8 Hz, 4H), 2.13 (s, 6H), 2.08 – 1.97 (m, 4H) ppm. **^{13}C NMR** (101 MHz, $CDCl_3$): δ 143.8, 133.9, 120.5, 118.5, 50.3, 25.0, 22.6, 19.7 ppm. **LCMS (ESI):** m/z $[M + H]^+$ calcd for $C_{14}H_{20}N^+$ 202.2; found 202.0.

9-bromo-8,10-dimethyl-2,3,6,7-tetrahydro-1*H*,5*H*-pyrido[3,2,1-*ij*]quinoline (**2**) [1]

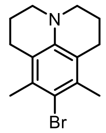

A solution of N-bromosuccinimide (11.67 g, 65.57 mmol, 1.0 equiv.) in MeCN (160 mL) was added dropwise into a solution of 3,5-dimethyljulolidine **1** (13.20 g, 65.57 mmol, 1 equiv.) in MeCN (160 mL) at 0 °C. The resultant suspension was stirred vigorously overnight. After removing all the volatiles under reduced pressure, the residual solid was dissolved in excess amount of DCM and washed with brine. The organic phase was collected, dried with Na_2SO_4, evaporated to dryness and purified by flash chromatography on silica (hexanes/DCM 25%) provided the title compound as white crystalline powder (8.6 g, yield 47%). Characterization data matched the previously reported data: **^{1}H NMR** (400 MHz, $CDCl_3$): δ 3.07 – 3.01 (m, 4H), 2.70 (t, J = 6.8 Hz, 4H), 2.32 (s, 6H), 2.07 – 1.95 (m, 4H) ppm. **^{13}C NMR** (101 MHz, $CDCl_3$): δ 143.2, 133.6, 120.1, 116.9, 50.1, 26.7, 22.7, 20.3 ppm. **LCMS (ESI):** m/z $[M + H]^+$ calcd for $C_{14}H_{19}BrN^+$ 282.1; found 281.8.

8,10-dimethyl-2,3,6,7-tetrahydro-1*H*,5*H*-pyrido[3,2,1-*ij*]quinoline-9-carbaldehyde (3,5-dimethyl-4-formyljulolidine) (**3**) [3]

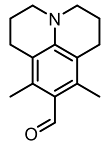

3,5-dimethyl-4-formyljulolidine **3** was accessed via a hitherto unpublished procedure provided by Dube and coworkers (*personal communication*): Under an argon atmosphere, bromide **2** (7.4 g, 26.4 mmol, 1.0 equiv.) was dissolved in anhydrous THF (140 mL) and cooled to −40 °C. *n*-BuLi (2.3 M in hexanes , 25.3 mL, 58.1 mmol, 2.20 equiv.) was added dropwise via a cannula and the solution was stirred at −40 °C for 1 h, then anhydrous DMF (0.165 mL, 2.14 mmol, 1.20 equiv.) was added. The mixture was stirred at −40 °C for 15 min, then the temperature was allowed to raise to ambient temperature at which the mixture was further stirred for 1 hr. A saturated aqueous solution of ammonium chloride (300 mL) was added and the aqueous phase was extracted with DCM (3 × 150 mL). The combined organic phases were dried over

Na_2SO_4, evaporated to dryness and purified by flash column chromatography on silica (hexane/EtOAc 5% to 10%), which afforded title compound **3** as an off white solid (4.77 g, 79% yield). Characterization data matched the previously reported data: **^{1}H NMR** (400 MHz, $CDCl_3$): δ 10.43 (s, 1H), 3.24 – 3.17 (m, 4H), 2.67 (t, J = 6.6 Hz, 4H), 2.43 (s, 6H), 2.03 – 1.92 (m, 4H) ppm. **LCMS (ESI):** m/z $[M + H]^+$ calcd for $C_{15}H_{20}NO^+$ 230.2; found 230.3.

Benzo[*b*]thiophen-3(2*H*)-one (**4**) [4]

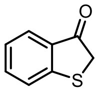

To an oven-dried 500 mL flask equipped with a magnetic stir bar and a condenser were added thiophenoxy acetic acid (17.5 g, 100 mmol, 1.0 equiv.) and DCM (175 mL, 0.57 M). The solution was purged with nitrogen for 5 min. TfOH (37.5 mL, 424.5 mmol, 4.25 eq.) was carefully added in four portions at the 0 h, 14 h, 64 h and 85 h time points. After the first TfOH addition, the reaction flask was purged with argon and then place into a prewarmed oil bath at 40 °C. The reaction mixture was continuously stirred at 40 °C for 7 days in total, after which no SM was observed via TLC. The reaction mixture was cooled to room temperature and carefully poured into a beaker with ice (˜1500 mL). Ethyl acetate (1500 mL) was added. The organic layer was separated and the aqueous layer was further extracted with Ethyl acetate (1000 mL). The combined organic layers were washed with sat. aq. $NaHCO_3$ (1000 mL) and brine (500 mL), dried over anhydrous Na_2SO_4, filtered and concentrated under reduced pressure by rotary evaporation to provide the title compound **4** as a yellow/orange solid crude product (8.5 g, 90% purity, 52% yield), which was used in the next step without further purification.

HTI-J synthesis

(*Z*)-2-((8,10-dimethyl-2,3,6,7-tetrahydro-1*H*,5*H*-pyrido[3,2,1-*ij*] quinolin-9-yl)methylene)benzo[*b*]thiophen-3(2*H*)-one (**5**) [3]

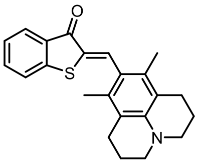

Benzo[*b*]thiophen-3(2*H*)-one **4** (2.5 g, 14.15 mmol, 1.0 equiv., 90% pure), benzene (130 mL, bubbled with argon), 3,5-dimethyl-4-formyljulolidine **3** (3.24 g, 14.15 mmol), and piperidine (0.8 mL, 0.5 equiv.) were added to a dry round bottom flask (500 mL). The reaction mixture was stirred for 2 h at 100 °C. After cooling to room temperature, a saturated NH_4Cl solution (150 mL) was added. The aqueous phase was extracted

with EtOAc (3 x 200 mL). The organic phases were combined, dried over Na_2SO_4 and evaporated to dryness. The crude product was purified by column chromatography on silica. The solid obtained by concentration had to be repurified by washing with pure hexanes to afford title compound **5** (709.0 mg, 12%) as dark red solid. Characterization data matched the previously reported data: **^{1}H NMR** (400 MHz, $CDCl_3$): δ 8.14 (s, 1H), 7.92 (dt, J = 7.8, 1.0 Hz, 1H), 7.52 (ddd, J = 8.3, 7.2, 1.4 Hz, 1H), 7.38 (dt, J = 8.0, 0.9 Hz, 1H), 7.29 – 7.21 (m, 1H), 3.16 – 3.08 (m, 4H), 2.66 (t, J = 6.7 Hz, 4H), 2.10 (s, 6H), 2.02 (h, J = 6.4 Hz, 4H) ppm. **^{13}C NMR** (101 MHz, $CDCl_3$): δ 187.7, 147.1, 145.6, 137.0, 135.1, 132.3, 131.8, 127.1, 125.1, 124.0, 123.4, 118.7, 50.2, 25.4, 22.3, 17.3 ppm. **LCMS (ESI):** m/z $[M + H]^+$ calcd for $C_{23}H_{24}NOS^+$ 362.2; found 362.1.

Suppl. Figs. 2 and 3 show the measured NMR and LCMS spectra of the synthesized HTI-J compound.

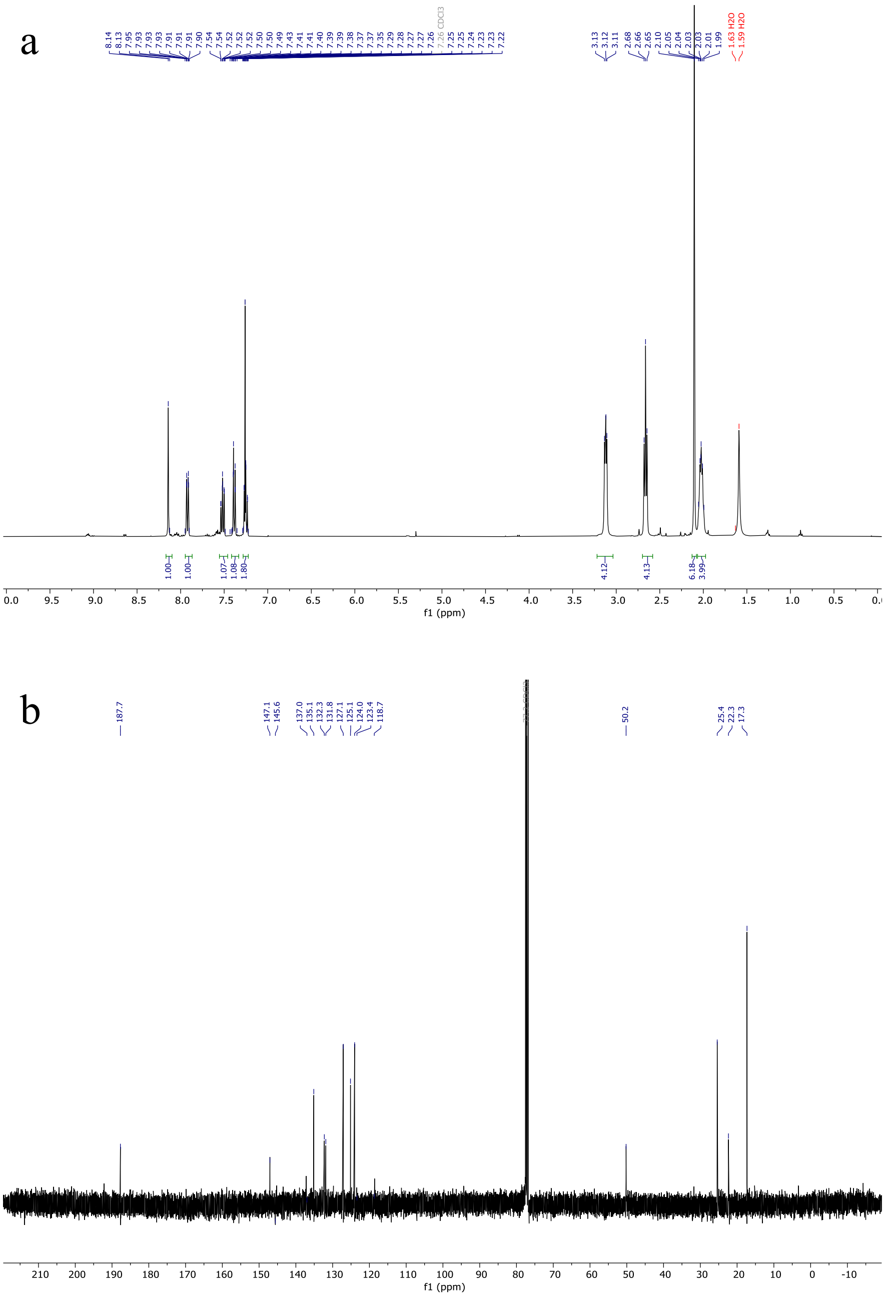

Suppl. Fig. 2 ^{1}H (**a**) and ^{13}C (**b**) NMR spectra of HTI-J (Z-isomer).

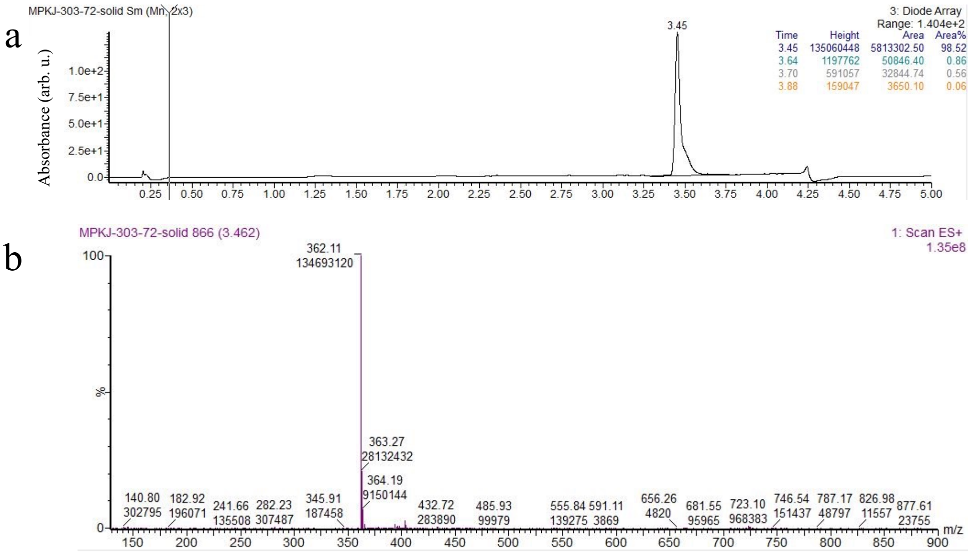

Suppl. Fig. 3 LCMS spectra of HTI-J (Z-isomer). **a** High-pressure liquid chromatography (HPLC) chromatogram of HTI-J. **b** Electrospray ionization (ESI) mass spectrum of HTI-J.

2 Suppl. Note 2. Stationary absorption spectrum of HTI-J in acetonitrile

Suppl. Fig. 4 shows the normalized stationary absorption spectrum of HTI-J in acetonitrile (Z-isomer). The spectrum was recorded on a NanoDrop spectrophotometer using a drop from the 3 mM sample solution measured during the scattering experiments at SwissFEL. The spectrum is characterized by a broad absorption band from around 400 to 550 nm with a peak around 470 nm. During the scattering experiments, the sample solution was excited near the maximum at 470 nm. Optical transient absorption spectra were recorded with an excitation wavelength of 490 nm.

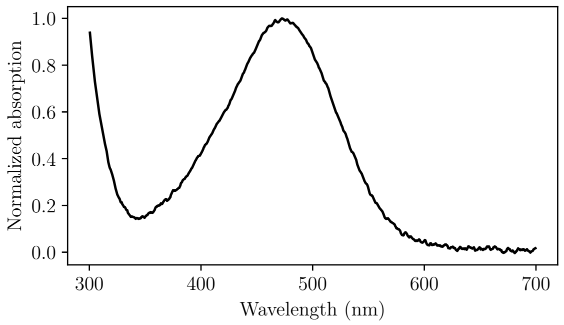

Suppl. Fig. 4 Normalized stationary absorption spectrum of HTI-J in acetonitrile (Z-isomer). During the scattering measurements, the sample solution was excited near the maximum at a wavelength of 470 nm. Optical transient absorption spectra were recorded with an excitation wavelength of 490 nm.

3 Suppl. Note 3. Optical transient absorption spectroscopy

The data handling and analysis of the transient absorption spectroscopy (TAS) data was performed within the KimoPack Python package (version 7.12.14), details on which have been published by Müller et al. [5]. Suppl. Fig. 5a shows the average of 19 difference absorption spectra, ΔA^{raw}, recorded from a sample solution of HTI-J in acetonitrile with a concentration of 0.3 mM. Transient absorption data were recorded with time delays up to 1 ns, but no signal was observed at time delays longer than the first ~10 ps and the analysis was therefore constrained to a time range from -5 to 30 ps as shown in the displayed spectrum. The difference absorption spectrum before time zero is expected to be close to zero and to contain only random noise. A non-zero difference absorption for negative time delays, as can be clearly seen in 5a, indicates artifact contributions. Artifacts and background signals were removed by subtracting the average before time zero, $< \Delta A^{\mathrm{raw}} >_{\Delta t<0}$, for each probe wavelength, resulting in the spectrum shown in Suppl. Fig. 5b. Still, the data recorded at a probe wavelength below 530 nm appear noisy and are affected by the pump scatter around the excitation

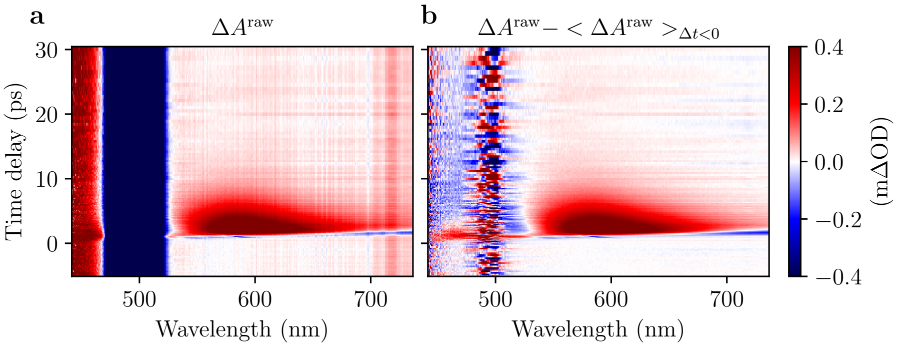

Suppl. Fig. 5 **a** Difference absorption data, ΔA^{raw}, for HTI-J in acetonitrile excited at a laser wavelength of 490 nm and probed in the visible range from around 450 to 750 nm. The data recorded for wavelengths below around 530 nm are dominated by artifacts as can be seen by the non-zero difference absorption for negative time delays. **b** The mean before time zero, $< \Delta A^{\mathrm{raw}} >_{\Delta t<0}$, is subtracted to remove background contributions, but the data recorded for wavelengths below 530 nm still appear noisy and affected by the pump scatter around 490 nm and are therefore not considered in the data analysis. Due to pump scatter within the range of the absorption spectrum of HTI-J (see Suppl. Fig. 4), the ground state bleach is not observed, and vibrational cooling in the ground state could not be inferred from the TAS data.

wavelength of 490 nm and were therefore masked prior to data analysis. The spectrum was further corrected for chirp and time zero and binned by a factor of two along the wavelength axis. The resulting transient absorption spectrum as a function of the probe wavelength and the time delay is shown in Suppl. Fig. 6a.

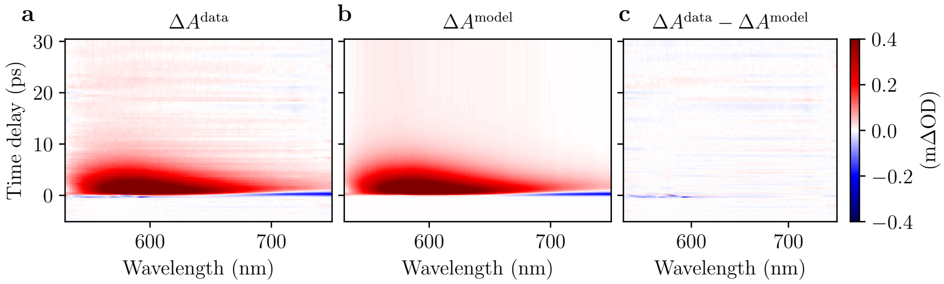

Suppl. Fig. 6 **a** Background subtracted, masked and chirp corrected difference absorption data, ΔA^{data}, for HTI-J in acetonitrile. Following photoexcitation at a laser wavelength of 490 nm, a broad ESA band appears from around 540 to 690 nm that decays within ∼10 ps as well as a short-lived SE band that can be seen from around 670 to 750 nm. **b** Modeled difference absorption spectrum, $\Delta A^{\mathrm{model}}$, resulting from global fitting with a kinetic model consisting of a sum of three exponential decay components and an offset component. **c** The model describes the data well as can be seen by the small residual. Some artifacts remain around time zero, in particular for wavelengths below 600 nm, that might arise from cross phase modulation [6, 7].

The spectrum shows a broad excited state absorption (ESA) band from around 540 to 690 nm that decays within ∼10 ps and a short-lived stimulated emission (SE) band that can be seen from around 670 to 750 nm, the red edge of the probed range. The transient absorption data were globally fitted with a kinetic model consisting of a sum of three exponential decay components convolved with the instrument response function (IRF) and a constant offset. Suppl. Figs. 6b and c show the modeled spectrum and the residual, the difference between the data and the model. The model describes the data well as can be seen by the small residual (R-squared = 0.996). Some short-lived artifacts remain around time zero, in particular for wavelengths below 600 nm, which might arise from cross phase modulation [6, 7]. The best fit parameters, including uncertainties based on a 68% confidence interval, are compiled in Suppl. Table 1.

Suppl. Table 1 Characteristic times (in ps) resulting from the global modeling of the optical TAS data and the initial input values. Values in parenthesis indicate the confidence interval (68%).

Parameter	Value	Initial value
τ_1^{TAS}	0.5 (-0.1/+0.2)	0.5
τ_2^{TAS}	1.2 (-0.5/+0.8)	1.0
τ_3^{TAS}	2.6 (-0.6/+0.4)	2.5
t_0	-0.12 (-0.03/+0.00)	0.00
resolution	0.32 (-0.03/+0.04)	0.11

Lifetimes are interpreted as

τ_1: Decay of stimulated emission and structural and vibrational relaxation of the solute in the excited state.

τ_2: Structural and vibrational relaxation of the solute in the excited state.

τ_3: Internal conversion from the excited to the ground state (excited state lifetime).

Suppl. Figs. 7a and b show the concentration profiles and the decay-associated spectra (DAS) resulting from global fitting of the TAS data. In accordance with observations by Wiedbrauk et al. [3], the 0.5 ps and 1.2 ps components are attributed to decay of stimulated emission and structural and vibrational relaxation of the solute in the excited state, while the 2.6 ps component is interpreted as the lifetime of the excited state. The offset component likely accounts for a minor population of the *E* isomer through photoisomerization.

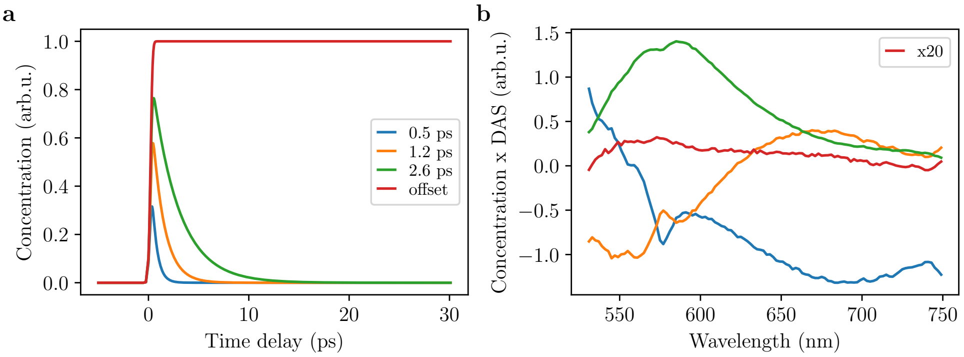

Suppl. Fig. 7 **a** Concentration profiles and **b** corresponding decay associated spectra (DAS) resulting from the global fitting of the femtosecond optical TAS data of HTI-J in acetonitrile. The spectra are attributed to stimulated emission and structural and vibrational relaxation of the solute in the excited state (0.5 ps and 1.2 ps), and the decay of the excited state (2.6 ps). The offset component likely accounts for a minor population of the *E* isomer through photoisomerization. The offset component has been scaled by a factor of 20 to enhance the visibility of the spectral features.

4 Suppl. Note 4. Time-resolved X-ray solution scattering

Time-resolved X-ray solution scattering (TR-XSS) measurements were carried out in 2022 at the Alvra endstation of the SwissFEL X-ray free electron laser under proposal number 20220249. Suppl. Table 2 summarizes key setup parameters of the TR-XSS measurements. HTI-J in acetonitrile was measured in two time ranges, -1.5 to 4.5 ps

Suppl. Table 2 Key experimental parameters of the TR-XSS measurements.

Laser	Wavelength	470 nm
	Pulse energy	12 µJ
	Pulse duration	50 fs (FWHM)
	Spot size	73x95 µm (FWHM)
X-ray	Energy	11 keV
	Pulse duration	30 fs (FWHM)
	Spot size	30x30 µm (FWHM)
Sample	HTI-J in acetonitrile	
	Concentration	3 mM
Delivery	Cylindrical jet	100 µm
Other	Detector	Jungfrau 16M
	Sample-detector distance	9.7 cm
	Repetition rate	100 Hz
	Pulse scheme	6 laser-on, 1 laser-off
	Timing tool	Yes
	Time resolution	∼120 fs

in 0.1 ps steps (short time scans, 21 scans recorded), and -10 to 60 ps in 1 ps steps (long time scans, 18 scans recorded). Each time step in a scan contains 1000 scattering images, resulting in 61000 images for each short-time scan and 71000 images for each long-time scan. The following suppl. notes contain further details on the experiment and the data reduction and processing required prior to data analysis.

4.1 Suppl. Note 4.1. Laser fluence scan

Laser fluence scans, also referred to as power titration, were performed during the experiment. The scattering response of HTI-J in acetonitrile was recorded for a laser energy ranging between 0.7 and 26 µJ with a fixed time delay of 20 ps. Suppl. Fig. 8 shows the sum of the absolute of the azimuthally integrated difference scattering signal in a q-range between 1 and 3 Å^{-1}, which has been selected to reflect the response of the signal, as a function of the pump laser energy. Apart from the data at the lowest laser

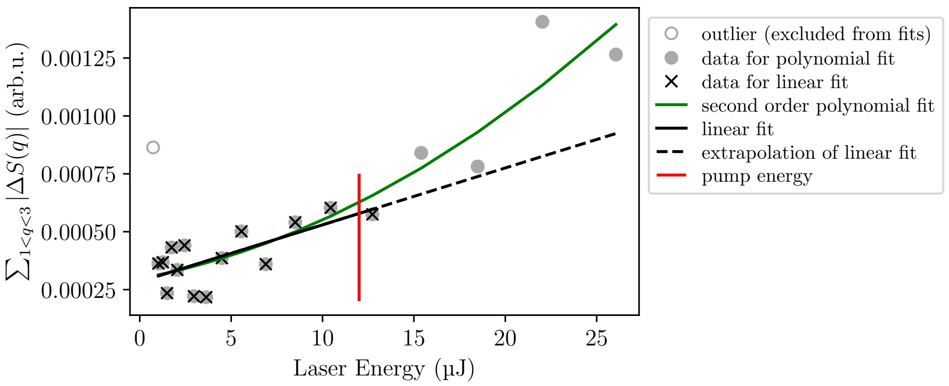

Suppl. Fig. 8 Response of the scattering signal, defined here as the sum of the absolute of the difference scattering signal in a q-range between 1 and 3 Å^{-1}, as a function of the pump laser energy. The data at the lowest laser energy of 0.76 µJ appears to be an outlier. Apart from this data point, the sum of the absolute of the difference scattering shows an upward trend with increasing laser energy. Data (filled markers) were modeled using a second order polynomial function and data between 1 and 13 µJ (x markers) were modeled using a linear function and extrapolated to higher energies. The second order polynomial begins to deviate from the linear fit around a laser energy of 10–13 µJ, and the signal response becomes nonlinear for higher energy. The scattering data discussed in this study were recorded with a laser excitation energy of 12 µJ.

energy of 0.76 µJ, which appears to be an outlier, the intensity of the scattering signal shows an overall increase with higher laser energies. Data have been modeled with a second order polynomial fit and data between 1 and 13 µJ have also been modeled using a linear function. The linear fit was extrapolated to higher laser energy. The fits indicate that around a laser energy of 10–13 µJ, the second-order polynomial begins to deviate from the linear fit, suggesting that the signal response becomes nonlinear at higher laser energies. For the HTI-J in acetonitrile experiments, a pump laser energy

of 12 µJ per pulse—at the upper end of the linear-response region and corresponding to a fluence of around 1.2 mJ/mm^{-2}—was chosen to maximize signal-to-noise ratio. A discussion of the influence of the laser energy on the observed dynamics is provided in Suppl. Note 8.

4.2 Suppl. Note 4.2. Reduction of TR-XSS data

Following data acquisition, the detector pedestal, a background detector image acquired without exposure to radiation, was subtracted from each raw scattering image. The resulting image was then multiplied by the gain factors of the detector and re-binned (2x2 pixels) to reduce the size of the stored images. The loss of resolution through re-binning of the 16 Mpixel Jungfrau detector is acceptable for the recorded X-ray solution scattering images as the signals of interest display broad oscillating features. The stored 2D scattering images were corrected for the geometry of the experimental setup, i.e. the solid angle coverage of the detector, X-ray polarization [8], and absorption in the active layer of the detector. Additionally, defects, module borders, and shadows on the detector were masked out. Suppl. Figs. 9a-d show the corrections and Suppl. Fig. 9e shows an example of a corrected and masked detector image.

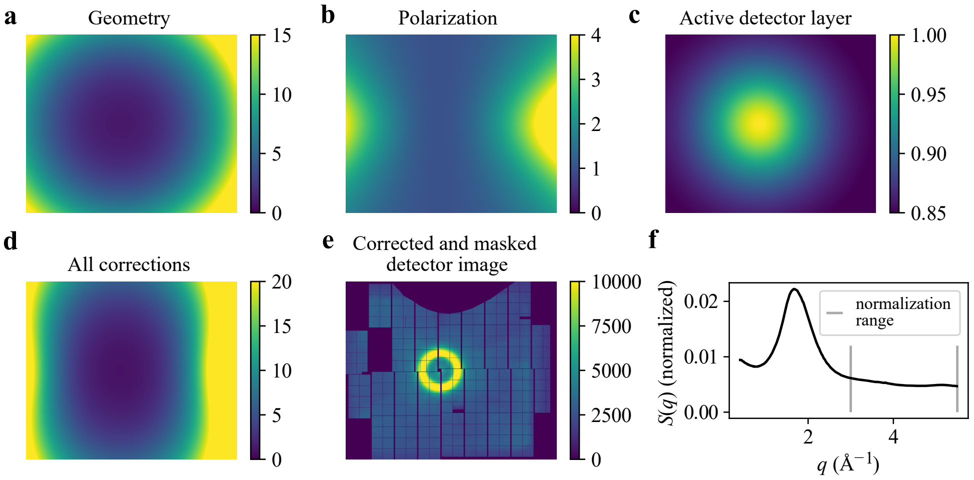

Suppl. Fig. 9 **a-c** Detector corrections applied to the recorded 2D scattering images. **d** Product of the detector corrections shown in **a-c**. **e** Corrected and masked example detector image for HTI-J in acetonitrile. **f** Azimuthal integration of the example image yields the scattering curve, $S(q)$. The signal is normalized by dividing $S(q)$ by the sum of the absolute scattering intensity in the range $3 < q < 5.5$ Å^{-1}. The normalization range is indicated by vertical gray lines.

The stochastic nature of the self-amplified spontaneous emission (SASE) process at XFELs induces substantial shot-to-shot fluctuations in beam intensity, photon energy, and pointing. Combined with sample delivery instabilities and variations in experimental chamber conditions, these effects may alter the spatial distribution of

scattered X-rays on the detector. Although mitigation strategies exist [9, 10], XFEL data reduction typically begins by discarding frames most affected. In the present work, images lacking beamline diagnostics (e.g., intensity monitors or timing tool data) or deviating by more than 10% from the median total scattering intensity within a scan were rejected (Suppl. Fig. 10a). To compensate for beam–jet overlap variations, we applied a correlation filter between summed scattering intensity and the I0 diode signal, removing shots in the lowest and highest deciles (Suppl. Fig. 10b). The filtering resulted

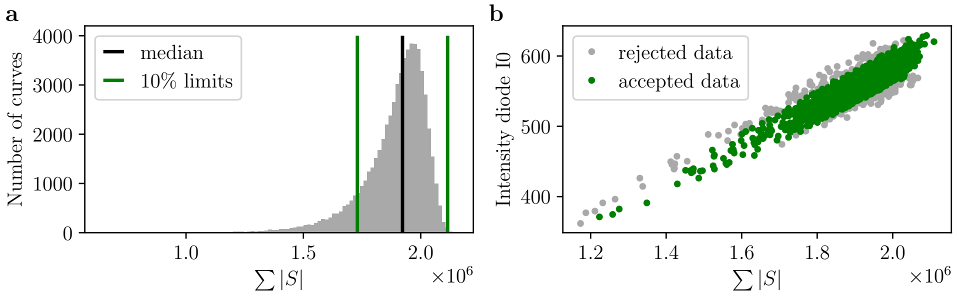

Suppl. Fig. 10 Detector images deviating from the median measurement condition were rejected based on **a** an intensity filter and **b** the correlation between an intensity diode and the summed absolute scattering signal. **a** Histogram of the sum of the absolute scattering signal in an example short time scan (total of 61000 measurements). Measurements deviating more than 10% from the median are rejected. **b** Intensity measured by the I0 intensity diode as a function of the summed absolute scattering signal for an arbitrary example time step (total of 1000 data points). The lowest and highest 10% of the signals deviating from a linear regression fit were rejected.

in a rejection of approximately 30% of the images. A more relaxed outlier filtering was also tested. Rejecting approximately 20% of the images was tested during data reduction, but significantly increased the magnitude of background artifact components in the calculated difference scattering signal. Background artifact components in the TR-XSS data are discussed in Suppl. Note 4.6.

The remaining images were integrated in 500 radial bins and 13 azimuthal bins to yield scattering signals, $S(\Delta t, q, \phi)$ [8], depending on the time delay between pump and probe pulses, Δt, the magnitude of the scattering vector, $q = (4\pi/\lambda)\sin(2\Theta/2)$, where λ is the X-ray wavelength and 2Θ is the scattering angle, and the azimuthal angle ϕ. Each scattering signal was normalized by the summed absolute intensity of itself in a q-range of 3 to 5.5 Å^{-1}: $S_{\text{norm}}(\Delta t, q, \phi) = S(\Delta t, q, \phi)/\sum_{3<q<5.5} \mid S(\Delta t, q, \phi) \mid$. Suppl. Fig. 9f shows an example of a normalized scattering signal. The normalization range is indicated by gray vertical lines. After normalization, the difference scattering signal $\Delta S(\Delta t, q, \phi) = S_{\text{on}}(\Delta t, q, \phi) - S_{\text{off}}(q, \phi)$ was calculated by subtracting a weighted average of the three nearest neighbors without laser excitation (laser-off), where the signals were weighted by the time elapsed between the laser-off measurements to the respective laser-on measurement. The difference scattering signals of both the short- and long-time scans were re-binned into 151 equistatistical time bins based on the timing tool [11] information, averaged (approximately 10000 signals per time

delay), and decomposed into isotropic and anisotropic signal contributions via Legendre decomposition [12, 13]. Suppl. Fig. 11a shows the isotropic, $\Delta S_0(\Delta t, q)$, and Suppl. Fig. 11b the anisotropic, $\Delta S_2(\Delta t, q)$, difference scattering signal. This study focuses

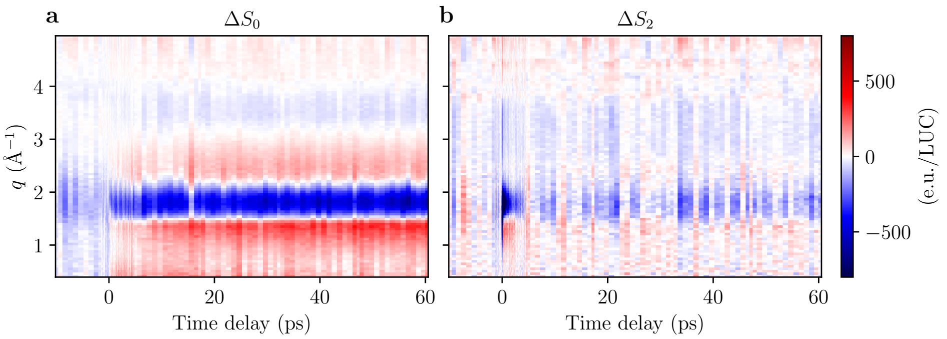

Suppl. Fig. 11 **a** Isotropic, ΔS_0, and **b** anisotropic, ΔS_2, difference scattering signal as a function of the time delay between laser pump and X-ray probe pulses and the magnitude of the scattering vector, q. Early time delays (-1.5 to 4.5 ps) were sampled in 100 fs steps and longer time delays (-10 to 60 ps) in 1 ps steps. **a** The isotropic difference scattering signal is dominated by heating of the bulk solvent, but contains also signal contributions reflecting solvation dynamics of HTI-J in acetonitrile as discussed in the main article. **b** The anisotropic signal is dominated by the response to the optical Kerr effect (OKE), the photo-alignment of molecules by the electric field of the excitation laser [14]. The scattering signals are displayed in electron units per liquid unit cell (e.u./LUC); the scaling to this unit is described in Suppl. Note 4.5.

on the information contained in the isotropic difference scattering signal, which is referred to simply as ΔS hereafter and in the main article. The anisotropic signal is discussed in Suppl. Note 4.3 and the fast signal response to the optical Kerr effect in the anisotropic signal is used to correct time zero and determine the time resolution as described in Suppl. Note 4.4. When the anisotropic difference scattering signal is discussed, it is specified as ΔS_2. To obtain a legible time delay axis, the time delays were shifted by 1048399.9 fs to correct for time zero with respect to the times recorded at the experiment and converted to picoseconds. Along the q-axis, discontinuities in the scattering signal caused by malfunctioning detector pixels that were not accounted for by the initial mask, were identified by visual inspection and subsequently masked. From the initial 500 radial bins, 387 bins remained which were further re-binned into 96 bins to increase the signal-to-noise ratio [15]. The resulting q-axis spans a range from 0.4 to 5 Å^{-1}.

4.3 Suppl. Note 4.3. Singular value decomposition of the anisotropic difference scattering signal

Suppl. Fig. 12a shows the anisotropic difference scattering signal, ΔS_2, in a time delay range from -2 to 15 ps. To investigate the signal components contributing to the anisotropic difference scattering signal, singular value decomposition (SVD) of

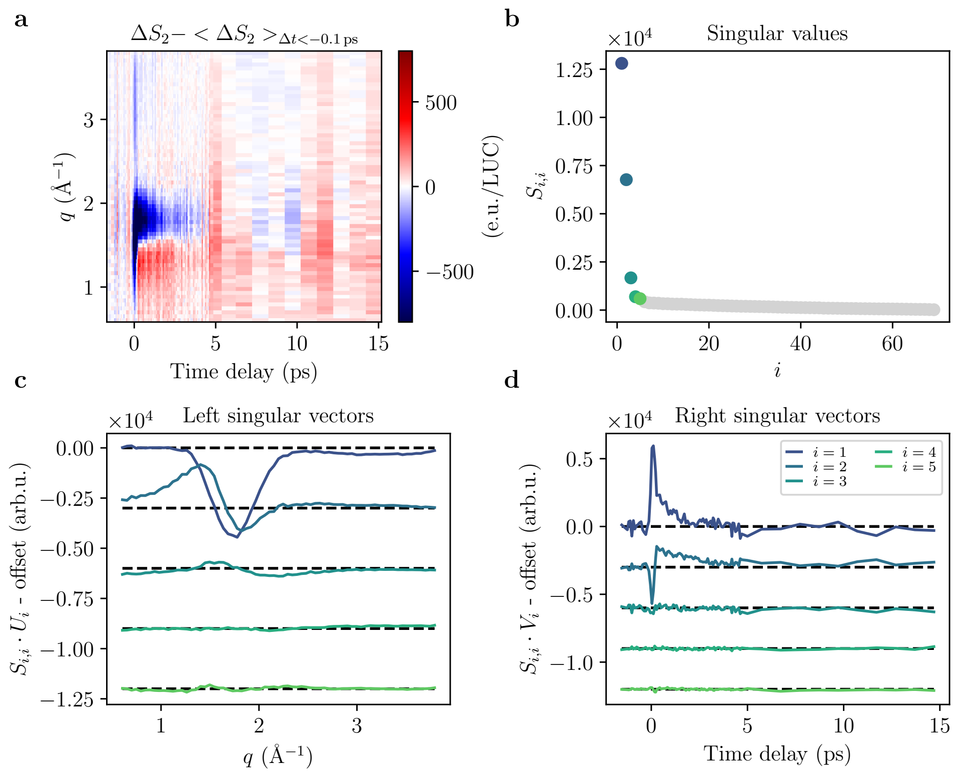

Suppl. Fig. 12 **a** Time-resolved anisotropic difference scattering data, ΔS_2 in a time range from -2 to 15 ps. The signal is dominated by the structural solvent response connected to the optical Kerr effect [14], i.e. the dynamics of re-orientational motion around the molecules photoaligned by the electric field of the excitation laser. **b** Singular values. **c**,**d** Left- and right-singular vectors of the five strongest components. The first two singular vectors arise from the optical Kerr effect and dominate the scattering signal [14].

the displayed scattering data is performed. SVD decomposes an $M \times N$ matrix into orthogonal left- and right-singular vectors $U_{i,i}$, $V_{j,j}$, weighted by singular values $S_{i,j}$, where $i = 1..M$ and $j = 1..N$ [16]. Suppl. Fig. 12b shows the resulting singular values and Suppl. Figs. 12c and d show the left- and right-singular vectors of the first five components. The left-singular vectors (LSVs) contain difference scattering profiles as a function of q and the right-singular vectors (RSVs) the respective temporal information.

The two strongest components are assigned to orientational rearrangement of the solvent associated with the optical Kerr effect [14], which arises form photoalignment of solvent molecules by the electric field of the excitation laser. Anisotropic signal components of similar q-profile and time evolution were reported by Ki et al. [14] and attributed to libration and orientational diffusion motions stemming from the OKE of bulk acetonitrile. Related anisotropic OKE signals have also been reported by Montoya-Castillo et al. for chloroform [17]. Signal components for $i > 2$ are small in

magnitude compared to the signal response to the OKE, showing that the anisotropic signal is dominated by the response to the OKE.

Suppl. Fig. 13 shows the left- and right-singular vectors of components 3 to 5. The decay of the 5th RSV can be fitted with a time constant of 2.5 ±0.8 ps close to

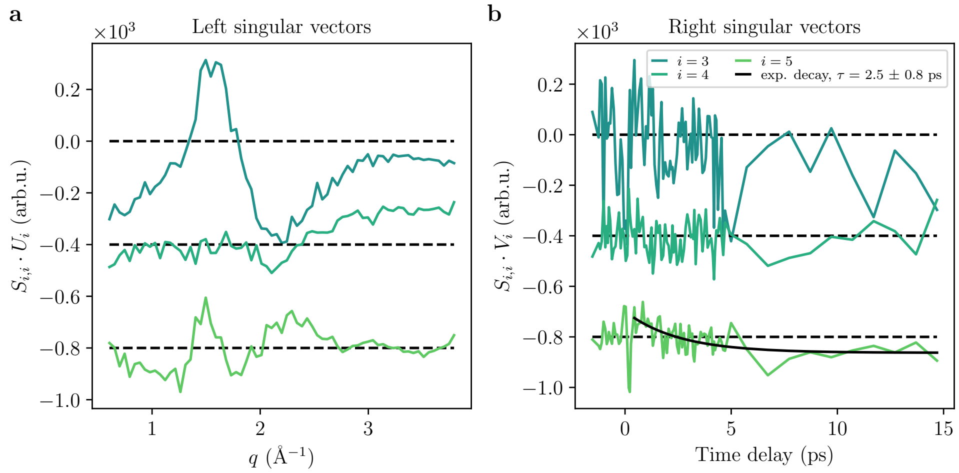

Suppl. Fig. 13 **a** Left-singular vectors and **b** right-singular vectors of SVD components 3 to 5 of the anisotropic difference scattering data. The decay of the 5th RSV can be fitted with a time constant close to the solvation-shell structural reequilibration time from the global fitting of the isotropic difference scattering data (see Suppl. Table 3 and Suppl. Fig. 22), which supports the observation of the solvation-shell dynamics in the scattering data.

the time constant of the solvation-shell structural reequilibration, 3 ± 1 ps, found in the global fitting of the isotropic difference scattering signal (and the excited state lifetime of 2.6 ± 0.5 ps obtained from the TAS data). However, the similarity between this timescale and the slow OKE decay, combined with large artifacts, limits the extent to which solvation-shell contributions can be quantitatively separated from the bulk-solvent OKE in the anisotropic data.

The ultrafast anisotropic signal response to the OKE is used to correct time zero and determine the time resolution as described below.

4.4 Suppl. Note 4.4. Time zero and time resolution

Suppl. Fig. 14a shows the anisotropic difference scattering signal, ΔS_2, in a time delay range from -1.5 to 5 ps, which is dominated by the signal response to the optical Kerr effect (OKE) as discussed in Suppl. Note 4.3 above. In the present study, the response to the OKE in the anisotropic difference scattering signal is used to correct time zero and model the instrument response function (IRF). For this, ΔS_2 was averaged in a q-range from 2.6 to 3.6 Å^{-1}, where ΔS_2 shows a short lived signal response, as shown in Suppl. Fig. 14. The averaged data was multiplied by -1 to invert the signal response

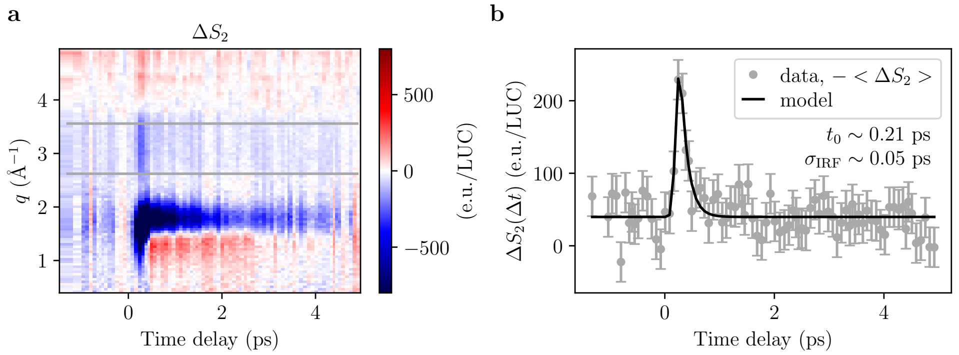

Suppl. Fig. 14 **a** Anisotropic difference scattering signal, ΔS_2, at early time delays. The signal is dominated by the response to the optical Kerr effect (see Suppl. Note 4.3). The horizontal lines at 2.6 and 3.6 Å^{-1} indicate the q-range in which ΔS_2 was averaged to model the temporal response of the signal. **b** The average ΔS_2 ($2.6 < q < 3.6$ Å^{-1}) was multiplied by -1 and modeled by a step-function rise and a single exponential decay convolved with the instrument response function (IRF) and a constant offset (see eq. 13) to account for the non-zero background that can be seen at negative time delays. Error bars correspond to one standard deviation of the data points for $\Delta t < -0.3$ ps (12 data points). The time zero, t_0, and the Gaussian width of the IRF, σ_{IRF}, are extracted form the fit. The difference scattering signals are corrected for t_0 and σ_{IRF} is used to fix this parameter in the global fitting.

and modeled by a step-function rise and a single exponential decay convolved with the IRF and a constant offset (see eq. 13). The offset is included to account for the non-zero background that can be seen for negative time delays. The modeling yields a time zero of around 0.21 ps, which was used to correct the difference scattering data presented in this study, and a Gaussian width of the IRF of $\sigma_{\mathrm{IRF}} \sim 0.05$ ps. Converted to full width at half maximum (FWHM), this corresponds to an IRF of approximately 120 fs. An estimate of the IRF from the experimental parameters given at the Alvra endstation at SwissFEL yields a similar IRF of around 130 fs (FWHM), calculated using IRF $\approx \sqrt{t_{\mathrm{laser}}^2 + t_{\mathrm{X-ray}}^2 + t_{\mathrm{jet}}^2}$, with a pulse width of the optical laser of $t_{\mathrm{laser}} \approx 50$ fs (FWHM), a pulse width of the X-rays of $t_{\mathrm{X-ray}} \approx 30$ fs (FWHM) and a temporal smearing due to the group velocity mismatch for the laser and X-ray pulses $t_{\mathrm{jet}} \approx 120$ fs. t_{jet} was calculated from the group velocity mismatch $(n_{\mathrm{laser}} - n_{\mathrm{X-ray}})/c = 1.2$ fs/µm, where n is the refractive index ($n_{\mathrm{laser}} = 1.344$ [18] for the optical laser in acetonitrile and $n_{\mathrm{X-ray}} \sim 1$) and c is the speed of light. For a jet with a 100 µm diameter, this results in t_{jet} of approximately 120 fs. Time jitter between the pump and probe pulses was corrected using the timing tool [11] and therefore neglected in the estimation of the IRF.

4.5 Suppl. Note 4.5. Liquid unit cell scaling

To allow for quantitative data analysis, the intensity of the measured scattering signal was converted to electron units per liquid unit cell (e.u./LUC). The LUC is the smallest stoichiometrically representative unit and is constructed to include a single solute and

N solvent molecules, where N depends on the concentration of the solution, c, and the density, ρ, and molar mass, M, of the solvent:

$$N = \frac{\rho}{M \cdot c} \tag{1}$$

For the measured solution of HTI-J in acetonitrile (c = 3 mM, ρ = 786 g/L [18], M = 41.05 g/mol), the LUC includes around 6400 acetonitrile molecules.

Suppl. Fig. 15a shows the scattering signals, $S_{\mathrm{acetonitrile}}$ and $S_{\mathrm{HTI-J}}$, arising from scattering of the LUC. The scattering signal of liquid acetonitrile is a reference signal

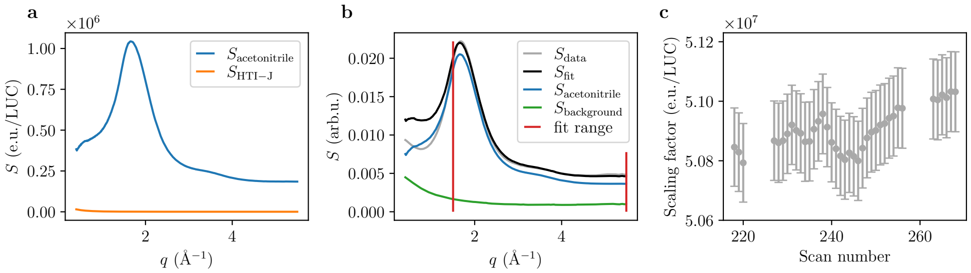

Suppl. Fig. 15 Conversion of the measured scattering intensity to electron units per liquid unit cell (e.u./LUC). **a** Scattering signal of liquid acetonitrile from [19] scaled by the number of solvent molecules per solute in the sample solution (~6400) and the scattering signal arising from coherent and incoherent scattering of a single HTI-J. As $S_{\mathrm{HTI-J}}$ is small compared to $S_{\mathrm{acetonitrile}}$, it was neglected when fitting the experimental data. **b** Fitting of the measured data (median of laser-off curves, normalized), S_{data}, with $S_{\mathrm{acetonitrile}}$ and a background component, $S_{\mathrm{background}}$, returns the fitted curve, S_{fit}. The fit was constrained to $q > 1.5$ Å^{-1} as the data and reference differ at low q, which impacted the quality of the fit. **c** Scaling factor and associated uncertainty (error bars obtained by propagating the standard deviation of the best-fit parameters) for scaling of the measured data to e.u./LUC as a function of the scan number.

in e.u. per molecule taken from work by Kjær et al. [19] and was multiplied by N, while $S_{\mathrm{HTI-J}}$ is the sum of the coherent and incoherent (Compton) scattering of one HTI-J molecule. The coherent scattering was calculated via the Debye equation [20] from a ground state structure of HTI-J generated with density functional theory (DFT). $S_{\mathrm{HTI-J}}$ is small compared to $S_{\mathrm{acetonitrile}}$ and was therefore neglected when modeling the experimental data.

The scaling factor to convert the experimental data to units of e.u./LUC was determined for each scan separately by fitting the median of the normalized laser-off images in each scan with a linear combination of the scattering of N acetonitrile molecules and a background component:

$$S_{\mathrm{fit}}(q) = a \cdot S_{\mathrm{acetonitrile}}(q) + b \cdot S_{\mathrm{background}}(q). \tag{2}$$

The background scattering component was obtained from the median of azimuthally integrated detector images recorded without the sample solution in the path of the

X-ray beam and is comprised of scattering of the helium gas that filled the sample chamber and stray scattering from upstream beamline components. Suppl. Fig. 15b shows the fitting of the experimental scattering data, S_{data}, for an example scan (scan number 240). The fit range of $q > 1.5$ Å^{-1} was chosen as there is a discrepancy between the acetonitrile reference and the measured scattering data at lower q that affected the quality of the fit. In the fitted range, the data and fit agree well, but at low q, the fit exhibits higher intensity than the data, which could be due to residual background or air scattering in the acetonitrile reference. As part of the background scattering is absorbed in the jet, a measurement without the jet is also not a perfect representation of the background, which can lead to discrepancies in the fitting. Suppl. Fig. 15c shows the scaling factor, $1/a$, as a function of the scan number of the data analyzed in this study. The scattering signals in each scan were scaled by the respective scaling factor.

4.6 Suppl. Note 4.6. Identification of background artifact components

Artifacts in the scattering data were identified through singular value decomposition (SVD) of the difference scattering data at negative time delays [21]. At negative time delays, laser-off images are subtracted from laser-off images and the difference signal should hence be flat and contain only random noise. Suppl. Fig. 16a shows the isotropic difference scattering signal in a time range from -10 to 5 ps. The difference scattering signal before time zero is not flat as can, for example, be seen by the negative intensity around $q = 1.8$ Å^{-1}. The signal variation at negative time delays reveals the presence of artifacts that likely arise from fluctuations in X-ray intensity and energy [9] and are expected to affect the scattering data at all time delays [21]. To identify the most prominent artifact contributions, SVD of the difference scattering data in the time range $-10 < \Delta t < -0.3$ ps (black vertical lines in Suppl. Fig. 16a) was calculated. Suppl. Fig. 16b shows the singular values, while the left- and right-singular vectors of the four strongest components are displayed in Suppl. Figs. 16c and d. The magnitude of the RSVs fluctuates in time, but none of them show a pronounced time-evolution. The LSVs show a noticeable q-dependence, but the magnitude of the components becomes small for $i > 3$. The q-profile of the third LSV appears to be similar to a signal component in the difference scattering data, which carries the risk of removing real signal in a fit including this component [21]. For this reason, only the first two LSVs are included in the data modeling, meaning that the background artifact components, $\Delta S^{\text{bkg}}(\Delta t, q)$, are given by

$$\begin{aligned}\Delta S^{\text{bkg}}(\Delta t, q) &= a_1^{\text{bkg}}(\Delta t) \cdot S_{1,1} U_1(q) + a_2^{\text{bkg}}(\Delta t) \cdot S_{2,2} U_2(q) \\ &= a_1^{\text{bkg}}(\Delta t) \cdot \Delta S_1^{\text{bkg}}(q) + a_2^{\text{bkg}}(\Delta t) \cdot \Delta S_2^{\text{bkg}}(q)\end{aligned} \tag{3}$$

where $a_1^{\text{bkg}}(\Delta t)$ and $a_2^{\text{bkg}}(\Delta t)$ are amplitudes as a function of the time delay. Noise on the LSVs was smoothed using a Savitzky-Golay filter with a second order polynomial and an 11 points interval before including the components in the fitting.

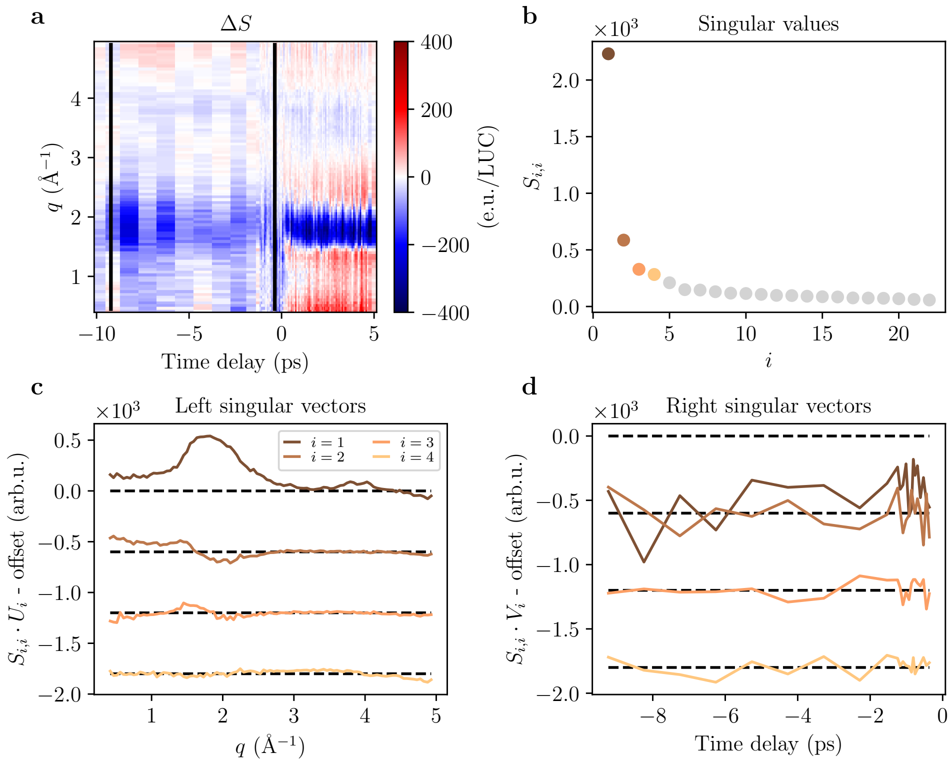

Suppl. Fig. 16 Identification of background artifact contributions to the isotropic difference scattering data, ΔS. Before time zero, ΔS is expected to be flat and contain only random noise. **a** The display of ΔS in a time range from -10 to 5 ps shows that the difference before time zero varies with a noticeable q-dependence. The artifacts are expected to affect the data at all time delays [21]. The most prominent background artifact components are identified through SVD of the data in the time range $-10 < \Delta t < -0.3$ ps (vertical black lines). **b** Singular values. **c**, **d** The left- and right-singular vectors of the four strongest components. The two strongest left-singular vectors were included in the fitting of the scattering data.

4.7 Suppl. Note 4.7. Noise estimation

The noise level on the difference scattering signals was estimated based on a piecewise polynomial method as described by Dent et al. [22]. A third order polynomial was fit to a 16 points interval around each data point in the difference scattering signal. The difference between the data point and the polynomial is assigned as noise, N, and the standard deviation for each data point is calculated as $\sigma = \sqrt{\frac{\sum_i N_i^2}{M}}$, where M is the size of the interval. The estimation is repeated for all data points at all time delays.

5 Suppl. Note 5. Identification of signal components in the TR-XSS data

Signal components contributing to the isotropic difference scattering signal were identified through a stepwise analysis of the experimental data, which involved averaging of the difference signal and singular value decomposition (SVD).

5.1 Suppl. Note 5.1. Bulk solvent heating

Given the lifetime of the excited state of HTI-J in acetonitrile of 2.6 ± 0.5 ps (see Suppl. Note 3), the low photoisomerization yield of HTI-J in polar solvents [3, 23], and vibrational cooling times reported between 10 and 15 ps for acetonitrile [24–26], it is assumed that the majority of the nonequilibrium ensemble of solute molecules returned to the initial ground state and re-established thermal equilibrium at the latest measured time delays, leaving only the change in scattering due to a temperature increase in the solvent [19].

Suppl. Fig. 17 shows the time-resolved isotropic difference scattering data together with an average in two time ranges, between 45-50 ps and between 55-60 ps. Suppl.

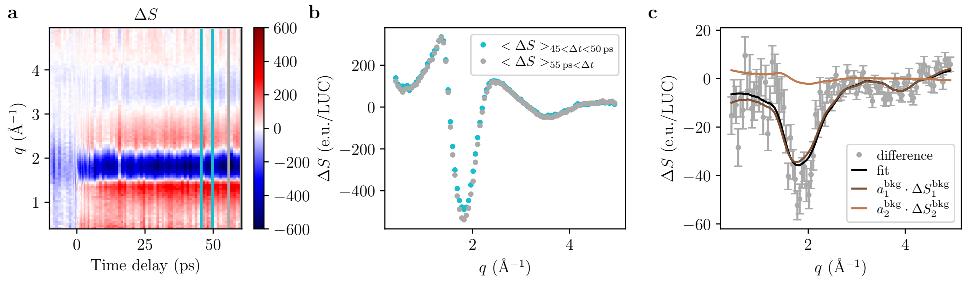

Suppl. Fig. 17 **a** Time-resolved Isotropic difference scattering data. Vertical lines indicate the time ranges of the average curves shown in **b**. **b** Isotropic difference scattering signal averaged between 45-50 ps and 55-60 ps. **c** Difference between the average curves at 45-50 and 55-60 ps (error bars from polynomial noise estimation [22] described in Suppl. Note 4.7). The difference can be explained by background artifact contributions, which supports the assumption that the majority of the nonequilibrium ensemble of solute molecules returned to the initial ground state and re-established thermal equilibrium at the latest measured time delays, leaving only the change in scattering due to a temperature increase in the solvent.

Fig. 17c shows the difference between the two curves. This difference can be explained by contributions from background artifact components (see Suppl. Note 4.6), which supports the assumption that at the latest measured time delays only the changes in scattering due to a temperature increase in the solvent are present.

The difference scattering signal arising from heating of the bulk solvent is therefore determined by averaging the isotropic difference scattering data, ΔS, between 55 and 60 ps as indicated by the gray vertical lines in Suppl. Fig. 17a.

Suppl. Fig. 18a shows the average difference scattering curve, $< \Delta S >_{55\,\mathrm{ps}<\Delta t}$ and the fit with a linear combination of a reference signal for bulk solvent heating for a 1 K heat increase [19] and the two background artifact components (see eq. 3):

$$\Delta S_{\mathrm{fit}}(q) = a^{\mathrm{ref}} \cdot \Delta S^{\mathrm{ref}}_{\Delta\mathrm{T}}(q) + a_1^{\mathrm{bkg}} \cdot \Delta S_1^{\mathrm{bkg}}(q) + a_2^{\mathrm{bkg}} \cdot \Delta S_2^{\mathrm{bkg}}(q). \quad (4)$$

The reference signal, $\Delta S^{\mathrm{ref}}_{\Delta\mathrm{T}}(q)$, was scaled to e.u./LUC/K by multiplying with the

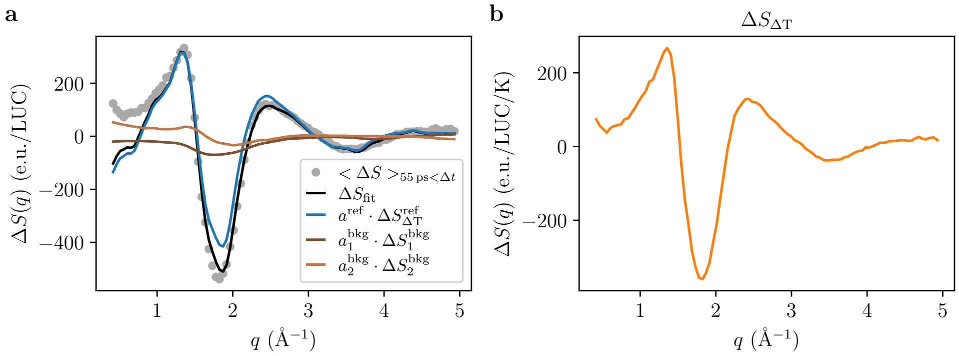

Suppl. Fig. 18 **a** The average isotropic difference scattering data between 55 and 60 ps is fitted with a linear combination of a reference for bulk solvent heating [19], which was multiplied by the number of solvent molecules in the LUC, and two background artifact components. Due to a discrepancy between the measured data and the reference at low q, the fit was constrained to $q > 1.2$ Å^{-1}. **b** $\Delta S_{\Delta\mathrm{T}}$ used for subsequent data analysis was calculated by subtracting the background artifact components from the averaged data and scaling the curve to e.u./LUC/K. The scaling to the reference solvent curve allows to estimate a quantitative time-dependent temperature change, $\Delta T(\Delta t)$, in the solvent in units of Kelvin.

number of solvent molecules in one LUC (see Suppl. Note 4.5). As can be seen in Suppl. Fig. 18a, the data and fitting curve differ at low q, which might be due to unaccounted artifacts in either the measured data or the reference. As a consequence, the fit was constrained to $q > 1.2$ Å^{-1} to optimize the agreement between the data and fit function at the peaks around 1.4 and 2.4 Å^{-1} and at the negative feature around 1.8 Å^{-1}. The fit returned the parameters $a^{\mathrm{ref}} = 1.24 \pm 0.02$, $a_1^{\mathrm{bkg}} = -0.13 \pm 0.01$, and $a_2^{\mathrm{bkg}} = 0.35 \pm 0.08$. The signal attributed to arise from heating of the bulk solvent, $\Delta S_{\Delta\mathrm{T}}$ in e.u./LUC/K, is calculated by subtracting the background artifact contributions and scaling the curve by $1/a^{\mathrm{ref}}$:

$$\Delta S_{\Delta\mathrm{T}}(q) = \frac{1}{a^{\mathrm{ref}}} \left(< \Delta S >_{\Delta t > 55\,\mathrm{ps}} (q) - a_1^{\mathrm{bkg}} \Delta S_1^{\mathrm{bkg}}(q) - a_2^{\mathrm{bkg}} \Delta S_2^{\mathrm{bkg}}(q) \right). \quad (5)$$

Suppl. Fig. 18b shows the resulting $\Delta S_{\Delta\mathrm{T}}$, which was used in the subsequent data analysis, and which allows to estimate a quantitative time-dependent temperature change, $\Delta T(\Delta t)$, in the solvent in units of Kelvin. The shape of $\Delta S_{\Delta\mathrm{T}}$ is fully constrained during the global fitting and not optimized.

5.2 Suppl. Note 5.2. Vibrational relaxation of the solute

Suppl. Fig. 19a shows the background artifact- and heat-subtracted difference signal after fitting the isotropic difference scattering data with the signal response to bulk solvent heating and the two background artifact components:

$$\Delta S_{\mathrm{fit}}(\Delta t, q) = \Delta T(\Delta t) \cdot \Delta S_{\Delta \mathrm{T}}(q) + \Delta S^{\mathrm{bkg}}(\Delta t, q), \tag{6}$$

with $\Delta T(\Delta t)$ described by the kinetic function given in eq. 11, $\Delta S_{\Delta \mathrm{T}}(q)$ as shown in Suppl. Fig. 18b, and $\Delta S^{\mathrm{bkg}}(\Delta t, q)$ as specified in eq. 3. The remaining data contain

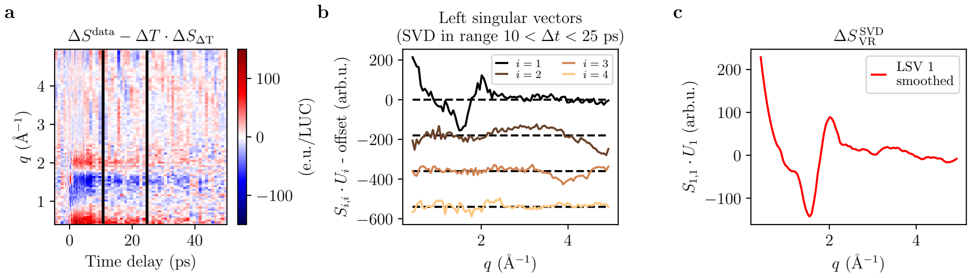

Suppl. Fig. 19 **a** Background artifact- and heat-subtracted difference scattering after fitting with an incomplete model considering two background artifact components and bulk solvent heating. The remaining data contain a signal component that appears for up to 40 ps and cannot be attributed to the optical Kerr effect [14] or structural changes of the excited state solute. An SVD in a time range of $10 < \Delta t < 25$ ps was calculated to extract a q-profile for this component. **b** The four strongest left-singular vectors (LSVs). The profile of the first LSV matches the signal observed in the data. LSVs 2 and 3 contain artifacts at low- and high-q. **c** The first LSV was smoothed using a Savitzky-Golay filter with an 11 point interval and a second order polynomial to reduce the noise on the curve.

a signal with a negative feature around $q = 1.5$ Å^{-1} and positive features around $q = 2$ Å^{-1} and below $q = 1$ Å^{-1} that persists for up to 40 ps and which can neither be explained by the signal response to the optical Kerr effect, for which a lifetime of 0.35 ± 0.21 ps has previously been reported [14], nor by excited state structural rearrangements in the solute and solvation-shell, as the analysis of the TAS data revealed an excited state lifetime of 2.6 ± 0.5 ps for HTI-J in acetonitrile (see Suppl. Note 3). To isolate the long-lived signal from the signal response to the OKE and the excited state dynamics of HTI-J, an SVD in a time delay range between 10 and 25 ps (see vertical black lines in Suppl. Fig. 19a) was calculated. After 10 ps, the signal response to the OKE is essentially fully decayed and more than 96% of the solute molecules have returned to the ground state, meaning that the signal contribution from excited state dynamics in HTI-J is expected to be negligible. Suppl. Fig. 19b shows the four strongest left-singular vectors (LSVs). The q-profile of the first LSV matches the features observed for the long-lived signal in the background artifact- and heat-subtracted difference scattering data. Suppl. Fig. 19c shows the first LSV after smoothing the noise using a Savitzky-Golay filter with an 11 point window and a

second-order polynomial. This curve was used as the initial signal component for the scattering response to vibrational relaxation (VR) of HTI-J in acetonitrile and denoted $\Delta S_{\mathrm{VR}}^{\mathrm{SVD}}$. The superscript SVD was added to distinguish this component from ΔS_{VR} shown in Fig. 5 in the main article, which is the optimized signal after global fitting (see Suppl. Note 6). The assignment of this component to solvent rearrangements due to vibrational relaxation of HTI-J in acetonitrile is discussed in the main article.

In addition to the first LSV, which was assigned as a signal component, the second and third LSVs also contain noticeable intensity features, in particular towards higher q. These features likely are remnant artifacts in the data, and are most evident by the fluctuating intensity features in the difference scattering signal in Suppl. Fig. 19a at high q. Since the signals investigated in this study are small, the presence of artifacts can affect the data modeling significantly. To minimize the influence of artifacts, a range of $0.6 < q < 3.8$ Å^{-1} was chosen for the subsequent data analysis.

5.3 Suppl. Note 5.3. Optical Kerr effect

Suppl. Fig. 20a shows the background artifact-, heat-, and VR-subtracted difference signal after fitting the isotropic difference scattering data with the signal response to bulk solvent heating, the signal attributed to vibrational relaxation, and the two background artifact components:

$$\Delta S_{\mathrm{fit}}(\Delta t, q) = \Delta T(\Delta t) \cdot \Delta S_{\Delta \mathrm{T}}(q) + \gamma(\Delta t) \cdot \Delta S_{\mathrm{VR}}^{\mathrm{SVD}}(q) + \Delta S^{\mathrm{bkg}}(\Delta t, q), \quad (7)$$

with $\Delta T(\Delta t)$ and $\gamma(\Delta t)$ described by the kinetic function given in eq. 11 and 12, $\Delta S_{\Delta \mathrm{T}}(q)$ and $\Delta S_{\mathrm{VR}}^{\mathrm{SVD}}(q)$ as shown in Suppl. Fig. 18b and Suppl. Fig. 19c, and $\Delta S^{\mathrm{bkg}}(\Delta t, q)$ as specified in eq. 3. The data are shown in a time range from -1 to 10 ps to highlight the remaining signal response at early time delays. Suppl. Figs. 20c and d show the four strongest left- and right-singular vectors resulting from an SVD of the displayed data. Based on a qualitative comparison of the shape of the first LSV and the response time in the first RSV to the results reported by Ki et al. [14], the first LSV is assigned to the signal response to the optical Kerr effect. Suppl. Fig. 20b shows the first LSV after smoothing the noise using a Savitzky-Golay filter with an 11 point interval and a second order polynomial. The component is denoted $\Delta S_{\mathrm{OKE}}^{\mathrm{SVD}}$, where the superscript SVD was added to distinguish the component from ΔS_{OKE} shown in Fig. 5 in the main article, which is the optimized signal after global fitting (see Suppl. Note 6).

The second and third RSV also display a time-dependence. The fast response in the third RSV around time zero suggests that this component might be part of the signal response to the optical Kerr effect. The second RSV shows a slower rise around time zero and a decay on a picosecond time scale, which is reminiscent of the excited state lifetime of 2.6 ± 0.5 ps of HTI-J in acetonitrile obtained from the analysis of the TAS data (see Suppl. Note 3). The second SVD component is further discussed in Suppl. Note 5.4.

5.4 Suppl. Note 5.4. Solvation-shell structural rearrangement

In Suppl. Fig. 20, it can be seen that the second RSV after SVD of the background artifact-, heat-, and VR-subtracted difference signal shows a time-dependence with a

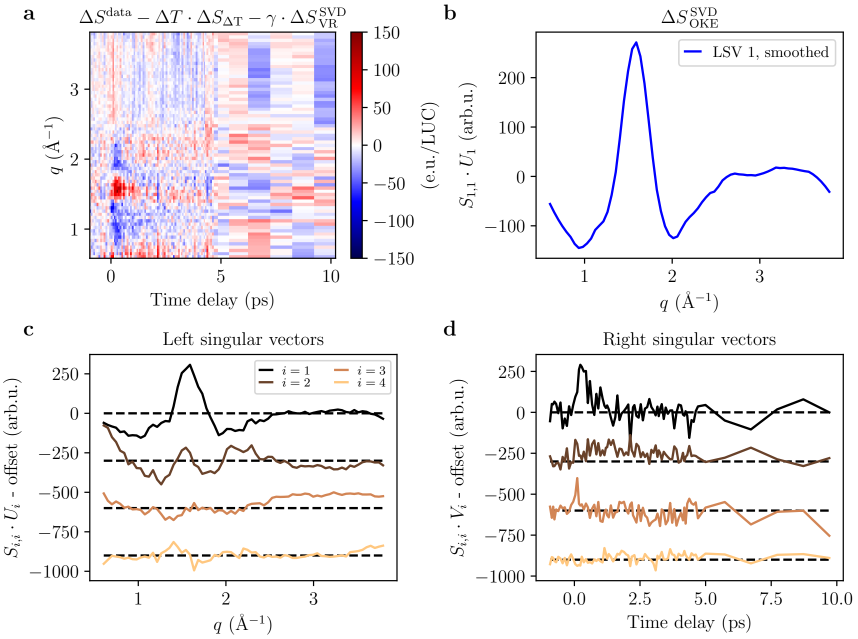

Suppl. Fig. 20 **a** Background artifact-, heat-, and VR-subtracted difference scattering after fitting with an incomplete model considering two background artifact components and the signal response to bulk solvent heating and vibrational relaxation. The remaining data are shown in a time range from -1 to 10 ps to highlight the signal response at early time delays. **c** and **d** show the four strongest LSVs and RSVs resulting from an SVD of the displayed data. **b** The first LSV is assigned to the signal response to the optical Kerr effect based on comparison of the signal shape and the response time captured by the first RSV to results reported by Ki et al. [14]. The noise on the first LSV was smoothed using a Savitzky-Golay filter with an 11 point interval and a second order polynomial.

rise around time zero and a decay within a few picoseconds. The response time of this component resembles the excited state lifetime of 2.6 ± 0.5 ps of HTI-J in acetonitrile obtained from the analysis of the TAS data (see Suppl. Note 3). Suppl. Fig. 21 shows the second LSV compared to difference scattering signals calculated from molecular dynamics (MD) simulations (see Suppl. Notes 9 and 10.1). The shape of the simulated signal for a twisting of the solute and a response of the solvation-shell to intramolecular charge transfer ("Charge transfer + twist") agrees with the second LSV, while the simulated signal for only a geometric rearrangement of the solute without a charge transfer ("Twist-only") does not fit the data. The difference between this model and the experimental data is particularly visible for $q < 1.5$ Å^{-1}, where the contribution of the changes in the solvent shell has the greatest impact.

While the observation of this component in the data after fitting with an incomplete model strongly supports the presence of a signal arising from solvation-shell structural rearrangements in response to population of an intermolecular charge transfer excited state, the LSV differs somewhat from the simulated signal for $q < 1.6$ Å^{-1} and for $q > 3$ Å^{-1}. The 1.6 Å^{-1} feature matches with the peak position of the component assigned to the signal response to the optical Kerr effect (see Suppl. Fig. 20b) and

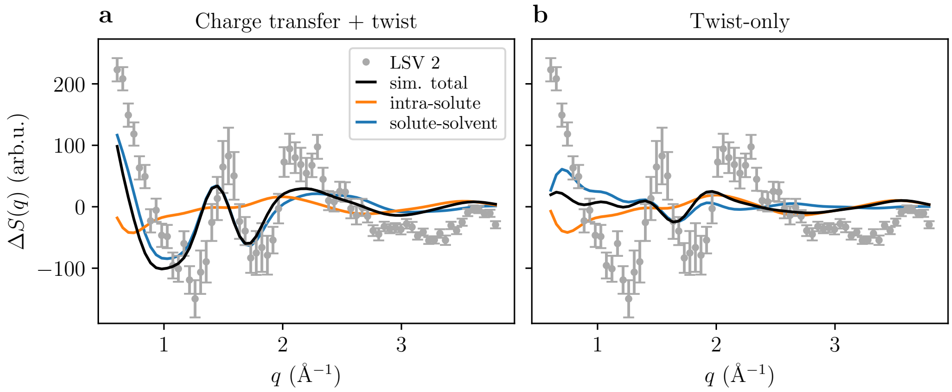

Suppl. Fig. 21 Second SVD component (LSV 2) of the data after fitting with an incomplete model (see Suppl. Fig. 20; error bars from polynomial noise estimation [22] described in Suppl. Note 4.7) compared to the difference scattering signal predicted from MD simulations (see Suppl. Notes 9 and 10.1). The black sim. total curve is the sum of the signals calculated from intra-solute (orange) and solute-solvent (blue) radial distribution functions (RDFs). **a** The simulated signal for a geometric rearrangement of the solute and a response of the solvation-shell to intramolecular charge transfer ("Charge transfer + twist") matches the shape of LSV 2. **b** The simulated signal for only a geometric rearrangement of the solute without charge transfer ("Twist-only") does not agree with the data. The mismatch between simulation and data is especially clear for $q < 1.5$ Å^{-1}, where the contribution of the changes in the solvent shell is most significant.

the differences for $q < 1.6$ Å^{-1} can be explained by the components not being well-separated in the singular vectors. The deviation at high-q is attributed to artifacts in the data. A more accurate estimate of the solvation-shell structural rearrangement (SSR) component, ΔS_{SSR}, is obtained in the global fitting presented in the next sections using the simulated signal as initial guess.

6 Suppl. Note 6. Global fitting of the TR-XSS data

The data analysis of the time-resolved X-ray scattering data is inspired by the global fitting strategies commonly employed in the analysis of transient spectroscopic data [5, 27]. Algorithm 1 summarizes the global fitting approach applied to the TR-XSS data, using the fit function

$$\Delta\mathbf{S}^{\mathrm{fit}} = \Delta\mathbf{S}^{\mathrm{model}} + \Delta\mathbf{S}^{\mathrm{bkg}} = \mathbf{A}\cdot\mathbf{C} + \mathbf{B}\cdot\mathbf{D} \tag{8}$$

in matrix form (see also eqs. 1 and 2 in the main article).

The parameters for the initial kinetic profiles, contained in $\mathbf{A}_0$, are reported in Suppl. Table 3 and the kinetic functions are described in Suppl. Note 7. The initial q-profiles, contained in $\mathbf{C}_0$, are given by the signal components determined from the experimental data as described in Suppl. Note 5: $\Delta S_{\Delta\mathrm{T}}$, $\Delta S^{\mathrm{SVD}}_{\mathrm{VR}}$, $\Delta S^{\mathrm{SVD}}_{\mathrm{OKE}}$, as well as the simulated difference scattering signal for solute and solvation-shell structural rearrangements (see Suppl. Note 10.1). The fitting uses regularization as explained in Suppl. Note 6.1,

Algorithm 1 Global fitting of time-resolved X-ray scattering data

Input:
$M \times N$ matrix $\Delta\mathbf{S}^{\text{data}}$ of time-dependent difference scattering data

Initialize:
$M \times K$ matrix $\mathbf{A}_0$ of K initial kinetic profiles depending on parameters $\{a_i\}, \{\tau_i\}$
$K \times N$ matrix $\mathbf{C}_0$ of K initial signal q-profiles
$K \times K$ diagonal matrix $\mathbf{W}$ of regularization parameters
$M \times L$ matrix $\mathbf{B}_0$ of L initial background artifact components amplitudes
$L \times N$ matrix $\mathbf{D}$ of L background artifact components
Noise estimate σ

$\mathbf{A} \leftarrow \mathbf{A}_0$, $\mathbf{B} \leftarrow \mathbf{B}_0$
repeat
Update A ▷ Outer loop kinetic profiles
Optimize B ▷ Inner loop background amplitudes
Fit $\Delta\mathbf{S}^{\text{data}} - \mathbf{A} \cdot \mathbf{C}_0$ with $\Delta\mathbf{S}^{\text{bkg}}$ for each time delay independently
until $\left\| \mathcal{L}\left(\frac{\Delta\mathbf{S}^{\text{data}} - \mathbf{A} \cdot \mathbf{C}_0 - \mathbf{B} \cdot \mathbf{D}}{\sigma} \right) \right\|^2$ is minimized to within desired tolerance

$\mathbf{C} \leftarrow \mathbf{C}_0$
Optimize C ▷ Optimize q-profiles
Solve $\min_{\mathbf{C}} \{ \|\Delta\mathbf{S}^{\text{data}} - \mathbf{A} \cdot \mathbf{C} - \mathbf{B} \cdot \mathbf{D}\|^2 + \|\mathbf{W} \cdot (\mathbf{C} - \mathbf{C}_0)\|^2 \}$

Optimize A ▷ Optimize kinetic profiles
Solve $\min_{\mathbf{A}} \left\| \mathcal{L}\left(\frac{\Delta\mathbf{S}^{\text{data}} - \mathbf{A} \cdot \mathbf{C} - \mathbf{B} \cdot \mathbf{D}}{\sigma} \right) \right\|^2$

Output: Optimized **A**, **C**, and **B**

where also the regularization parameters are specified. The determination of the two background profiles in **D** is described in Suppl. Note 4.6.

In the algorithm, **A**, **B**, and **C** are optimized to minimize the square of the residual between the data and the fit function, while **D**, containing the background profiles, is kept constant. $\mathcal{L}$ denotes the loss function, such as *linear* for the standard least-squares problem or more outlier-resistant options like *cauchy* or *arctan*. In the present global fitting, the arctan loss function was used. The optimized kinetic parameters are summarized in Suppl. Table 3 and the corresponding kinetic functions are shown in Suppl. Fig. 22, while the optimized q-profiles are shown in Fig. 5 in the main article. $\Delta S_{\Delta\text{T}}$ was fully constrained and not optimized.

6.1 Suppl. Note 6.1. Regularization

The optimization of **C** (q-profiles) is performed by solving a regularized least squares problem, where the objective is:

$$\min_{\mathbf{C}} \{ \|\Delta\mathbf{S}' - \mathbf{A} \cdot \mathbf{C}\|^2 + \|\mathbf{W} \cdot (\mathbf{C} - \mathbf{C}_0)\|^2 \}. \tag{9}$$

Here, $\Delta \mathbf{S}' = \Delta \mathbf{S}^{\text{data}} - \mathbf{B} \cdot \mathbf{D}$ is the background subtracted data, $\mathbf{C}_0$ is the matrix of initial q-profiles, and $\mathbf{W}$ is a diagonal matrix with the square roots of the regularization parameters, $\sqrt{\lambda_k}$, on the diagonal:

$$\mathbf{W} = \begin{pmatrix} \sqrt{\lambda_1} & 0 & \cdots & 0 \\ 0 & \sqrt{\lambda_2} & \cdots & 0 \\ \vdots & \vdots & \ddots & \vdots \\ 0 & 0 & \cdots & \sqrt{\lambda_K} \end{pmatrix}. \tag{10}$$

For the global fitting of the HTI-J in acetonitrile TR-XSS data, the q-profile for the bulk solvent heating was fully constrained by setting $\lambda_{\Delta\text{T}} = 10$, while no constraint was placed on the OKE, SSR, and VR components ($\lambda_{\text{OKE}} = \lambda_{\text{SSR}} = \lambda_{\text{VR}} = 0$).

6.2 Suppl. Note 6.2. Global fitting results

Suppl. Table 3 summarizes the parameters obtained from the global fitting of the TR-XSS data. The optimized kinetic profiles are visualized in Suppl. Fig. 22. The time

Suppl. Table 3 Parameters obtained from a global fitting of the model given in eq. 2 in the main article to the time-resolved isotropic difference scattering signal of the HTI-J molecule photoexcited in acetonitrile. Times are in ps.

Parameter	Value	Initial value	Origin
a_{OKE}	0.6 ± 0.1	0.5	Signal response to optical Kerr effect
τ_{OKE}	0.4 ± 0.1	0.4	
a_{SSR}	0.2 ± 0.1	0.5	Solvation-shell structural rearrangement
τ^1_{SSR}	0.3 ± 0.1	0.3	
τ^2_{SSR}	3 ± 1	2.6	
a_{VR}	0.7 ± 0.1	0.5	Solute vibrational relaxation
τ^1_{VR}	1.6 ± 0.3	2	
τ^2_{VR}	15 ± 2	14	
$a^1_{\Delta\text{T}}$	0.32 ± 0.01	0.3	Fast solvent heating
$\tau^1_{\Delta\text{T}}$	0.2 ± 0.1	1	
$a^2_{\Delta\text{T}}$	0.90 ± 0.01	0.9	Slow solvent heating
$\tau^2_{\Delta\text{T}}$	13.5 ± 0.5	14	

constants are also reported in Table 1 of the main article, where they are discussed and interpreted in detail. The amplitude of the ΔS_{SSR} component, a_{SSR}, was limited to an upper value of 1 to ensure the stability of the global fitting.

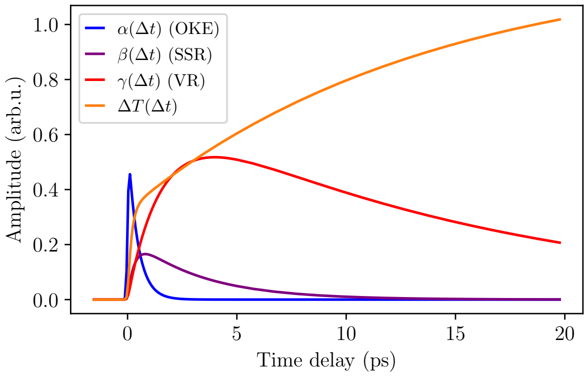

Suppl. Fig. 22 Kinetic profiles resulting from global fitting of the time-resolved X-ray scattering data with the kinetic functions described in Suppl. Note 7.

6.2.1 Suppl. Note 6.2.1. Heat-subtracted difference signal at selected time delays

Suppl. Fig. 23a shows the heat-subtracted isotropic difference scattering signal in a time range from -3 to 20 ps. Background artifact components identified as part of the global fitting were also subtracted. Vertical lines indicate the time delays for which $\Delta S(q)$ is shown in Suppl. Figs. 23b-d. Suppl. Figs. 23b-d show the data including errors from polynomial noise estimation [22], the corresponding heat-subtracted $\Delta S^{\mathrm{model}}(q)$ obtained from global fitting, and the contributions from $\Delta S_{\mathrm{OKE}}(q)$, $\Delta S_{\mathrm{SSR}}(q)$, and $\Delta S_{\mathrm{VR}}(q)$ at the selected time delays. Suppl. Fig. 23b shows the data, model and signal contributions at a time delay of 0.2 ps. At such early time delays, the signal response to the optical Kerr effect dominates, while $\Delta S_{\mathrm{SSR}}(q)$ and $\Delta S_{\mathrm{VR}}(q)$ contribute only weakly. Suppl. Fig. 23c shows the data, model and signal contributions at a time delay of 0.8 ps. Around this time delay, the signal contribution from the solvation-shell rearrangement in response to intramolecular charge transfer in the HTI-J solute is at its maximum. Since the observed processes occur simultaneously, $\Delta S_{\mathrm{OKE}}(q)$ and $\Delta S_{\mathrm{VR}}(q)$ contribute as well. Suppl. Fig. 23d shows the data, model and signal contributions at a time delay of 4.0 ps. At later time delays, the heat-subtracted data is dominated by the signal arising from vibrational relaxation of the solute in the ground state. $\Delta S_{\mathrm{OKE}}(q)$ is fully decayed at this time delay, while $\Delta S_{\mathrm{SSR}}(q)$ contributes weakly. The insets in Suppl. Figs. 23b-d specify the reduced chi-squared (χ^2) of the fit at the selected time delays. The reduced χ^2 are somewhat larger than 1. Rather than considering this to be due to an incomplete model, we attribute a χ^2 larger than 1 to remaining background artifacts in the experimental data that were not considered in the global fitting to avoid overfitting.

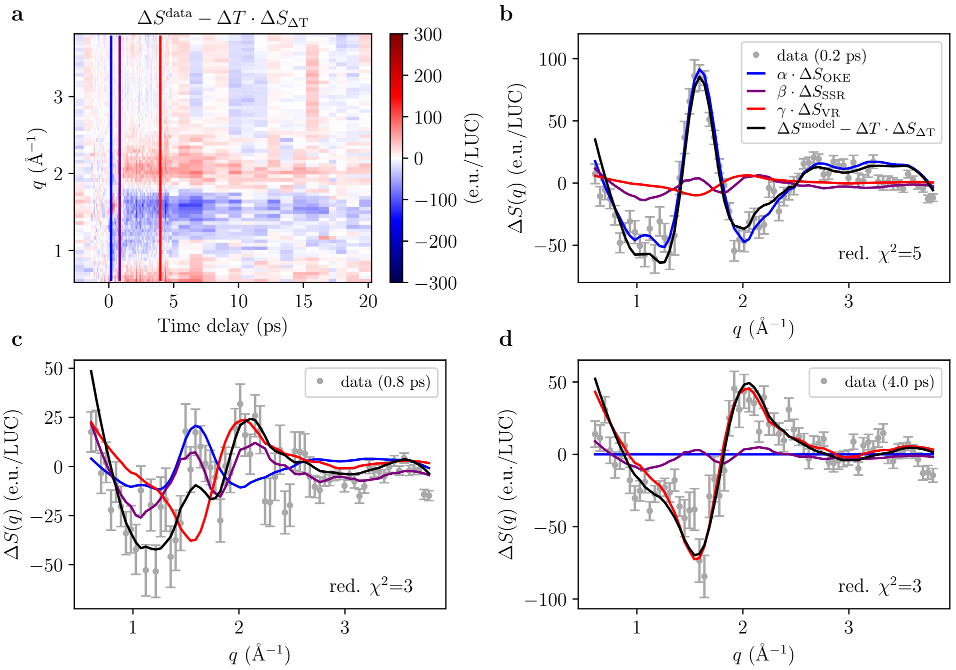

Suppl. Fig. 23 **a** Background artifact- and heat-subtracted isotropic difference scattering signal with vertical lines indicating time slices at which the data are plotted in **b-d**. Data at the respective time delays are shown by gray markers with error bars from polynomial noise estimation [22] (see Suppl. Note 4.7). Blue, purple and red lines show the contributions of $\Delta S_{\rm OKE}$, $\Delta S_{\rm SSR}$, and $\Delta S_{\rm VR}$ obtained through the global fitting. The black line corresponds to the heat- and background-subtracted model signal. **b** $\Delta S(q)$ at a time delay of 0.2 ps, where the signal is dominated by the OKE solvent response. **c** $\Delta S(q)$ at a time delay of 0.8 ps, where the contribution of $\Delta S_{\rm SSR}$ is around its maximum, but also $\Delta S_{\rm OKE}$ and $\Delta S_{\rm VR}$ contribute to the signal. **d** At a time delay of 4.0 ps, the data is dominated by $\Delta S_{\rm VR}$ with a small contribution from $\Delta S_{\rm SSR}$, while $\Delta S_{\rm OKE}$ is fully decayed at this time delay. Text insets specify reduced chi-squared values of the fit.

6.2.2 Suppl. Note 6.2.2. Charge transfer initial guess for SSR component

The results presented in the main article and in Suppl. Table 3 are obtained using the simulated signal for intramolecular charge transfer and twisting of the solute ("Charge transfer + twist", see Suppl. Fig. 32a) as initial guess for the $\Delta S_{\rm SSR}$ component in the global fitting. The global fitting yields similar results when using the simulated difference signal for intramolecular charge transfer without twisting ("Charge transfer", see Suppl. Fig. 32b). Suppl. Figs. 24a-b show the background artifact-, heat-, VR-, and OKE-subtracted difference scattering data, leaving only the signal response to the solvation-shell rearrangement (SSR). For the data shown in Suppl. Fig. 24a, charge transfer and twisting of the solute was used as initial guess for $\Delta S_{\rm SSR}$, while in Suppl. Fig. 24b intramolecular charge transfer without twisting was used as initial guess. Suppl. Figs. 24c-d show an average of the data in a time delay range between 0 and 5 ps as

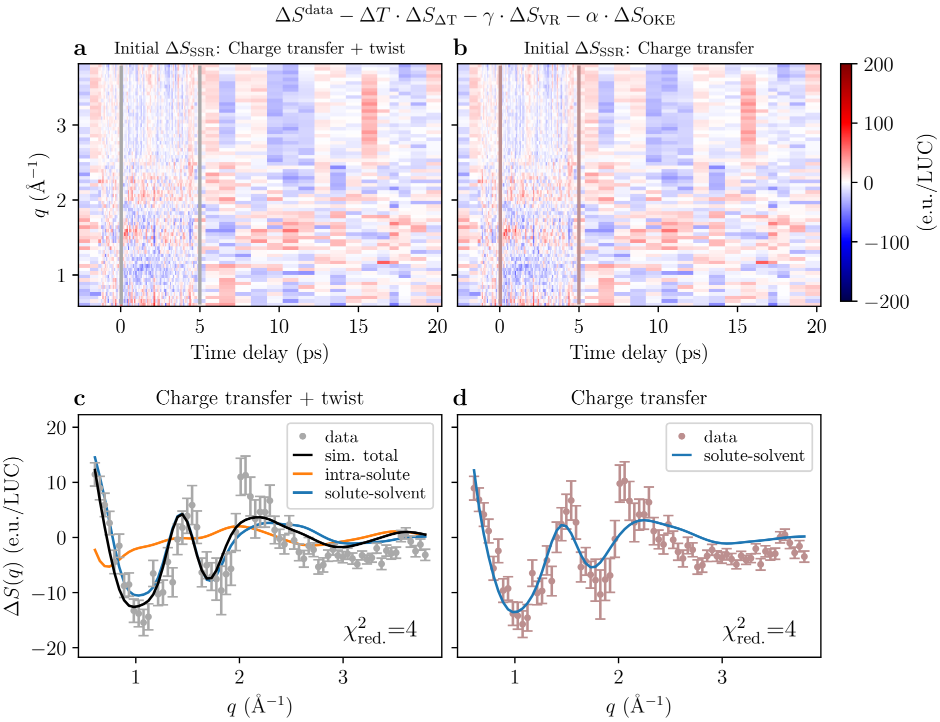

Suppl. Fig. 24 **a-b** Background artifact-, heat-, VR-, and OKE-subtracted isotropic difference scattering signal. **a** The simulated signal for charge transfer and twisting of the solute was used as initial guess for ΔS_{SSR} in the global fitting. **b** The simulated signal for charge transfer without twisting of the solute was used as initial guess for ΔS_{SSR} in the global fitting. Vertical lines indicate the time delay range in which the data displayed in **c-d** were averaged (error bars from polynomial noise estimation [22] described in Suppl. Note 4.7). **c** Averaged data compared to the simulated signal for charge transfer and twisting of the solute. The black sim. total curve is the sum of the signals calculated from intra-solute (orange) and solute-solvent (blue) radial distribution functions. **d** Averaged data compared to the simulated signal for charge transfer without twisting of the solute. In this case there is no intra-solute contribution and only the solute-solvent simulated signal is present. Insets specify reduced χ^2 values.

indicated by the vertical lines in Suppl. Figs. 24a-b. Since different initial ΔS_{SSR} were used in the global fitting, the data displayed in Suppl. Figs. 24c and d are not identical, which is reflected in the different colors for the data points and errorbars. Errors were estimated by polynomial noise estimation [22]. While not identical, the averaged data are similar and dominated by the solvation-shell response to the intramolecular charge transfer and not the twisting of the solute. Some differences can be seen above 2 Å^{-1}. Between 2 and 3 Å^{-1}, the data is shifted to lower q with respect to the solute-solvent simulated curves. In the case of charge transfer and twist, inclusion of the intra-solute term improves the agreement between the data and the simulation in this range, as well as for the slight peak around 3.6 Å^{-1}. In case of charge transfer alone, the agreement around 1.4 Å^{-1} is marginally better than for charge transfer and twist and in both

cases, the reduced $\chi^2 = 4$, meaning that a quantitative distinction between the two models is not possible given the present signal-to-noise ratio on the data.

6.2.3 Suppl. Note 6.2.3. Twist-only initial guess for SSR component

In a similar procedure as described above for charge transfer and twisting of the solute and charge transfer only, Suppl. Fig. 25 shows the average data between 0 and 5 ps after subtracting modeled contributions from background artifacts, bulk solvent heating, vibrational relaxation and the signal response to the optical Kerr effect when using the simulated signal for twisting of the solute without charge transfer ("Twist-only", see Suppl. Fig. 32c) as initial guess for the ΔS_{SSR} component.

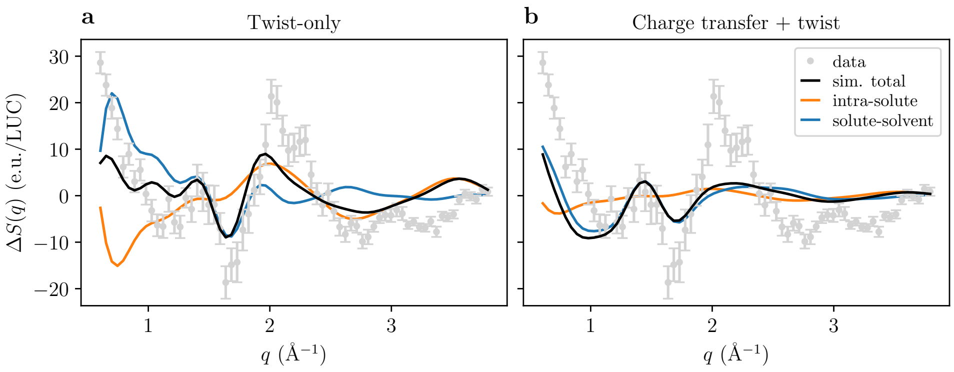

Suppl. Fig. 25 Average (0-5 ps) of the background artifact-, heat-, VR-, and OKE-subtracted isotropic difference scattering signal after global fitting with the simulated signal for twisting of the solute without charge transfer as initial guess for the ΔS_{SSR} component (error bars from polynomial noise estimation [22] described in Suppl. Note 4.7). **a** Averaged data compared to the simulated signal for twisting of the solute without charge transfer. The black sim. total curve is the sum of the signals calculated from intra-solute (orange) and solute-solvent (blue) radial distribution functions. **b** Averaged data compared to the simulated signal for charge transfer and twisting of the solute. For $q < 1.5$ Å^{-1}, where the contribution of the changes in the solvent shell has the greatest impact, the shape of the average experimental data resembles the simulated signal for the charge transfer and twist model.

Suppl. Fig. 25a compares the average data to the simulated signals for twisting of the solute without charge transfer. While the shape of the signals somewhat agrees at high q, the change in the signal for $q < 1.5$ Å^{-1}, where the solute-solvent term strongly impacts the signal shape, matches better with the shape of the simulated signal including intramolecular charge transfer as can be seen in Suppl. Fig. 25b.

7 Suppl. Note 7. Kinetic functions

The temporal evolution of the signal components in the time-resolved X-ray solution scattering data is modeled by kinetic functions consisting of rising and decaying exponential functions convolved with the instrument response function (IRF) of the

experiment. In the present case, the functions depend on the time delay, Δt, and are parametrized by the time of signal onset, t_0, the Gaussian width of the IRF, σ_{IRF}, time constants, τ_i, and amplitudes, a_i. Time zero and the Gaussian width of the IRF were determined by modeling the signal response to the optical Kerr effect in the anisotropic difference scattering data by eq. 13 as explained in Suppl. Note 4.4. These parameters were then fixed to $t_0 = 0$ ps and $\sigma_{\mathrm{IRF}} = 0.05$ ps when modeling the experimental data with eqs. 11, 12 and 13. Time zero is set to 0 as the time axis of the experimental data was shifted to display a signal onset at $\Delta t = 0$ ps and it is expected that all signal contributions arise with photoexcitation at time zero.

7.1 Suppl. Note 7.1. Double exponential rise

The signal arising from the temperature increase in the bulk solvent is modeled by a double exponential rise broadened by the IRF:

$$\begin{aligned} K(\Delta t) = \frac{a_1}{2}\Bigg[&\left(1+\operatorname{erf}\left(\tfrac{\Delta t - t_0}{\sqrt{2}\sigma_{\mathrm{IRF}}}\right)\right) \\ &- \exp\left(\tfrac{\sigma_{\mathrm{IRF}}^2 - 2(\Delta t - t_0)\tau_1}{2\tau_1^2}\right)\left(1-\operatorname{erf}\left(\tfrac{\sigma_{\mathrm{IRF}}^2-(\Delta t - t_0)\tau_1}{\sqrt{2}\sigma_{\mathrm{IRF}}\tau_1}\right)\right)\Bigg] \\ &+ \frac{a_2}{2}\Bigg[\left(1+\operatorname{erf}\left(\tfrac{\Delta t - t_0}{\sqrt{2}\sigma_{\mathrm{IRF}}}\right)\right) \\ &- \exp\left(\tfrac{\sigma_{\mathrm{IRF}}^2 - 2(\Delta t - t_0)\tau_2}{2\tau_2^2}\right)\left(1-\operatorname{erf}\left(\tfrac{\sigma_{\mathrm{IRF}}^2-(\Delta t - t_0)\tau_2}{\sqrt{2}\sigma_{\mathrm{IRF}}\tau_2}\right)\right)\Bigg], \end{aligned} \tag{11}$$

where a_1 and a_2 are amplitudes, τ_1 and τ_2 are rise times, Δt is the time delay, $t_0 = 0$ ps is time zero, and $\sigma_{\mathrm{IRF}} = 0.05$ ps is the Gaussian width of the IRF. Exp stands for exponential function, and erf for error function.

7.2 Suppl. Note 7.2. Single exponential rise and decay

The signal responses to vibrational relaxation, ΔS_{VR}, and the solvation-shell structural rearrangement, ΔS_{SSR}, are modeled by the sum of a single exponential rise and a single exponential decay broadened by the IRF:

$$\begin{aligned} K(\Delta t) = \frac{a}{2}&\left(1+\operatorname{erf}\left(\tfrac{\Delta t - t_0}{\sqrt{2}\sigma_{\mathrm{IRF}}}\right)\right) \\ &- \frac{a}{2}\exp\left(\tfrac{\sigma_{\mathrm{IRF}}^2 - 2(\Delta t - t_0)\tau_1}{2\tau_1^2}\right)\left(1-\operatorname{erf}\left(\tfrac{\sigma_{\mathrm{IRF}}^2-(\Delta t - t_0)\tau_1}{\sqrt{2}\sigma_{\mathrm{IRF}}\tau_1}\right)\right) \\ &- \frac{a}{2}\left[1-\exp\left(\tfrac{\sigma_{\mathrm{IRF}}^2 - 2(\Delta t - t_0)\tau_2}{2\tau_2^2}\right)\left(1-\operatorname{erf}\left(\tfrac{\sigma_{\mathrm{IRF}}^2-(\Delta t - t_0)\tau_2}{\sqrt{2}\sigma_{\mathrm{IRF}}\tau_2}\right)\right)\right], \end{aligned} \tag{12}$$

where a is the amplitude, τ_1 and τ_2 are the rise and decay time, Δt is the time delay, $t_0 = 0$ ps is time zero, and $\sigma_{\mathrm{IRF}} = 0.05$ ps is the Gaussian width of the IRF. Exp stands for exponential function, and erf for error function.

7.3 Suppl. Note 7.3. Step-function rise and single exponential decay

The signal response to the optical Kerr effect in the isotropic and anisotropic difference scattering data are modeled by an instantaneous rise (step-function) and a single exponential decay broadened by the IRF (see 14 for the fitting of ΔS_2):

$$K(\Delta t) = \frac{a}{2} \exp\left(\frac{\sigma_{\mathrm{IRF}}^2 - 2(\Delta t - t_0)\tau}{2\tau^2}\right)\left[1 - \mathrm{erf}\left(\frac{\sigma_{\mathrm{IRF}}^2 - (\Delta t - t_0)\tau}{\sqrt{2}\,\sigma_{\mathrm{IRF}}\tau}\right)\right] + C, \tag{13}$$

where a is the amplitude, τ is the decay time, Δt is the time delay, $t_0 = 0$ ps is time zero, and $\sigma_{\mathrm{IRF}} = 0.05$ ps is the Gaussian width of the IRF. C stands for a constant offset that is included in the modeling of the signal response in the anisotropic difference scattering data, ΔS_2, but is set to $C = 0$ when modeling the signal response to the optical Kerr effect in the isotropic data. Exp stands for exponential function, and erf for error function.

8 Suppl. Note 8. Energy deposition into the solvent

The change in temperature in the solvent expected from the absorption of a photon of a specific wavelength can be calculated by

$$\Delta T = \frac{E_{\mathrm{photon}} \cdot N_{\mathrm{A}}}{C_V} \cdot \frac{M \cdot c}{\rho} \cdot \alpha,$$

where E_{photon} is the photon energy corresponding to the excitation wavelength, N_{A} is Avogadro constant, C_V is the heat capacity of the solvent at constant volume, M is the molar mass of the solvent, ρ is the density of the solvent, c is the concentration of the solute and α is the fraction of excited molecules.

For the presented scattering experiments with laser excitation at 470 nm, $E_{\mathrm{photon}} = 2.64$ eV. The sample was a 3 mM solution of HTI-J in acetonitrile, so $C_V = 63.53$ J/mol/K [28], $M = 41.05$ g/mol, $\rho = 786$ g/L [18], and $c = 3$ mM. Given these parameters and setting $\alpha = 1$, corresponding to 100% excitation fraction, the maximum expected change in solvent temperature based on one-photon excitation is around 0.6 K. The temperature change observed from fitting the experimental data is around 1.2 K, which is double the maximum expected temperature change.

The difference is mainly attributed to multiphoton excitation of the solute as a consequence of the laser fluence at the upper end of the linear-response region. Multiphoton excitation is a nonlinear optical process where the solute absorbs two or more photons and is promoted to a higher lying excited state. The excess energy is dissipated to the solvent through vibrational relaxation in the lowest lying excited state, leading to an additional temperature increase in the bulk solvent. In the present study, this is reflected in the rapid onset of the solvent heating response with a time constant of $\tau_{\Delta \mathrm{T}}^1 \sim 0.2$ ps, as discussed in the main text and previously observed in TR-XSS studies conducted at similar excitation fluence [14, 29, 30].

A direct excitation of the solvent is not expected. Ki et al. [14] report no evidence of such effects in acetonitrile when using a 400 nm laser with a fluence ∼50% higher

than that used in the present study. This is consistent with the ∼12 eV ionization threshold for acetonitrile [18], which would require a four-photon process at 400 nm (3.1 eV per photon). Direct photoionization of the HTI-J solute molecules at 470 nm (2.6 eV) would similarly require a three-photon process, given that related stilbene and indigo compounds exhibit photoionization thresholds of approximately 7.5 eV and 6.9 eV, respectively [18, 31]. Considering these thresholds and the laser fluence scans, which show only weak nonlinear behavior (see Suppl. Fig. 8), we regard photoionization processes as unlikely to influence the reported results.

We therefore conclude that multiphoton excitation of the HTI-J solute and rapid relaxation to the lowest excited state is responsible for the large temperature increase and the rapid onset of the solvent-heating response. Apart from these observations, no additional signal contributions arising from the higher laser fluence in the TR-XSS experiments compared to the TAS could be discerned in our analysis.

9 Suppl. Note 9. Electronic structure calculations

9.1 Suppl. Note 9.1. Basis set convergence

Suppl. Fig. 26 shows an analysis of the basis set convergence for the orbital-optimized excited state density functional calculations of HTI-J in acetonitrile using the CAM-B3LYP functional and the conductor-like polarizable continuum model (CPCM) for the solvent. The vertical excitation energy, single bond twist dihedral angle, d_1, of the

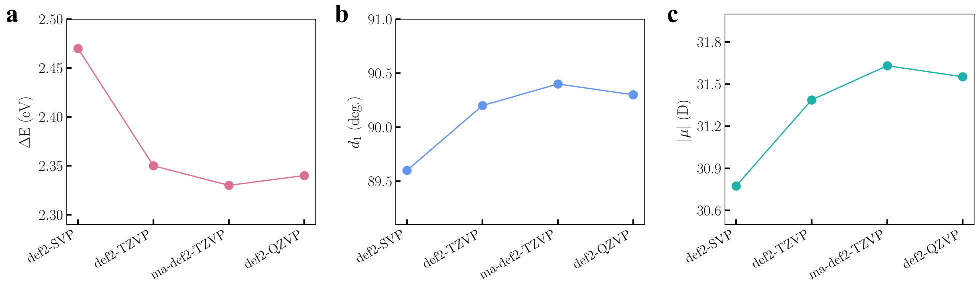

Suppl. Fig. 26 Basis set convergence for **a** the vertical excitation energy, **b** dihedral angle of the single bond twist of the optimized excited state geometry, and **c** magnitude of the dipole moment, for the S1 excited state of HTI-J in acetonitrile using orbital-optimized calculations with CAM-B3LYP/CPCM.

optimized geometry, and the magnitude of the dipole moment, for the first exited state, S1, are plotted as a function of the basis set. The single bond twist dihedral angle d_1 is defined by atoms C2, C3, C4 and C5 as shown in Suppl. Fig. 27. The changes between def2-TZVP, ma-def2-TZVP, and def2-QZVP are small for all the considered quantities. Satisfactory convergence is reached with the minimally augmented ma-def2-TZVP basis set, which includes diffuse functions and is chosen for all calculations discussed further here and in the main article.

9.2 Suppl. Note 9.2. DFT calculations

The optimized ground state minimum geometry corresponds to the Z isomer and is shown in Suppl. Fig. 27 together with the optimized geometry of the E isomer.The two isomers differ by a twist around the carbon-carbon double bond between the thioindigo and stilbene fragments, as described by the dihedral angle d_2 (atoms C1, C2, C3, and H1).

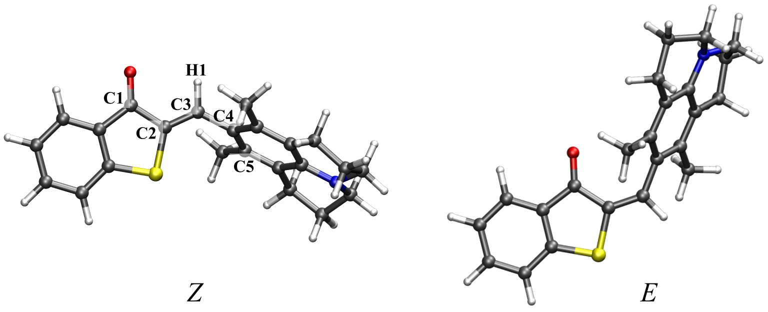

Suppl. Fig. 27 The ground state structures for the Z and E isomers for the HTI-J optimized with CAM-B3LYP/ma-def2-TZVP/CPCM. Atom colors: C gray, H white, N blue, O red, S yellow. The labeled atoms define the relevant dihedral angles: single bond twist d_1 (C2-C3-C4-C5), and double bond twist d_2 (C1-C2-C3-H1).

The excitation energy and the oscillator strength for the three lowest excited states obtained from linear-response TDDFT calculations with the CPCM implicit solvent model for acetonitrile are shown in Suppl. Table 4. The first excited state shows a large

Suppl. Table 4 Vertical excitation energy and oscillator strength of the first excited state of HTI-J in acetonitrile obtained from linear-response TDDFT and orbital-optimized (OO) calculations with CAM-B3LYP/ma-def2-TZVP/CPCM. TDDFT results for the second and third excited states are also shown.

	ΔE (eV)			f
	OO	TDDFT	exp.	TDDFT
S1	2.33 [a]	3.08	2.64	0.43
S2	-	3.52	-	0.01
S3	-	3.83	-	0.02

[a] including full (fast and slow) solvent response within the CPCM model in the excited state

oscillator strength, and has an excitation energy closest to the position of the maximum of the experimental absorption spectrum, 470 nm (2.64 eV), whereas the higher-energy

states have oscillator strengths close to zero, indicating that the one-photon excitation by the pump pulse in the TAS and TR-XSS experiments is to the lowest excited state.

The equilibrium geometries and partial charges used in the MD simulations were obtained from ground state and orbital-optimized excited state calculations. Both the ground and S1 geometries were fully optimized in acetonitrile described with the CPCM implicit solvent model. Orbital-optimized calculations with the CAM-B3LYP functional have been shown to produce accurate excitation energies and charge distributions for long-range charge transfer excited states in organic molecules [32, 33]. Suppl. Table 4 shows the vertical excitation energy for the lowest excited state obtained with the orbital-optimized approach. For the excitation energy, the spin-purification formula is applied to obtain the energy of the singlet excited state E_s from the energies of spin-mixed and triplet calculations, E_m and E_t [34]:

$$E_\mathrm{s} = 2E_\mathrm{m} - E_\mathrm{t}\,. \tag{14}$$

where E_m is obtained in a calculation where one electron is excited in one spin channel, while E_t is obtained in a calculation where one electron is excited from one spin channel to the other. The excited state calculations include both the fast and slow response of the solvent in the implicit CPCM solvent model, representing the solvent being in equilibrium with the solute, instead of only the fast, electronic response corresponding to the vertical excitation. This might explain the underestimation of the vertical excitation energy by the calculations. Suppl. Table 5 shows the dihedral angles d_1 and d_2, and change in the magnitude of the dipole moment between the ground and S1 state both at the Franck-Condon (FC) geometry and the S1 minimum. The excited state geometry was optimized using the mixed-spin calculations only and the excited state dipole moment corresponds to the dipole moment of the spin-mixed solution. The S1 minimum geometry has $d_1 = 90.4°$, corresponding to a twisting to a

Suppl. Table 5 Change in the magnitude of the dipole moment, and single and double bond twist dihedral angles (d_1 and d_2) for the FC geometry and the optimized geometry of the S1 excited state of HTI-J in acetonitrile obtained from orbital-optimized calculations with CAM-B3LYP/ma-def2-TZVP/CPCM.

	FC	S1 min.
$\Delta\lvert\mu\rvert$ (D)	21.9	26.3
$\lvert d_1\rvert$ (deg.)	60.7	90.4
$\lvert d_2\rvert$ (deg.)	1.3	0.1

$\sim$90-degree geometry upon photoexcitation. The dipole moments for the ground state and S1 excited state at the FC geometry, obtained from calculations for the spin-mixed state, are shown in Suppl. Fig. 28. The large change in the dipole moment between the

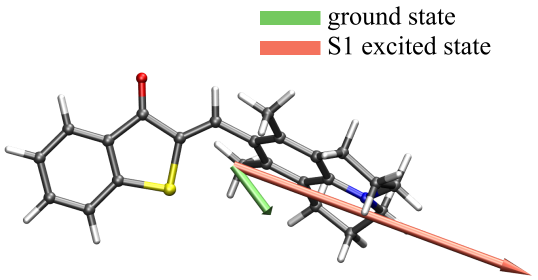

Suppl. Fig. 28 Electric dipole moments of the ground state (green) and the S1 excited state at the ground state geometry (orange), the latter obtained from orbital-optimized calculations. All calculations use CAM-B3LYP/ma-def2-TZVP/CPCM.

ground and S1 state shows that the excited state has strong charge transfer character.

Suppl. Table 6 Point charges in units of e of the oxygen, nitrogen and sulfur atoms, as well as total charges of the thioindigo and julolidine fragments for the ground state (GS) and S1 charge transfer excited state of HTI-J in acetonitrile used in the MD simulations.

	GS	S1
O	-0.62	-0.83
S	-0.09	-0.19
N	-0.05	0.33
thioindigo	-0.34	-1.02
julolidine	0.34	1.02

Suppl. Table 6 shows the point charges of three selected atoms, oxygen and sulfur of the thioindigo fragment, and nitrogen of the julolidine fragment, used for the HTI-J ground and excited states in the MD simulations. The point charges are obtained from the DFT optimized ground and S1 states using the CHELPG method [35]. An electronic charge of 0.72 e is transferred from the julolidine to the thioindigo fragment, making the former more positively charged and the latter more negatively charged. The partial charges for the three selected atoms show this charge transfer, the nitrogen having an especially large change in the partial charge, going from slightly negative to significantly positive.

9.3 Suppl. Note 9.3. CCSD(T) calculations

To validate the DFT results, additional calculations are performed using the domain based local pair-natural orbital (DLPNO) coupled cluster singles, doubles and perturbative triples (CCSD(T)) method [36, 37]. These calculations are performed for T1, the triplet state corresponding to S1. The T1 and S1 states differ only in the exchange interaction, so they are often expected to exhibit similar electronic character and approximately parallel potential energy surfaces, as observed, e.g., for azobenzene [38]. Since T1 is the lowest triplet state, ground state methods can be utilized, facilitating the inclusion of solvent effects. The CCSD(T) calculations were performed with ORCA, using the cc-pVDZ basis set [39, 40] and the perturbation theory energy singles (PTES) scheme [41] to include solvent effects within the CPCM model.

Suppl. Fig. 29 shows the energy of the T1 state as a function of the d_1 twisting angle compared to the energy of the S1 state from OO calculations using CAM-B3LYP/cc-pVDZ/CPCM. The CAM-B3LYP energies are from a relaxed S1 scan, and the points of the CCSD(T) curve are obtained from single-point calculations performed at these geometries. Both methods show a similar shape with a minimum at 90-degree twist, agreeing with the minimum S1 geometry with $d_1 = 90.4°$ from OO/CAM-B3LYP/ma-def2-TZVP calculations. The magnitude of the CCSD(T) dipole moment for T1 at the minimum of the scan, with $|d_1| = 90.0°$ and $|d_2| = 0.0°$, is 33.9 D, close to 31.6 D at the OO/CAM-B3LYP/ma-def2-TZVP S1 minimum with $|d_1| = 90.4°$ and $|d_2| = 0.1°$.

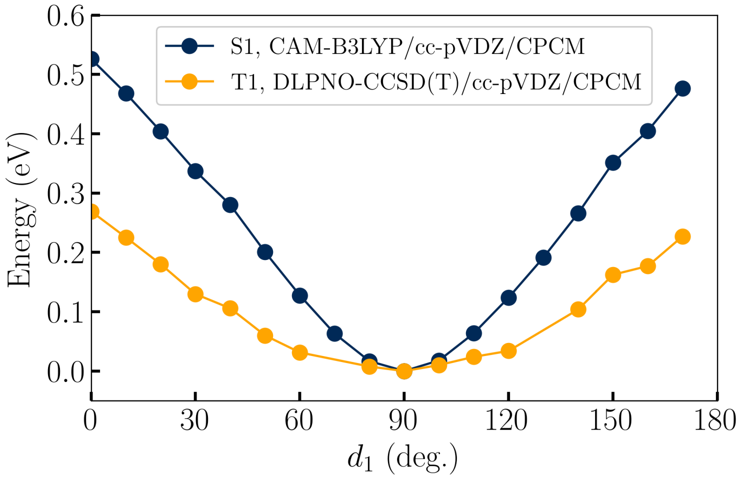

Suppl. Fig. 29 Energy along the d_1 single bond twist for the lowest triplet state (T1) from DLPNO-CCSD(T)/cc-pVDZ/CPCM calculations, and for the lowest singlet excited state (S1) from OO calculations using CAM-B3LYP/cc-pVDZ/CPCM. The energy is shown with respect to the T1 or S1 minimum energy geometry. The CAM-B3LYP energy is from a relaxed scan along d_1, and the CCSD(T) energy is from single-point calculations at these geometries.

10 Suppl. Note 10. Molecular dynamics simulations

10.1 Suppl. Note 10.1. Equilibrium molecular dynamics simulations

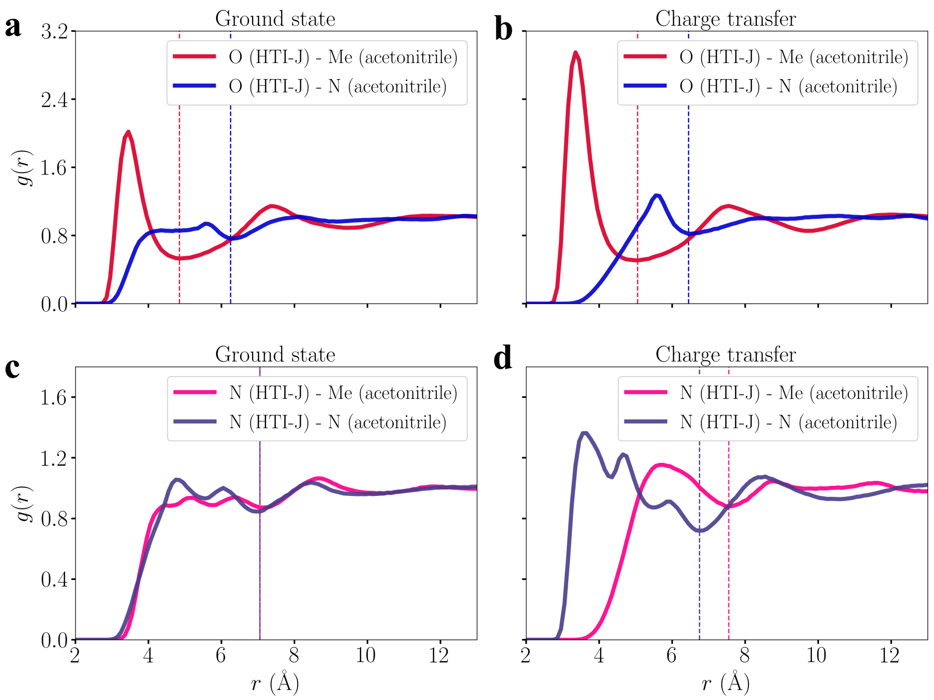

Suppl. Fig. 30 Solute-solvent radial distribution functions (RDFs) between the nitrogen and the methyl carbon sites of acetonitrile, and (a-b) the oxygen of HTI-J, and (c-d) the nitrogen of HTI-J, calculated from equilibrium molecular dynamics simulations of HTI-J in acetonitrile. (a,c) RDFs for HTI-J in the ground state. (b,d) RDFs for HTI-J in the excited state, modelled by modifying only the partial charges according to DFT calculations (see Suppl. Note 9.2). The excitation corresponds to charge transfer from the julolidine to the thioindigo group. Dashed lines mark the boundary of the first solvation shell used to compute the structural parameters reported in Suppl. Table 7.

Suppl. Fig. 30 shows representative solute-solvent radial distribution functions (RDFs) obtained from the equilibrium molecular dynamics simulations of the HTI-J molecule in acetonitrile. RDFs from two sets of simulations are compared, one where the HTI-J molecule is modelled in the ground state and one where it is modelled in the excited state by changing only the partial charges compared to the ground state based on the DFT calculations (see section 9.2) reflecting a charge transfer from the julolidine to the thioindigo group. Suppl. Table 7 reports key structural parameters extracted from the RDFs of Suppl. Fig. 30 as well as the RDFs of a set of MD simulations where both the partial charges and geometry are changed in the excited state based on the

Suppl. Table 7 The position of the first peak, $r_{g_{\max}}$, the mean peak of the first solvation shell, $\langle r \rangle$, and the coordination number for the first solvation shell, CN, obtained from solute-solvent radial distribution functions of different sets of equilibrium molecular dynamics simulations of the HTI-J molecule in acetonitrile: (i) HTI-J in the ground state (GS), (ii) HTI-J in the excited state modelled by changing only the partial charges (CT only), and (iii) HTI-J in the excited state modelled by changing the partial charges and the geometry (Twist + CT). $r_{g_{\max}}$ and $\langle r \rangle$ are reported in Å. The boundary of the first solvation shell used to compute the reported parameters is shown in Suppl. Fig. 30.

	O(HTI)-Me(ACN)			O(HTI)-N(ACN)			N(HTI)-Me(ACN)			N(HTI)-N(ACN)		
	$r_{g_{\max}}$	$\langle r \rangle$	CN	$r_{g_{\max}}$	$\langle r \rangle$	CN	$r_{g_{\max}}$	$\langle r \rangle$	CN	$r_{g_{\max}}$	$\langle r \rangle$	CN
GS	3.5	3.8	3.9	5.6	5.1	7.7	5.3	5.7	11.9	4.8	5.7	12.3
CT only	3.4	3.8	5.4	5.6	5.5	8.1	5.8	6.2	15.3	3.7	5.1	12.0
Twist+CT	3.4	3.8	5.2	5.6	5.5	8.6	5.8	6.2	15.9	3.6	5.1	12.3

DFT calculations, reflecting both a charge transfer and twist of the molecule. The parameters reported include the position of the first peak of the RDF ($r_{g_{\max}}$), the mean radius of the first solvation shell

$$\langle r \rangle = \frac{\int_0^{r_s} r\, 4\pi r^2 \rho_0\, g(r)\, \mathrm{d}r}{\int_0^{r_s} 4\pi r^2 \rho_0\, g(r)\, \mathrm{d}r} = \frac{\int_0^{r_s} r^3 g(r)\, \mathrm{d}r}{\int_0^{r_s} r^2 g(r)\, \mathrm{d}r}, \tag{15}$$

and the cumulative coordination number

$$CN(r_s) = 4\pi\rho_0 \int_0^{r_s} r^2 g(r)\, dr, \tag{16}$$

where ρ_0 is the isotropic solvent density and r_s is the distance representing the extent of the first solvation shell (vertical lines in Suppl. Fig. 30).

In the ground state, the acetonitrile molecules surrounding the thioindigo group show a preferential coordination to the oxygen of the carbonyl bond with the methyl site, indicated by a structured peak in the O(HTI-J)-Me(acetonitrile) RDF at around 3.5 Å, while the O(HTI-J)-N(acetonitrile) RDF appears very broad, with a small peak around 5.6 Å. This is also apparent from a plot of the ratio between the cumulative coordination numbers of nitrogen and methyl sites of acetonitrile computed from the RDFs of O(HTI-J)-N(acetonitrile) and O(HTI-J)-Me(acetonitrile) distances, as shown in Suppl. Fig. 31. In the excited state, the coordination of the methyl sites of the acetonitrile molecules to the oxygen atom of HTI-J becomes stronger, as indicated by an increased height of the O(HTI-J)-Me(acetonitrile) RDF peak corresponding to an increase of the cumulative coordination number of $\sim$ 1.5. The O(HTI-J)-N(acetonitrile) RDF also becomes more structured, with a clear peak emerging at around 5.6 Å, $\sim$2 Å from the peak of the O(HTI-J)-N(acetonitrile), which is close to the Me-N distance of acetonitrile. Overall, the ratio of coordinating methyl sites over N sites becomes bigger, as shown in Suppl. Fig. 31. The stronger coordination of the solvent to the carbonyl of the HTI-J molecule is due to a shift of electronic charge from the julolidine to the thioindigo group in the excited state.

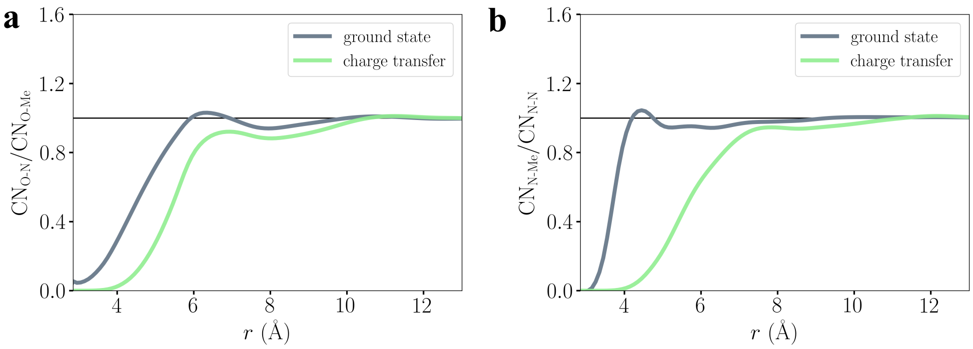

Suppl. Fig. 31 Ratio of the cumulative coordination numbers for the nitrogen ($CN_{\mathrm{N(ACN)}}$) and the carbon methyl ($CN_{\mathrm{Me(ACN)}}$) sites of acetonitrile as a function of the distance from (a) the oxygen and (b) the nitrogen of HTI-J. The cumulative coordination number is computed from radial distribution functions of equilibrium molecular dynamics simulations of the HTI-J in acetonitrile. The cumulative coordination number ratios are shown for simulations of the HTI-J in the ground state and in the excited state modelled by changing only the partial charges compared to the ground state.

The solvation shell rearrangement is even larger around the julolidine part of the molecule. There, the N(HTI-J)-N(acetonitrile) and N(HTI-J)-Me(acetonitrile) RDFs for the ground state are very broad without a clear dominant peak. Therefore, the solvation shell around the julolidine appears highly unstructured in the ground state. In the excited state, a clear peak emerges around 3.7 Å in the N(HTI-J)-N(acetonitrile) RDF and a broader peak around 5.8 Å in the N(HTI-J)-Me(acetonitrile) RDF. The ratio between the cumulative coordination numbers of the N(HTI-J)-Me(acetonitrile) and N(HTI-J)-N(acetonitrile) RDFs shows a dominant preferential orientation with the N sites up to a distance of $\sim$ 12 Å (see Suppl. Fig. 31). The structuring of the solvation shell and increased coordination with the N sites of acetonitrile reflect a loss of negative charge on the julolidine group in the excited state.

When the excited state is modelled by changing both the partial charges and the molecular structure very similar changes in the solute-solvent RDFs are obtained, demonstrating that the rearrangement of the solvation shell is driven by charge transfer rather than an intramolecular structural change (see also Figure 6 in the main text).

Suppl. Fig. 32 shows the difference scattering signals calculated from equilibrium MD simulations for the ground state and for three different models for the excited state. In the first model ("Twist and CT"), both partial charges and geometry are changed in the excited state simulations compared to the ground state, based on the orbital-optimized density functional excited state calculations presented in the previous sections. In the second model ("CT only"), only the partial charges are changed, and the ground state geometry is retained. Finally, in the third model ("Twist only"), only the geometry is changed, retaining the partial charges of the ground state. The terms calculated from the intra-solute RDFs (in orange) and the solute-solvent RDFs (in blue) are shown separately, as as well as their sum (in black). For the "Twist and CT" model, the solvent-solvent signal dominates at $q < 3.5$ Å^{-1}, while the intra-solute dominates at higher q.

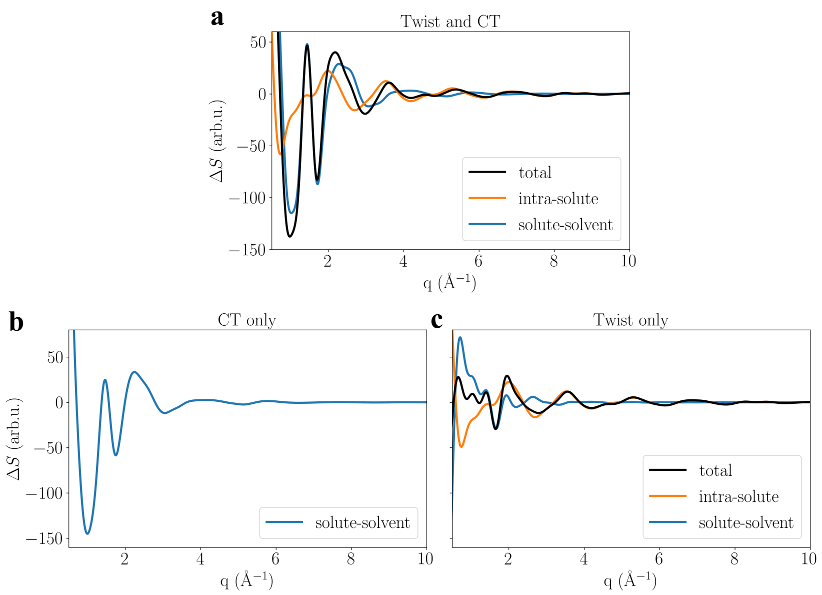

Suppl. Fig. 32 Difference scattering curves calculated from the equilibrium molecular dynamics simulations for the three different models representing the excited state; **a** intramolecular charge transfer and twisting (changing both partial charges and geometry compared to the ground state), **b** only charge transfer (changing only partial charges), and **c** only twist (changing only geometry). The scattering signals calculated from intra-solute (orange curve) and solute-solvent (blue curve) radial distribution functions are shown separately, as well as the total scattering signal that is the sum of these contributions (black curve).

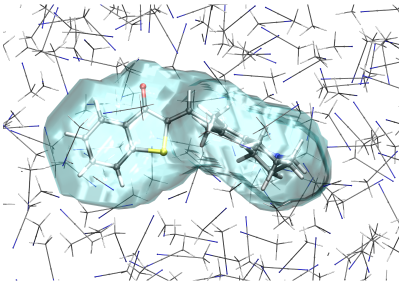

Suppl. Fig. 33 The cyan isosurface shows the surface around the HTI-J where the number density of the solvent is 0.01 $Å^{-3}$, as obtained from the molecular dynamics simulations with a charge transfer and twist model of the excited state. The excluded volume is calculated as the volume of the region enclosed by this surface (see Suppl. Table 8).

To quantify changes upon excitation in the volume around the HTI-J excluded to the solvent, the number density n of the solvent atoms, averaged over all the frames in the MD trajectory, was calculated for the ground state and the three different models used for the excited state. We define the excluded volume as the volume of the region around the solute where $n < 0.01$ $Å^{-3}$, corresponding to less than 1 % probability

Suppl. Table 8 Excluded volume, calculated from the equilibrium molecular dynamics simulations as the volume of the region around the HTI-J where the number density of the solvent atoms is less than 0.01 $Å^{-3}$. The values are shown for the ground state and the three models for the excited state.

	Excluded volume ($Å^3$)
GS	415
Twist only	415
CT only	403
Twist and CT	404

of finding a solvent atom. The surface corresponding to $n = 0.01$ $Å^{-3}$ for the twist and charge transfer model of the excited states of HTI-J is shown in Suppl. Fig. 33. The excluded volume is obtained as the volume of the region enclosed by this surface. As table 8 shows, twisting of the HTI-J has a minimal effect on the excluded volume, whereas charge transfer causes the excluded volume to decrease. This is attributed to the rearrangement of the polar acetonitrile molecules as a response of the thioindigo and julolidine fragments HTI-J getting more positively and negatively charged, respectively, causing the solvent molecules to get closer to the solute on average.

10.2 Suppl. Note 10.2. Nonequilibrium molecular dynamics simulations

Suppl. Fig. 34 shows the time evolution of the kinetic energy of the HTI-J molecule and the acetonitrile solvent molecules obtained in the nonequilibrium MD simulations of vibrational cooling and heat transfer to the solvent. In the simulations, an energy of 2.64 eV, corresponding to the photon energy of the laser pump pulse, is instantaneously deposited as vibrational kinetic energy in the solute in the ground electronic state. Initially, roughly half of the total excess energy is rapidly transformed into potential energy of the system. The other half leads to a corresponding increase in the kinetic energy of the solvent. A biexponential fit to the time-dependent kinetic energy of the solute after the rapid conversion into potential energy gives two time constants, 1.80 ± 0.02 ps and 12.18 ± 0.01 ps. A biexponential fit of the time-dependent solvent kinetic energy gives time constants of 2.95 ± 0.04 ps and 14.17 ± 0.05 ps.

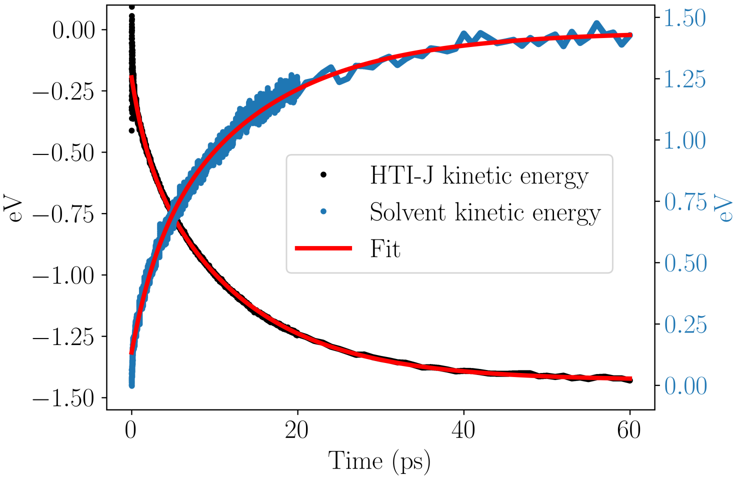

Suppl. Fig. 34 Time evolution of the kinetic energy of HTI-J (black dots) and the acetonitrile solvent molecules (blues dots) obtained in nonequilibrium molecular dynamics simulations after a 2.64 eV instantaneous vibrational excitation of HTI-J in the ground state. The red lines represent best-fitting curves of a biexponential fit.

Supplementary references

[1] Li, T.-y., Muthiah Ravinson, D. S., Haiges, R., Djurovich, P. I. & Thompson, M. E. Enhancement of the luminescent efficiency in carbene-au(i)-aryl complexes by the restriction of renner–teller distortion and bond rotation. *Journal of the American Chemical Society* **142**, 6158–6172 (2020).

[2] Dance, Z. E. X. *et al.* Direct observation of the preference of hole transfer over electron transfer for radical ion pair recombination in donor-bridge-acceptor molecules. *Journal of the American Chemical Society* **130**, 830–832 (2008).

[3] Wiedbrauk, S. *et al.* Twisted hemithioindigo photoswitches: solvent polarity determines the type of light-induced rotations. *Journal of the American Chemical Society* **138**, 12219–12227 (2016).

[4] Zweig, J. E. & Newhouse, T. R. Isomer-specific hydrogen bonding as a design principle for bidirectionally quantitative and redshifted hemithioindigo photoswitches. *Journal of the American Chemical Society* **139**, 10956–10959 (2017).

[5] Müller, C., Pascher, T., Eriksson, A., Chabera, P. & Uhlig, J. KiMoPack: A python package for kinetic modeling of the chemical mechanism. *The Journal of Physical Chemistry A* **126**, 4087–4099 (2022).

[6] Ekvall, K. *et al.* Cross phase modulation artifact in liquid phase transient absorption spectroscopy. *Journal of Applied Physics* **87**, 2340–2352 (2000).

[7] Lorenc, M. *et al.* Artifacts in femtosecond transient absorption spectroscopy. *Applied Physics B: Lasers and Optics* **74**, 19–27 (2002).

[8] Ashiotis, G. *et al.* The fast azimuthal integration python library: pyfai. *Journal of Applied Crystallography* **48**, 510–519 (2015).

[9] Brandt van Driel, T. *et al.* Disentangling detector data in XFEL studies of temporally resolved solution state chemistry. *Faraday discussions* **177**, 443–65 (2015).

[10] Haubro, M. L. *et al.* Analysis strategies for time-resolved x-ray solution scattering at high repetition rate xfel sources. *Journal of Synchrotron Radiation* **33**, 1–14 (2026).

[11] Harmand, M. *et al.* Achieving few-femtosecond time-sorting at hard x-ray free-electron lasers. *Nature Photonics* **7**, 215–218 (2013).

[12] Biasin, E. *et al.* Anisotropy enhanced x-ray scattering from solvated transition metal complexes. *Journal of Synchrotron Radiation* **25**, 306–315 (2018).

[13] Lorenz, U., Møller, K. B. & Henriksen, N. E. On the interpretation of time-resolved anisotropic diffraction patterns. *New Journal of Physics* **12**, 113022 (2010).

[14] Ki, H. *et al.* Optical kerr effect of liquid acetonitrile probed by femtosecond time-resolved x-ray liquidography. *Journal of the American Chemical Society* **143**, 14261–14273 (2021).

[15] Haldrup, K., Christensen, M. & Nielsen, M. M. Analysis of time-resolved x-ray scattering data from solution-state systems. *Acta Crystallographica Section A* **66**, 261–269 (2010).

[16] Hendler, R. W. & Shrager, R. I. Deconvolutions based on singular value decomposition and the pseudoinverse: a guide for beginners. *Journal of Biochemical and Biophysical Methods* **28**, 1–33 (1994).

[17] Montoya-Castillo, A. *et al.* Optically induced anisotropy in time-resolved scattering: Imaging molecular-scale structure and dynamics in disordered media with experiment and theory. *Phys. Rev. Lett.* **129**, 056001 (2022).

[18] Haynes, W. M. (ed.) *CRC Handbook of Chemistry and Physics* 95 edn (CRC Press, 2014).

[19] Kjær, K. S. *et al.* Introducing a standard method for experimental determination of the solvent response in laser pump, x-ray probe time-resolved wide-angle x-ray scattering experiments on systems in solution. *Physical Chemistry Chemical Physics* **15**, 15003–15016 (2013).

[20] Debye, P. Zerstreuung von röntgenstrahlen. *Annalen der Physik* **351**, 809–823 (1915).

[21] Haldrup, K. Singular value decomposition as a tool for background corrections in time-resolved xfel scattering data. *Philosophical Transactions of the Royal Society B: Biological Sciences* **369** (2014).

[22] Dent, A. J., Stephenson, P. C. & Greaves, G. N. The extraction of signal to noise values in x-ray absorption spectroscopy. *Review of Scientific Instruments* **63**, 856–858 (1992).

[23] Stallhofer, K. *et al.* Electronic and geometric characterization of tict formation in hemithioindigo photoswitches by picosecond infrared spectroscopy. *The Journal of Physical Chemistry A* **125**, 4390–4400 (2021).

[24] Nguyen, S. C., Lomont, J. P., Caplins, B. W. & Harris, C. B. Studying the dynamics of photochemical reactions via ultrafast time-resolved infrared spectroscopy of the local solvent. *Journal of Physical Chemistry Letters* **5**, 2974–2978 (2014).

[25] Zhang, Y., Chen, J. & Kohler, B. Hydrogen bond donors accelerate vibrational cooling of hot purine derivatives in heavy water. *Journal of Physical Chemistry A* **117**, 6771–6780 (2013).

[26] Middleton, C. T., Cohen, B. & Kohler, B. Solvent and solvent isotope effects on the vibrational cooling dynamics of a dna base derivative. *Journal of Physical Chemistry A* **111**, 10460–10467 (2007).

[27] Ruckebusch, C., Sliwa, M., Pernot, P., de Juan, A. & Tauler, R. Comprehensive data analysis of femtosecond transient absorption spectra: A review. *Journal of Photochemistry and Photobiology C: Photochemistry Reviews* **13**, 1–27 (2012).

[28] Nakamura, M., Chubachi, K., Tamura, K. & Murakami, S. Thermodynamic properties of [xHCON(CH3)2 or CH3CN + (1-x)(ch3)2so] at the temperature 298.15 k. *The Journal of Chemical Thermodynamics* **25**, 1311–1318 (1993).

[29] Katayama, T. *et al.* Atomic-scale observation of solvent reorganization influencing photoinduced structural dynamics in a copper complex photosensitizer. *Chemical Science* **14**, 2572–2584 (2023).

[30] Haldrup, K. *et al.* Ultrafast X-Ray Scattering Measurements of Coherent Structural Dynamics on the Ground-State Potential Energy Surface of a Diplatinum Molecule. *Physical Review Letters* **122**, 63001 (2019).

[31] Ngan, V. T., Gopakumar, G., Hue, T. T. & Nguyen, M. T. The triplet state of indigo: Electronic structure calculations. *Chemical Physics Letters* **449**, 11–17 (2007).

[32] Paetow, L. & Neugebauer, J. Excited state dipole moments from ΔSCF: a benchmark. *Physical Chemistry Chemical Physics* (2025).

[33] Selenius, E., Sigurdarson, A. E., Schmerwitz, Y. L. & Levi, G. Orbital-optimized versus time-dependent density functional calculations of intramolecular charge transfer excited states. *Journal of Chemical Theory and Computation* **20**, 3809–3822 (2024).

[34] Ziegler, T., Rauk, A. & Baerends, E. J. On the calculation of multiplet energies by the hartree-fock-slater method. *Theor. Chim. Acta* **43**, 261–271 (1977).

[35] Breneman, C. M. & Wiberg, K. B. Determining atom-centered monopoles from molecular electrostatic potentials. the need for high sampling density in formamide conformational analysis. *Journal of Computational Chemistry* **11**, 361–373 (1990).

[36] Riplinger, C. & Neese, F. An efficient and near linear scaling pair natural orbital based local coupled cluster method. *The Journal of Chemical Physics* **138**, 034106 (2013).

[37] Riplinger, C., Sandhoefer, B., Hansen, A. & Neese, F. Natural triple excitations in local coupled cluster calculations with pair natural orbitals. *The Journal of chemical physics* **139** (2013).

[38] Duchstein, P., Neiss, C., Görling, A. & Zahn, D. Molecular mechanics modeling of azobenzene-based photoswitches. *Journal of molecular modeling* **18**, 2479–2482 (2012).

[39] Dunning Jr, T. H. Gaussian basis sets for use in correlated molecular calculations. i. the atoms boron through neon and hydrogen. *The Journal of chemical physics* **90**, 1007–1023 (1989).

[40] Woon, D. E. & Dunning Jr, T. H. Gaussian basis sets for use in correlated molecular calculations. iii. the atoms aluminum through argon. *The Journal of chemical physics* **98**, 1358–1371 (1993).

[41] Garcia-Ratés, M., Becker, U. & Neese, F. Implicit solvation in domain based pair natural orbital coupled cluster (dlpno-ccsd) theory. *Journal of Computational Chemistry* **42**, 1959–1973 (2021).
