# Supplementary material for: Tracking polar solvation dynamics of a photoexcited organic chromophore with ultrafast X-ray scattering
Source: Nat Commun. 2026 Apr 13;17:5080. doi: 10.1038/s41467-026-71635-1 (PMC13247046; doi:10.1038/s41467-026-71635-1)
Supplement: Supplementary file 2 — Transparent Peer Review file [file 41467_2026_71635_MOESM2_ESM.pdf]

nature portfolio

Peer Review File

Tracking Polar Solvation Dynamics of a Photoexcited Organic Chromophore with Ultrafast X-ray Scattering

Corresponding Author: Professor Gianluca Levi

This file contains all reviewer reports in order by version, followed by all author rebuttals in order by version.

Attachments originally included by the reviewers as part of their assessment can be found at the end of this file.

Version 0:

Reviewer comments:

Reviewer #1

(Remarks to the Author)
Summary
This manuscript reports a femtosecond time-resolved X-ray solution scattering (TR-XSS) study of hemithioindigo–julolidine (HTI-J) in acetonitrile, aiming to visualize charge-transfer-induced solvation dynamics at the atomic scale. By constructing a global fitting model that includes optical Kerr effect (OKE), vibrational relaxation (VR), solvation-shell rearrangement (SSR), and bulk heating (ΔT) components, the authors attempt to separate and interpret multiple solvent responses over sub- to tens-of-picosecond timescales. The work represents a meaningful attempt to extend TR-XSS to organic chromophores containing only light atoms and highlights the potential of model-based decomposition for disentangling complex solvent and solute responses.

Overall assessment
The work tackles an important and technically demanding problem in ultrafast structural chemistry: distinguishing charge-transfer-driven solvent reorganization from solute structural motion in a light-atom system. The experimental setup and analysis framework are sophisticated, and the topic is timely. However, several central claims and figures appear to overstate what the data can conclusively demonstrate, which can mislead the readers. The following points must be thoroughly addressed before the manuscript can be considered for publication.

1) On the generalization and claim of "atomic-scale observation"
The title and abstract emphasize "*atomic-scale observation of polar solvation dynamics*," yet the analysis does not yield explicit atomic coordinates. The observed effects are primarily inferred from solvent density changes in the scattering profiles induced by charge transfer in the solute molecules, whose atomic-scale structural changes (twisting) are themselves not directly visible in their scattering signal. This phrasing may overstate what was actually observed with the technique. The authors should clarify that the study provides indirect structural sensitivity to solvent reorganization rather than a direct atomic reconstruction. A more accurate framing in the title or abstract, focusing on charge-transfer-driven solvent reorganization, would strengthen the manuscript's credibility.

2) On CT-only versus CT + twist models
Figures 4–5 and Supplementary Figs. S19–S21 show that including a twist marginally improves the fit near 2–3 $Å^{-1}$ but worsens agreement around 1 $Å^{-1}$. However, the manuscript does not present quantitative criteria for assessing these differences. It would be helpful to include direct numerical comparisons (e.g., χ^2 values) between the two models to support the argument. If the signal-to-noise ratio prevents discrimination, the authors should state explicitly that the current data cannot resolve solute geometry changes. Displaying both fits side by side in a single panel would make this limitation visually clear.

3) On the definition and interpretation of the "SSR" component
The term "*solute and solvation-shell structural rearrangement (SSR)*" suggests resolvable solute motion, yet the fits use time-invariant shape functions and provide no quantitative evidence of twisting. Because the observed features can be explained by charge redistribution alone, the terminology should be revised to "solvation-shell rearrangement" to avoid implying direct

solute structural change. Related schematics (e.g., Fig. 5c, 5f) should also be modified so that solute twisting is presented as hypothetical rather than confirmed via TR-XSS. The text should clearly state that the experimental data primarily support solvent reorganization following charge transfer.

4) On figure presentation and potential misinterpretation
Some figures (notably Fig. 2b and Fig. 5f) depict a twisted geometry that may be interpreted as an experimentally observed structure. These should be redrawn to emphasize charge-transfer-driven polarization and solvent motion instead of definite twisting of the solute molecule. Figure captions should explicitly describe such illustrations as conceptual representations based on DFT calculations and transient absorption measurement, not experimental determinations by TR-XSS.

5) On the DFT assignment and relevance of the proposed twisting geometry
The proposed twisting is supported by DFT calculations on a julolidine-substituted HTI derivative. However, static DFT geometries may not accurately reflect solvated structures under experimental conditions. The authors should justify how well the computed geometries correspond to the actual solute configuration in acetonitrile and clarify whether solvent effects were included in the calculations. It is recommended that the authors explore how the twisting geometry and the extent of charge transfer depend on a broader range of quantum chemical methods.

6) On excitation fluence and possible nonlinear effects
The excitation conditions correspond to a pulse energy of 12 μJ at 470 nm focused to a 73 × 95 μm (FWHM) spot, which gives an average fluence of approximately 1.1 mJ mm^{-2} within the FWHM area, assuming that half of the pulse energy lies inside the Gaussian FWHM circle. This fluence is relatively high for pump–probe experiments in solution and may enter the regime where multiphoton ionization or solvent plasma formation becomes relevant. Under such conditions, the number of solvated electrons and ionized solvent molecules can approach significant concentrations, potentially modifying the solvent response and introducing additional anisotropy or background signals in the transient scattering data.
It would therefore be important for the authors to demonstrate that the TR-XSS signals scale linearly with excitation fluence and that the measurements were performed under single-photon excitation conditions. Power-titration tests or transient absorption data verifying linear excited-state population would provide valuable support.

7) On extraction of the heating contribution
The heating term is obtained by averaging the 55–60 ps trace under the assumption that non-thermal contributions have fully decayed. While this is reasonable given the reported 2.6 ps electronic lifetime, the robustness of this assumption should be demonstrated. Testing alternative time windows (e.g., 45–50 ps versus 55–60 ps) and reporting how the resulting heating profile varies would provide useful validation. Any residual non-thermal contribution could also be estimated from the model or transient absorption data and summarized in the Supplementary Information.

8) On the treatment of solvent effects in DFT calculations
The DFT calculations appear to be based on static geometries optimized within an implicit solvent model (CPCM). Such treatment may not adequately capture the specific solute–solvent interactions that influence the charge distribution and dipole moment in the charge-transfer state. To ensure consistency between theory and experiment, the authors should perform additional excited-state calculations that include explicit solvation, for example using a QM/MM approach, to evaluate whether the computed charge distribution and dipole moments remain consistent with the experimental data. This additional analysis would help determine whether the experimentally observed solvent response arises solely from electronic polarization of the solvent environment or also involves genuine structural rearrangements within the first solvation shell that are not represented in the implicit-solvent model.

9) On the anisotropic signal and its physical interpretation
The Supplementary Information states that the anisotropic signal contains the response to the optical Kerr effect (OKE), and that this fast OKE component is used to define time zero and determine the time resolution. However, the anisotropic signal can also contain valuable information about the orientational dynamics of the photoexcited solute and the correlated motion of nearby solvent molecules. A more complete analysis of the anisotropic component would provide independent insight into the solvation mechanism. In particular, the authors should clarify whether contributions from the photoexcited solute or its first solvation shell are distinguishable from the pure OKE response of the bulk solvent. Demonstrating this separation, even qualitatively, would substantially reinforce the interpretation of solvent reorganization dynamics.

Minor comments
- Terminology: The term *solute–solute* is used throughout the manuscript (e.g., Fig. 7) to denote intramolecular scattering within a single solute molecule. However, this usage is unconventional and potentially misleading, because solute–solute typically refers to interference terms arising from scattering between different solute molecules in highly concentrated solutions. The authors should rectify this usage by adopting a clearer label such as solute term or intra-solute scattering.
- Experimental conditions: Include a compact table summarizing concentration, fluence, jet thickness, and pump–probe geometry.
- Captions and legends: Ensure that all figures specify whether heating and OKE contributions are included or subtracted.

Conclusion and recommendation
This manuscript presents an important step toward extending TR-XSS to light-atom organic chromophores and toward dissecting solvent reorganization following charge transfer. The experimental results are strong, and the analytical framework is promising. However, the current data do not provide clear evidence of solute twisting, and several textual and visual elements do not clearly convey this point. Clarifying terminology, presenting CT-only versus CT + twist comparisons

quantitatively, and harmonizing figure representations would substantially improve the clarity and rigor of the paper.

Recommendation: Major revision.

(Remarks on code availability)

Reviewer #2

(Remarks to the Author)
Please find a pdf of my review attached. Thank you.

(Remarks on code availability)
Not applicable

Reviewer #3

(Remarks to the Author)
The manuscript presents an in-depth study of a nonequilibrium response of a polar solvent acetonitrile which is caused by charge transfer in the small organic molecule hemithioindigo (HTI) chromophore following it's photoexcitation by short femtosecond laser pulse. The work consists of experiments in static and time-resolved pump-probe absorption (VIS) spectroscopy, time-resolved pump-probe X-ray solution scattering (Swiss XFEL) and extensive computational experiments, namely density functional theory (DFT) and molecular dynamics (MD) simulations to probe the structural and electronic changes in HTI and the subsequent response of acetonitrile polar solvent. The work utilizes advanced and robust techniques for data processing, data analysis and statistical analysis of the data. The results presented in this manuscript are of interest to scientists working in the fields of chemistry, photonics, photobiology and solvation dynamics.
Significance.
Solution X-ray scattering is a contrast X-ray imaging technique in which measured X-ray scattering signal is proportional to difference of densities (contrast) of solute and solvent. Therefore, for small organic molecules, such as HTI chromophore, it is notoriously challenging to disentangle scattering from the solute molecule and its solvent, in this case acetonitrile. Time-Resolved X-ray Solution Scattering (TRXSS) has capability to probe simultaneously structural properties of a solute molecule and solvent molecules, and their interaction after the reaction initiation, in this case photoinitiation of the solute chromophore. The authors studied how photoexcitation of the HTI chromophore changes distribution of its intramolecular charges and its structural geometry, and how acetonitrile solvent responds to the changes. The charge transfer properties of HTI were amplified by substituting stilbene fragments by julolidine group (HTI-J) which caused increase of dipole moment of the molecule upon photoactivation from 5.3 D in ground state to 27.3 D in the excited state (DFT calculations) and therefore amplified electrostatic interaction of polar solvent with the photoexcited molecule of HTI-J.
The authors observed experimentally, analyzed and determined the sequence of the processes which occur in the system upon HTI-J photoexcitation: polarization of the polar solvent by the electric field of the pump laser light (optical Kerr effect), changing energy of HTI-J from the ground to exited state and charge redistribution in the solute, vibrational energy transfer from solute to a solvent, solvent response and electrostatic interaction of the solute molecule and the polar solvent. While changing structure of HTI-J upon photoexcitation does not contribute significantly to scattering patterns observed in the experiment, vibrational energy exchange and electrostatic interaction between the exited state of HTI-J and polar solvent does. The significance of this work is in observing structural changes in solute and solvent, encoded in the scattering patterns, identifying the nature of chemical processes governing photoexcitation of HTI-J and its energy exchange with polar solvent and capturing time scales and sequences of those processes. This work contributes significantly to understanding mechanisms of solvation dynamics of polar solvents.
Novelty and originality.
The novelty of this work is in observing experimentally a change is the scattering pattern due to the dipolar rearrangement of the solvent molecules surrounding photoexcited HTI-J, capturing the formation of the transient solvation shell structure on a ~0.5 ps timescale and its re-equilibration as the system returns to the ground state with a time constant of ~2.6 ps, This is the first time when such structural dipolar rearrangement of the solvent molecules following HTI-J photoexcitation is experimentally captured in X-ray solution scattering patterns and those experimentally observed structural changes are modeled on the atomic scale.
Data and methodology. Validity of the results.
The manuscript presents state of the art research both experimentally, methodologically and computationally. It combines cutting edge technologies in TRXSS with static and time-resolved optical spectroscopy and extensive in-silico experiments. The combination of all those tools is required to identify, assign and model physical processes which occur upon photoexcitation of a small organic chromophore in a polar solute including energy dissipation, relaxation and charge re-distribution processes. The disentanglement of those processes is accomplished by a robust stepwise global optimization procedure guided by methods of singular vector decomposition, DFT and MD simulations. Methods section in the manuscript, and Supplemental Material present in detail robustness of procedures of statistical analysis and errors handling, data acquisition, data processing, data analysis and simulations and ensure validity of the results.
Comments and Suggestions.
-Data presentation should be improved. The time-resolved scattering patterns are dominated by the solute heating signal caused by vibrational cooling of solute molecules and heating of the solvent molecules. It is very important to see how data looks after global fitting and subtraction of the solvent heating signal. In this regard map representation does not give much information about actual data and noise levels contained in the data. In addition to the maps (Figure 4) heating signal

subtracted data should be presented for selected time delays where a major contribution from specific processes is identified. The data should show experimental errors, and the quality of the fits, discussed in the manuscript.
- It is not clear why authors use acetonitrile patten from a ref [15] in the data analysis. The experimental data were normalized according to established standards. 1 HTI-J molecule is surrounded by 6400 acetonitrile molecules (Supplemental Materials, Liquid unit cell scaling) and in the fitting procedure of scattering pattern from [15] authors dismiss very small contribution for HTI-J molecule anyway. Why authors need fitting of the acetonitrile data, collected at different experimental conditions previously, and then rescale already normalized data. Did authors try to process the data without this additional scaling? Did they perform static measurements of the acetonitrile and temperature jump measurements in this solvent along with HTI-J photoexcitation measurements?
-The authors did laser power titration measurements for the laser wavelength 470nm and proved that the measurements are performed in the linear regime. How many photons were delivered per HTI-J chromophore? Did authors try to estimate what additional fitting procedure (discussed above) could contribute to errors in the heating signal increase (temperature jump) and authors conclusions about multiphoton excitation in the experiment ?
- Multiphoton absorption in organic chromophores may significantly impact energy transfer and relaxation processes and trigger different pathways and rates. Therefore, it may alter the photophysical properties of the system. The authors should discuss in the main text what changes (if any) in the identified in the manuscript relaxation processes this may cause besides mentioned in the manuscript fast non-radiative relaxation from higher excited state causing fast solvent heating (0.2ps, Table1).

-DFT calculations and MD simulations are very important for the atomic-scale interpretation of the data . How MD simulations are used for the determination of the final structures, however, requires additional explanations. The manuscript presents exiting results of interplay between molecule of solute and surrounding molecules of solvent (solvation dynamics), including solvent excluded volume changes, changes in its interatomic distances and changes in polarization of the solvent. In Methods it is emphasized that for equilibrium MD simulations, 400000 equidistant frames were collected over 400 ns. It is not clear how these 400000 trajectories were used in the final selection of the structures. How many runs were performed, what were criteria for the final structure selections? This should be explained, and appropriate data should be presented.
- It is confusing to read sections of the main text of the manuscript describing solvent heating, vibrational relaxation, and optical Kerr effect and solvation dynamics with the detailed explanations of physics, energy flow and timing of multiple processes. It would be much easier for a reader if this information is also presented in a visual schematic or a diagram which a reader can follow in addition to the text description.
-Sentence lines 95-98 is not clear, it read like" ..Knowledge … could reduce energy loss through thermal dissipation..', perhaps it should be rephrased.
References:
The manuscript references previous literature adequately.
Clarity:
The manuscript is written in clear language, understandable to an expert and a general audience. Argumentation is clear with the appropriate references when required. Abstract, Introduction and Conclusions are appropriate. Methods and Supplemental materials are very helpful.
Reviewer expertise: My expertise is in time-resolved X-ray solution scattering and supporting experiments (for example optical spectroscopy) and analysis of time-resolved and static X-ray solution scattering data, in particular for biomolecules in solutions. The topic of this manuscript does align partially with the field of my expertise.
My assessment that the manuscript can be published in Nature Communications with revisions, which address above comments

(Remarks on code availability)
I reviewed the code but did not run it. The code provides step-by step guide for data analysis of TRXSS and TRA. Fits clearly show good quality. The code is usable resource for the community and nice training tool for those who start their career in the field. README file provides lists of tutorials on how to run the code. It would be helpful if authors also provided Conda environment (instructions) as running the code requires multiple python-based packages installed.

Reviewer #4

(Remarks to the Author)
This is an important experimental advance – obtaining time-resolved solution scattering on an organic molecule. Time-resolved X-ray solution scattering on the femto-picosecond timescale is now a well-established technique at X-FELs. To date, these experiments have been performed on solute-solvent systems where the solute contains high Z atom(s), which have strong scattering cross-sections.
Before the work is published the authors should address the following comments:
1. The issue of high optical pump fluence 12 microJ/pulse needs to be addressed. From Figure S8, this reviewer does not agree that the data was collected in the linear regime. The data could be easily fit by higher order terms.
a. What was the optical pump fluence used in the optical transient absorption experiments? If different from the X-ray experiments, then it should be rationalized, or the TA experiments must be performed with the same fluence for a 1:1 comparison.
b. The pump fluence dependent study for the transient X-ray was conducted at 20 ps. At that time delay, there is very little difference signal from the solute. It seems that the pump-fluence is measuring the solvent heating response. Is that correct?
c. At some point the in the main text of SI, the authors point to multi-photon effects in the solvent. How does that match with the difference signal being linear in pump-fluence.

2. The authors have detailed their data analysis and data reduction. However, one is left wondering if the data is over-fit. The multiple timescales and nomenclature used in Table 1 of the main text are confusing. It is also odd that the transient absorption data does not measure the longer timescales for solute vibrational cooling or the slow solvent heating. Why are these not the same thing?
3. The authors claim that their work provides atomic scale resolution of polar solvation dynamics. However, they do not provide any non-equilibrium RDFs of the solute-solvent interactions via MD simulations. Their MD simulations should include non-equilibrium solute-solvent interactions because they fit these timescales in their data. If non-equilibrium MD simulations are not included, then there should be a discussion in the main text about what is missing.

In conclusion, while this is an important experimental demonstration, the data analysis and quality of the data do not provide any new physically relevant information.

(Remarks on code availability)

Version 1:

Reviewer comments:

Reviewer #1

(Remarks to the Author)
Summary and Recommendation
The authors have submitted a substantially revised manuscript that successfully addresses the critical technical and interpretational concerns raised in the first round of review. The revision demonstrates a high degree of transparency, particularly regarding the excitation fluence conditions and the limitations in distinguishing solute structural models, and incorporates rigorous theoretical validation (DLPNO-CCSD(T)). The authors have adequately addressed the comments in their point-by-point responses, and the corresponding changes to the manuscript are appropriate. The work now stands as a robust contribution to the field. I recommend acceptance of the manuscript, subject to one final addition described below to fully substantiate the “atomic-scale” claims made in the title and abstract.

1) Request for quantitative analysis of solvation shell (final action item)
While the authors have clarified in the text that the experiment tracks the solvation-shell rearrangement signal rather than specific atomic trajectories, the support for “atomic-scale observation” currently relies on qualitative comparisons of MD-derived Radial Distribution Functions (RDFs) in Figure 6. To bridge the gap between the qualitative visual evidence and the quantitative claim of “atomic-scale” sensitivity, a specific breakdown of the structural changes is necessary.

Suggestion:
I suggest including a table (in the Main Text or SI) summarizing the quantitative structural parameters extracted from the MD simulations for the first and second solvation shells. Specifically:
- Peak positions (r_{max}) for relevant atom pairs (e.g., Solute-N, Solute-Methyl) in the ground state vs. charge-transfer state.
- Coordination numbers (CN) for these shells.
- The quantitative magnitude of the change (Δr, ΔCN) upon excitation.
Providing these numbers will concretely define what “atomic-scale” changes are responsible for the observed scattering signal.

2) Assessment of Resolved Issues
All other comments from the previous round have been satisfactorily addressed:
- Model Comparison (Point 2): The new side-by-side comparison (Fig. 7) and the admission that the models are statistically indistinguishable ($\chi^2 \approx 4$) are appropriate and scientifically rigorous.
- Terminology (Point 3): The adoption of “Solvation-Shell Rearrangement (SSR)” eliminates ambiguity.
- Figures (Point 4): Revised figures and captions correctly attribute twisted geometries to theoretical predictions rather than direct observation.
- Theoretical Validation (Points 5, 8): The inclusion of DLPNO-CCSD(T) calculations and clarification of the explicit solvent MD workflow provide a solid theoretical foundation.
- Fluence/Heating (Points 6, 7): The power titration analysis and the validation of the heating signal extraction by comparing different time delay windows (Fig. S17) effectively resolve concerns about signal contamination.
- Anisotropic Signal (Point 9): The SVD analysis added to the SI adequately addresses the potential of the anisotropic data.
- Literature Context: The authors have appropriately cited a key reference, providing the necessary context on anisotropic TR-XSS and atomic charge redistribution.

Recommendation: Accept (after minor revision regarding the RDF quantification).

(Remarks on code availability)

Reviewer #2

(Remarks to the Author)
I have read carefully the response of the authors to the referee reports, which all four raise interesting questions and comments. The authors' responses, in some instances including additional work, and finally the commensurate changes in the manuscript (or the SI), are satisfactory and improve the manuscript further. My overall assessment is that the resulting manuscript warrants publication in Nature Communications.

(Remarks on code availability)

Reviewer #3

(Remarks to the Author)
My comments were addressed in the revisions of the manuscript NCOMMS-25-77889A "Atomic-Scale Observation of Polar Solvation Dynamics Triggered by Photoinduced Charge Transfer in an Organic Chromophore". My assessment is that the revised version of the manuscript can be published in Nature Communications.

(Remarks on code availability)

Reviewer #4

(Remarks to the Author)
This review is on the revised manuscript and response to reviewer comments of Levi et al on the ultrafast X-ray solution scattering (XSS) study of an organic chromophore in solution.

I would like to start by commending the authors for taking the time to respond to all the reviewer comments carefully. The comments from four independent reviewers were quite consistent.

After careful review, my overall impression of the manuscript remains unchanged.

- This is an important demonstration experiment of the potential of ultrafast XSS on organic chromophores in solution. However, the lack of signal-to-noise, prevents the discovery of new molecular-level information.
- This reviewer continues to remain concerned about the high excitation fluence. I appreciate the non-linear fit of the laser fluence scan in Fig. S8, which clearly shows that the data was measured in the non-linear regime excitation regime. This makes it challenging to understand the ensuing photochemical pathways taken. The authors have discussed this in the context of there being higher electronic states which are accessed and that they decay to the lowest excited electronic state on a short timescale. From an experimental standpoint, it is incorrect to interpret ultrafast photochemical experiments where the pump pulse is in the non-linear excitation regime. If the goal of this paper is simply to demonstrate the feasibility of ultrafast XSS on organic chromophores then, it has achieved its goal. If the goal is to identify specific physical phenomena (including solvation) following photoexcitation, then the higher fluence presents a problem.
- Several reviewers have pointed out that this work does not provide an "atomic-scale observation" of polar solvation dynamics. The authors have chosen to address this by adding following statement in the conclusion: "The experiments provide a clear atomic-scale signature of the transient solvation shell. However, neither the time evolution of specific solvent motions underlying the solvation-shell rearrangement nor any accompanying solute structural change could be resolved." The authors should consider changing the title of the paper and the abstract to clear indicate the limitations of this work.

While I have outlined my concerns about the manuscript above, I do believe that with advances at X-FELs we will be able to observe time-dependent, atomic-scale displacements in photoexcited solute molecules and the surrounding solvent and be able to provide an atomic description of organic photochemistry in solution. This paper is an early demonstration about the potential of this technique.

(Remarks on code availability)

Open Access This Peer Review File is licensed under a Creative Commons Attribution 4.0 International License, which permits use, sharing, adaptation, distribution and reproduction in any medium or format, as long as you give appropriate credit to the original author(s) and the source, provide a link to the Creative Commons license, and indicate if changes were made.
In cases where reviewers are anonymous, credit should be given to 'Anonymous Referee' and the source.
The images or other third party material in this Peer Review File are included in the article's Creative Commons license, unless indicated otherwise in a credit line to the material. If material is not included in the article's Creative Commons license and your intended use is not permitted by statutory regulation or exceeds the permitted use, you will need to obtain permission directly from the copyright holder.
To view a copy of this license, visit https://creativecommons.org/licenses/by/4.0/

Response to Reviewers of Manuscript NCOMMS-25-77889

Below, we provide a point-by-point response to the reviewers' comments. Changes made to the manuscript in response to the reviewers' comments are highlighted in blue in the annotated version of the revised manuscript provided with this resubmission. Each revision is marked with the label "RXCY", where X is the reviewer number and Y is the number of the reviewer's comment.

Reviewer 1

Summary
This manuscript reports a femtosecond time-resolved X-ray solution scattering (TR-XSS) study of hemithioindigo–julolidine (HTI-J) in acetonitrile, aiming to visualize charge-transfer-induced solvation dynamics at the atomic scale. By constructing a global fitting model that includes optical Kerr effect (OKE), vibrational relaxation (VR), solvation-shell rearrangement (SSR), and bulk heating (ΔT) components, the authors attempt to separate and interpret multiple solvent responses over sub- to tens-of-picosecond timescales. The work represents a meaningful attempt to extend TR-XSS to organic chromophores containing only light atoms and highlights the potential of model-based decomposition for disentangling complex solvent and solute responses.

Overall assessment
The work tackles an important and technically demanding problem in ultrafast structural chemistry: distinguishing charge-transfer-driven solvent reorganization from solute structural motion in a light-atom system. The experimental setup and analysis framework are sophisticated, and the topic is timely. However, several central claims and figures appear to overstate what the data can conclusively demonstrate, which can mislead the readers. The following points must be thoroughly addressed before the manuscript can be considered for publication.

R1C1 On the generalization and claim of "atomic-scale observation"
The title and abstract emphasize "atomic-scale observation of polar solvation dynamics," yet the analysis does not yield explicit atomic coordinates. The observed effects primarily are inferred from solvent density changes in the scattering profiles induced by charge transfer in the solute molecules, whose atomic-scale structural changes (twisting) are themselves not directly visible in their scattering signal. This phrasing may overstate what was actually observed with the technique. The authors should clarify that the study provides indirect structural sensitivity to solvent reorganization rather than a direct atomic reconstruction. A more accurate framing in the title or abstract, focusing on charge-transfer-driven solvent reorganization, would strengthen the manuscript's credibility.

Response R1C1
Indeed, the present TR-XSS analysis does not yield the time evolution along specific solute

or solute-solvent atomic coordinates because the solvation-shell rearrangement is modelled as $\beta(\Delta t)\Delta S_{\mathrm{SSR}}(q)$. Nevertheless, the transient changes in solvation-shell structure encoded in $\Delta S_{\mathrm{SSR}}(q)$ are resolved at the atomic length scale because the difference scattering signal is directly sensitive to changes in interatomic pair-distance distributions, as exemplified in Figs. 6 and 7 (in the manuscript, we use "atomic-scale" in the standard sense of atomic-length-scale structural sensitivity). To make it clear that the experiments capture the transient solvation-shell structure formed upon intramolecular charge transfer and its decay, rather than specific solvent motions or solute atomic coordinates, we have added the following sentence to the Conclusions section: "The experiments provide a clear atomic-scale signature of the transient solvation shell. However, neither the time evolution of specific solvent motions underlying the solvation-shell rearrangement nor any accompanying solute structural change could be resolved.". The text in several other parts in the manuscript has also been modified to make it clear that the current experiments are not able to resolve solute structural changes, as described in more detail in the replies to the next comments.

> **R1C2** On CT-only versus CT + twist models
> Figures 4-5 and Supplementary Figs. S19–S21 show that including a twist marginally improves the fit near 2–3 Å-1 but worsens agreement around 1 Å-1. However, the manuscript does not present quantitative criteria for assessing these differences. It would be helpful to include direct numerical comparisons (e.g., $\chi 2$ values) between the two models to support the argument. If the signal-to-noise ratio prevents discrimination, the authors should state explicitly that the current data cannot resolve solute geometry changes. Displaying both fits side by side in a single panel would make this limitation visually clear.

Response R1C2

Figure 7 has been modified to display side-by-side fits of an average of the data after subtraction of all contributions apart from $\Delta S_{\mathrm{SSR}}(q)$ with the two models (charge transfer and twist, and charge transfer only). the value of reduced χ^2 has also been included for each fit. The reduced χ^2 is found to be 4 in both cases. This indicates that the signal-to-noise ratio prevents discrimination between the charge transfer and twist and charge transfer only models. To make this clear, we have added the following sentence in the Solvation dynamics section: "The average experimental signal closely matches the simulated signal, with both models giving a similar quality of the fit (reduced $\chi^2 = 4$). For charge transfer and twisting, the fit is marginally improved between 2 to 3 Å^{-1}, but the signal-to-noise ratio does not allow for quantitative discrimination between the two models, meaning that the current data can resolve only the transient solvation-shell structure, not the transient solute structural changes.". Moreover, the new caption of Fig. 7 includes the following sentence: "The quality of the fit is similar in both cases, indicating that the current signal-to-noise ratio prevents a quantitative determination of the solute structural change.".

> **R1C3** On the definition and interpretation of the "SSR" component
> The term "solute and solvation-shell structural rearrangement (SSR)" suggests resolvable solute motion, yet the fits use time-invariant shape functions and provide no quantitative evidence of twisting. Because the observed features can be explained by charge redistribution alone, the terminology should be revised to "solvation-shell rearrangement" to avoid implying direct solute structural change. Related schematics (e.g., Fig. 5c, 5f) should also be modified so that solute twisting is presented as hypothetical rather than confirmed via TR-XSS. The text should clearly state that the experimental data primarily support solvent reorganization following charge transfer.

Response R1C3

We agree with the reviewer that the terminology "solute and solvation-shell structural rearrangement (SSR)" could be read as implying that the analysis of the TR-XSS data provides

clear evidence of a solute structural change. Therefore, we have revised the terminology of this component to "solvation-shell rearrangement (SSR)". Related figures and discussions were revised accordingly. The twisting has been removed from Fig. 5 (see also answer to the next comment), while the text of the manuscript has been modified in several parts to emphasize that the observation of solvent-shell reorganization at atomic length scale following charge transfer is the primary conclusion supported by the TR-XSS data (see also answers to the previous comments).

> **R1C4** On figure presentation and potential misinterpretation
> Some figures (notably Fig. 2b and Fig. 5f) depict a twisted geometry that may be interpreted as an experimentally observed structure. These should be redrawn to emphasize charge-transfer-driven polarization and solvent motion instead of definite twisting of the solute molecule. Figure captions should explicitly describe such illustrations as conceptual representations based on DFT calculations and transient absorption measurement, not experimental determinations by TR-XSS.

Response R1C4
We agree that the depiction of a twisted geometry in Figs. 2 and 5 poses a risk of misinterpretation. We have updated the caption of Fig. 2 to state explicitly that the twisting of HTI-J is supported solely by the DFT calculations, and is not experimentally determined by TR-XSS. As also explained in the answer to the previous comment, we revised Fig. 5 by removing the depiction of twisting to emphasize the observation of the solvation-shell rearrangement from the TR-XSS measurements.

> **R1C5** On the DFT assignment and relevance of the proposed twisting geometry
> The proposed twisting is supported by DFT calculations on a julolidine-substituted HTI derivative. However, static DFT geometries may not accurately reflect solvated structures under experimental conditions. The authors should justify how well the computed geometries correspond to the actual solute configuration in acetonitrile and clarify whether solvent effects were included in the calculations. It is recommended that the authors explore how the twisting geometry and the extent of charge transfer depend on a broader range of quantum chemical methods.

Response R1C5
Solvent effects are included in the DFT calculations through the implicit conductor-like polarizable continuum model (CPCM) for acetonitrile, as described in the Methods section.

To validate the DFT results, we have performed additional coupled cluster calculations at DLPNO-CCSD(T) level. These calculations are performed for the triplet state (T1). Since T1 is the lowest triplet state, a ground state method can be used, which makes it possible to include solvation effects in coupled cluster calculations. T1 and the singlet excited state (S1) differ only in the exchange interaction, so they are expected to exhibit similar electronic character and potential energy surface (see, e.g., *J Mol Model* 18, 2479 (2012) DOI:10.1007/s00894-011-1270-6). The CCSD(T) calculations are mentioned in the Methods section and the results presented in Supplementary Note 9.3. The results confirm the predictions of DFT for the S1 state, as they show that the excited state has a 90 degree twisted geometry and has a large dipole moment in acetonitrile (magnitude of the dipole moment of 33.9 D for CCSD(T) vs. 31.6 D for DFT/CAM-B3LYP).

> **R1C6** On excitation fluence and possible nonlinear effects
> The excitation conditions correspond to a pulse energy of 12 µJ at 470 nm focused to a 73 × 95 µm (FWHM) spot, which gives an average fluence of approximately 1.1 mJ mm-2 within the FWHM area, assuming that half of the pulse energy lies inside

the Gaussian FWHM circle. This fluence is relatively high for pump–probe experiments in solution and may enter the regime where multiphoton ionization or solvent plasma formation becomes relevant. Under such conditions, the number of solvated electrons and ionized solvent molecules can approach significant concentrations, potentially modifying the solvent response and introducing additional anisotropy or background signals in the transient scattering data. It would therefore be important for the authors to demonstrate that the TR-XSS signals scale linearly with excitation fluence and that the measurements were performed under single-photon excitation conditions. Power-titration tests or transient absorption data verifying linear excited-state population would provide valuable support.

Response R1C6

To investigate the signal response to laser pump fluence, laser fluence scans were carried out during the TR-XSS experiment and the results were included in Supplementary Note 4.1 and Supplementary Fig. S8 in the original submission. A linear as well as second order polynomial fit suggest that the dependency of the signal response on the laser fluence starts deviating from linearity between 10–13 μJ/pulse. An excitation energy of 12 μJ/pulse as used to collect the data is in the upper end of the linear regime and we observe a higher energy deposition into the solvent than expected for one-photon excitation (discussed in Supplementary Note 8). However, as detailed below, we exclude photoionization of the solvent or the solute, and attribute the higher-than-expected increase of temperature to multiphoton excitation to higher-energy states.

First of all, we exclude direct excitation and photoionization of the acetonitrile solvent. Ki et al. (*J. Am. Chem. Soc.* 143, 14261 (2021) DOI:10.1021/jacs.1c06088) report no evidence of such effects in acetonitrile when using a 400 nm laser with a fluence ∼50% higher than used in the present study. This is not unexpected, as the ∼12 eV (CRC Handbook of Chemistry and Physics 95 edn (2014)) ionization threshold for acetonitrile would entail a 4-photon process at 400 nm (3.1 eV). Secondly, we also exclude direct photoionization of the HTI-J solute molecules. Related stilbene and indigo compounds exhibit photoionization thresholds of ∼7.5 eV (CRC Handbook of Chemistry and Physics 95 edn (2014)) and ∼6.9 eV (*Chem. Phys. Lett.* 449, 11–17 (2007) DOI:10.1016/j.cplett.2007.10.015), respectively. This suggests that at 470 nm (2.6 eV) photoionization would require a 3-photon process. Since the laser fluence scans show only weak non-linear behavior at 12 μJ/pulse, we consider significant photoionization of the solute unlikely.

Given the near-linear fluence dependence at 12 μJ/pulse, we conclude that a fraction of the excited state population is excited into higher-lying excited states. These states are expected to rapidly relax non-radiatively to the lowest excited state, depositing the excess energy into the solvent. This causes the rapid increase ($\tau^1_{\Delta T} \sim 0.2$ ps) in the magnitude of the solvent temperature component seen in this study. Similar fluence-dependent solvent-heating contributions have been reported in TR-XSS studies performed under comparable experimental conditions (*Phys. Rev. Lett.* 122, 63001 (2019) DOI:10.1103/PhysRevLett.122.063001, *Chem. Sci.* 14, 2572 (2023), DOI:10.1039/d2sc06600a).

To reflect these important considerations better in the manuscript, we have added the following to the main text: "... and an excitation fluence of approximately 1.2 mJ/mm^{-2}, corresponding to the upper end of the linear-response region determined via a laser fluence scan (see Supplementary Note 4.1).", and "We conclude that this rapid solvent response arises from fast non-radiative relaxation following multiphoton excitation of a fraction of molecules to higher-lying states, as discussed in more detail in Supplementary Note 8. ... Except for the rapid onset of the heating signal ($\tau^1_{\Delta T} \sim 0.2$ ps) no contributions from the higher laser fluence in the TR-XSS experiments compared to the TAS could be discerned in our analysis.". We have additionally expanded Supplementary Note 8 to incorporate a more thorough discussion of this

potential concern.

> **R1C7** On extraction of the heating contribution
> The heating term is obtained by averaging the 55–60 ps trace under the assumption that non-thermal contributions have fully decayed. While this is reasonable given the reported 2.6 ps electronic lifetime, the robustness of this assumption should be demonstrated. Testing alternative time windows (e.g., 45–50 ps versus 55–60 ps) and reporting how the resulting heating profile varies would provide useful validation. Any residual non-thermal contribution could also be estimated from the model or transient absorption data and summarized in the Supplementary Information.

Response R1C7
We have added a new figure, Fig. S17, to Supplementary Note 5.1 showing that the difference between the isotropic difference scattering data averaged between 45-50 ps and between 55-60 ps can be explained by contributions from background artifact components alone. Apart from these, no residual contribution was observed in the scattering data at long time delays.

> **R1C8** On the treatment of solvent effects in DFT calculations
> The DFT calculations appear to be based on static geometries optimized within an implicit solvent model (CPCM). Such treatment may not adequately capture the specific solute–solvent interactions that influence the charge distribution and dipole moment in the charge-transfer state. To ensure consistency between theory and experiment, the authors should perform additional excited-state calculations that include explicit solvation, for example using a QM/MM approach, to evaluate whether the computed charge distribution and dipole moments remain consistent with the experimental data. This additional analysis would help determine whether the experimentally observed solvent response arises solely from electronic polarization of the solvent environment or also involves genuine structural rearrangements within the first solvation shell that are not represented in the implicit-solvent model.

Response R1C8
We agree that a static optimization in an implicit (CPCM) solvent cannot by itself describe solvent structural rearrangement in the solvation shell. However, our interpretation of the structural solvent response does not rely on the implicit solvent model but on equilibrium MD simulations with explicit acetonitrile molecules for both ground and excited state solute models (see Figs. 6 and 7 and related text in the manuscript). The DFT/CPCM calculations are used primarily to obtain the ground and excited state solute geometry and partial charges for the parametrization of the solute in the MD simulations. To make this clear, we have added the following to the Computational methods section: "The DFT calculations used to obtain equilibrium geometries and partial charges for the solute in the MD simulations employ the range-separated hybrid functional CAM-B3LYP ...".

Regarding the adequacy of an implicit solvent model, previous studies (see, e.g., *J. Phys. Chem. C* 123, 2056 (2019) DOI:10.1021/acs.jpcc.8b10768, and *J. Phys. Chem. B* 122, 2975 (2018) DOI:10.1021/acs.jpcb.7b12560) have shown that the excitation energy and the solvent-stabilization (solvation) energy obtained with polarizable continuum models are consistent with the corresponding ensemble-averaged values from fully quantum-mechanical calculations that include explicit solvent molecules. In the Methods section, we have added "Previous studies [19, 23] have shown that the excitation and solvation energy obtained with polarizable continuum models are consistent with the corresponding ensemble-averaged values from fully quantum-mechanical calculations including explicit solvent molecules.", where [19, 23] are references to the above mentioned articles.

Regarding the representation of structural rearrangements within the solvation shell, MD simulations based on fixed-value partial charges from DFT calculations with implicit solvent have been shown to describe the orientational response of the solvent molecules to charge transfer of the solute (*J. Phys. Chem. C* 123, 2056 (2019) DOI:10.1021/acs.jpcc.8b10768, *J. Phys. Chem. B* 109, 3553 (2005) DOI:10.1021/jp0456032). Moreover, a flexible solute can account for some polarization effects as the solute geometry adjusts to stabilize the solute-solvent interactions, which can change the instantaneous and average dipole moment relative to that of the equilibrium geometry in an implicit solvent environment, as shown in *J. Phys. Chem. B* 109, 3553 (2005) DOI:10.1021/jp0456032. To make this clear, we have added the following in the Methods section, page 19: "MD simulations with fixed-value partial charges have been shown to describe the orientational response of a polar solute to charge transfer in the solute [19, 23]. Moreover, using a flexible solute with fixed partial charges has been shown to capture some polarization effects, since the solute can distort in response to solute–solvent interactions and thereby change its instantaneous and average dipole moment relative to the equilibrium geometry in an implicit solvent [23].".

> **R1C9** On the anisotropic signal and its physical interpretation
> The Supplementary Information states that the anisotropic signal contains the response to the optical Kerr effect (OKE), and that this fast OKE component is used to define time zero and determine the time resolution. However, the anisotropic signal can also contain valuable information about the orientational dynamics of the photoexcited solute and the correlated motion of nearby solvent molecules. A more complete analysis of the anisotropic component would provide independent insight into the solvation mechanism. In particular, the authors should clarify whether contributions from the photoexcited solute or its first solvation shell are distinguishable from the pure OKE response of the bulk solvent. Demonstrating this separation, even qualitatively, would substantially reinforce the interpretation of solvent reorganization dynamics.

Response R1C9
We thank the reviewer for this suggestion. We have added a new section in the Supplementary Information, Supplementary Note 4.3, with an analysis of the anisotropic difference data based on singular value decomposition (SVD). The SVD shows that the anisotropic signal is dominated by two components with a short and a long decay time constants. Similar anisotropic signals with timescales of ∼60 fs and 1.5 ps have been observed for an acetonitrile solution in time-resolved X-ray scattering by Ki et al. (*J. Am. Chem. Soc.* 143, 14261 (2021) DOI:10.1021/jacs.1c06088), where they were assigned to orientational rearrangement of the solvent associated with the optical Kerr effect (OKE). Related anisotropic OKE signals have also been reported by Montoya-Castillo et al. for chloroform (*Phys. Rev. Lett.*, 129, 056001 (2022) DOI:10.1103/PhysRevLett.129.056001). On this basis, we attribute the two leading SVD components of the anisotropic data to the bulk-solvent OKE.

The OKE contributions dominate the anisotropic signal. Apart from them, the SVD contains an additional weaker component. Fitting its temporal evolution gives a decay constant of 2.5 ± 0.8 ps. This value is consistent, within uncertainty, with (i) the solvation-shell structural rearrangement (SSR) time constant obtained from the global analysis of the isotropic difference scattering data (3 ± 1 ps) and (ii) the excited state lifetime from the TAS data (2.6 ± 0.5 ps). However, the overlap with the timescale of the slow component of the OKE signal and significant artifacts affecting the anisotropic data limit robust quantitative separation of solvation shell contributions from the bulk-solvent OKE compared to the isotropic signal, where the OKE and SSR components have significantly different decay rates.

> **R1 Minor comments**

- **Terminology:** The term solute–solute is used throughout the manuscript (e.g., Fig. 7) to denote intramolecular scattering within a single solute molecule. However, this usage is unconventional and potentially misleading, because solute–solute typically refers to interference terms arising from scattering between different solute molecules in highly concentrated solutions. The authors should rectify this usage by adopting a clearer label such as solute term or intra-solute scattering.
- **Experimental conditions:** Include a compact table summarizing concentration, fluence, jet thickness, and pump–probe geometry.
- **Captions and legends:** Ensure that all figures specify whether heating and OKE contributions are included or subtracted.

Response R1 Minor comments
- **Terminology:** We thank the reviewer for pointing out this potentially misleading use of the terminology "solute-solute scattering" to describe the scattering arising from changes of intramolecular atomic distances. We have decided to adopt the terminology "intra-solute scattering".
- **Experimental conditions:** We added a new table in the beginning of Supplementary Note 4, Table S2, summarizing key experimental parameters of the TR-XSS measurements.
- **Captions and legends:** In the revised manuscript, we ensure that all figures specify whether heating and OKE contributions are included or subtracted.

Conclusion and recommendation

This manuscript presents an important step toward extending TR-XSS to light-atom organic chromophores and toward dissecting solvent reorganization following charge transfer. The experimental results are strong, and the analytical framework is promising. However, the current data do not provide clear evidence of solute twisting, and several textual and visual elements do not clearly convey this point. Clarifying terminology, presenting CT-only versus CT + twist comparisons quantitatively, and harmonizing figure representations would substantially improve the clarity and rigor of the paper.

R1 Additional comment

In the discussion of charge-transfer–induced structural responses, the authors cite Biasin et al. (Nat. Chem. 2021) as the only representative study. To provide a more comprehensive view of the field, it would be beneficial to also cite Heo et al. (Nat. Commun., 2022, 13, 522). This work used anisotropic TR-XSS to resolve atomic charge redistribution accompanying the bond cleavage of the I3- system in solution, which offers a relevant and complementary context to the solvation dynamics and structural reorganization discussed in the current manuscript. I believe citing this work would benefit readers and the community as a whole.

Response R1 Additional comment
We thank the reviewer for pointing us to the work of Heo et al. (*Nat. Commun.* 13, 522 (2022) DOI:10.1038/s41467-022-28168-0). We agree that this work is highly relevant, and have added a citation in the Introduction.

Reviewer 2

Photochemical and photophysical processes in molecules can play out quite differently in solution compared to the gas-phase. The reasons for this include steric effects, with solvent molecules hindering geometric changes in the solute, and the ability of the solvent to quench excited vibrations, allowing energy to dissipate from

the solute into the solvent. This can have significant effect on the final quantum yields of reactions and makes fragmentation a much more important channel in the gas-phase since the excess energy in the ground electronic state is often sufficient to break chemical bonds. Another important example of solvent effects appears if the solvent is polar and the excited electronic state has charge transfer character, in which case the solvent can rearrange to stabilise the charge separation. This effect is predicted by theory and has been observed spectroscopically.

In the current manuscript by Mitterer et al, the authors provide convincing evidence for the rearrangement of a polar solvent in response to an excited charge-transfer state. They carry out timeresolved X-ray solution scattering experiments at the SwissFEL, supported by transient absorption measurements and simulations. By doing this, the team is able to infer the real-space rearrangements of atoms in the solute and the solvent following excitation by a 470 nm pulse. This is a significant achievement by itself, but the experiment also constitutes a notable demonstration of the improvement in solution-phase X-ray scattering experiments over time, as well as a textbook example of the current state-of-the-art in this domain (especially in terms of the analysis).

While time-resolved gas-phase X-ray scattering only appeared in 2015 (10.1103/PhysRevLett.114.255501), solution-phase measurements have a longer history with early experiments done with the help of a mechanical chopper at Michael Wulff's beamline ID09 at the ESRF already carried out in the early 2000s (10.1103/PhysRevLett.87.195508). The cited pioneering paper by Wulff et al investigates I2 dissociation in solution, and catches a glimpse of the caging effect of the solvent. However, what this early solution phase paper has in common with later papers, despite very significant later improvements in the data analysis and improvements in the time-resolution following the arrival of XFELs, is the reliance on targets with strongly scattering atoms to achieve sufficient contrast to the solvent. Later examples include an impressive range of studies in organometallics from the current authors (eg [Fe(bmip)2]2+ in 10.1038/s41467-020-14468-w and Pt2POP4 in 10.1103/PhysRevLett.122.063001, etc) and work in systems such as [Au(CN)2–]3 by Ihee et al (see eg 10.1038/s41586-020-2417-3 and 10.1038/nature14163). However, the current work stands out by performing a study in an organic chromophore. Although this is arguably a somewhat technical point, it is nevertheless an important one, as it demonstrates the overall quality of the measurements that can now be achieved. In my view this is an important aspect of the current manuscript.

Overall, the paper is very well written and presents impressive results. I particularly appreciate the systematic and thorough analysis of the data - it is rare that the supplementary information is enjoyable to read, but in this instance, I gained a lot from reading the SI. I find that the paper is suitable for publication in Nature Communications, but, as always, there are some points that I would like the authors to clarify and/or expand upon first.

Questions and comments:

R2C1 The kinetic model used to interpret the experimental scattering data benefits greatly from the complementary transient absorption spectroscopy (TAS) measurements. However, from what I can tell the TAS measurements use a 490 nm excitation pulse, while the X-ray scattering experiment uses a 470 nm pulse. Could the authors please explain what consequences this discrepancy may have, and to what degree it

may affect the obtained rate constants (or their assignment)?

Response R2C1

Both wavelengths lie within the same lowest-energy absorption band and therefore populate the same excited electronic state. The main difference is that 470 nm deposits slightly more excess vibrational energy in the solute. The difference in photon energy between 470 and 490 nm is about 0.1 eV, which is modest compared to the width of the absorption band. In solution, this small additional excess energy is expected to be at least in part dissipated to the solvent before the passage of the wave packet through the conical intersection with the ground state. As a result, the subsequent population decay and solvation structural dynamics that determine the fitted kinetic time constants are not expected to depend strongly on whether excitation is at 470 or 490 nm.

R2C2 When reading the manuscript, I was somewhat concerned that the X-ray signal may contain contributions from higher excited states or even ionisation. However, when looking in the SI, I saw that the authors performed a 'power titration' to establish the highest power commensurate with linear excitation. This is excellent, and I wonder if it may be worth including a (brief) reference in the main manuscript pointing readers to the SI on this topic?

Response R2C2

We thank the reviewer for the suggestion. We have included a reference in the Methods section of the main manuscript that points to Supplementary Note 4.1, reporting a laser fluence scan (formerly "power titration").

We would like to mention that in response to reviewer comments R1C6, R3C4, and R4C1, we have expanded the discussion about nonlinearity and multiphoton excitation and would like to refer the reviewer to our responses to R1C6 and R4C1.

R2C3 The theoretical description of charge transfer states by DFT is not entirely unproblematic, unless one uses range-separated hybrid functionals, as the authors have indeed done here. Nevertheless, I wonder if the authors have carried out any benchmarking beyond what is already presented in the SI, which contains a test of a sequence of basis sets with the CAM-B3LYP functional. I appreciate that the size of the molecular target is somewhat prohibitive for higher level methods, but it would be encouraging to see some reference data obtained with a non-DFT method.

Response R2C3

To validate the DFT results, we have performed higher-level coupled cluster calculations using the DLPNO-CCSD(T) method. In order to include implicit solvent effects, these calculations are performed for the lowest triplet state instead of the singlet excited state (S1) state (see also the reply to R1C5 of Reviewer 1). Under the typical assumption that T1 and S1 have similar geometry and electronic character, the CCSD(T) calculations support the DFT predictions, as both methods yield a 90 degree twisted structure and a strong charge transfer character for the excited state (magnitude of the dipole moment of 33.9 D for CCSD(T) vs. 31.6 D for DFT/CAM-B3LYP). The new CCSD(T) calculations are briefly described in the Methods section and the results presented in Supplementary Note 9.3.

R2C4 On page 14 in the SI, the authors state that approximately 30% of the images are rejected from the data. This is quite high. The process for excluding data is well described, but could the authors explain better why so much data had to be excluded to begin with? It would be good to make this as clear as possible given that this seems to be an example of rather aggressive curating of experimental data.

Response R2C4
We agree with the reviewer that a 30% outlier rejection can be considered fairly aggressive, and have added the following new text to the Supplementary Note 4.2 to explain the rationale for this in more detail: "The stochastic nature of the self-amplified spontaneous emission (SASE) process at XFELs induces substantial shot-to-shot fluctuations in beam intensity, photon energy, and pointing. Combined with sample delivery instabilities and variations in experimental chamber conditions, these effects may alter the spatial distribution of scattered X-rays on the detector. Although mitigation strategies exist [9, 10], XFEL data reduction typically begins by discarding frames most affected. In the present work, images lacking beamline diagnostics (e.g., intensity monitors or timing tool data) or deviating by more than 10% from the median total scattering intensity within a scan were rejected (Fig. S10a). To compensate for beam–jet overlap variations, we applied a correlation filter between summed scattering intensity and the I0 diode signal, removing shots in the lowest and highest deciles (Fig. S10b). The filtering resulted in a rejection of approximately 30% of the images. A more relaxed outlier filtering with 20% of the images rejected was also tested during data reduction, but significantly increased the magnitude of background artifact components in the calculated difference scattering signal. Background artifact components in the TR-XSS data are discussed in Supplementary Note 4.6.". Here, [9, 10] are references to *Faraday Discuss.* 177, 443 (2015) DOI:10.1039/c4fd00203b and *J. Synchrotron Rad.* 33, 1, (2026), DOI:10.1107/S1600577525009737.

> **R2C5** The authors make a strong point in the manuscript about 'atomistic resolution'. Although I fully appreciate that the experimental signal is reflective of the spatial arrangement of the atoms, I feel that with such a narrow q-range (essentially 1-3.5 Å-1) and an instrument response function of 120 fs, perhaps one should be more careful in the manuscript to not overstate this, or at least acknowledge the limitations of the current data more clearly. I would also be curious to know what limited the q-range in the current measurements. Looking elsewhere in the literature, the authors' own measurements on Pt2POP4 at LCLS achieved qmax = 4.5 Å-1 (10.1103/PhysRevLett.122.063001) and Ihee's measurements on [Au(CN)2–]3 at SACLA achieved qmax = 6 Å-1 (10.1038/s41586-020-2417-3). What were the specific limitations here, and can these be overcome in the future? Could the authors perhaps include theoretical signals for a wider range of q, say up to 10 Å-1, in the SI to help interested readers think about how much information may be present at those higher q-ranges?

Response R2C5
Regarding the atomic resolution, the present TR-XSS analysis does not yield the time evolution along specific solute or solute-solvent atomic coordinates because the solvation-shell rearrangement is modelled as $\beta(\Delta t)\Delta S_{\text{SSR}}(q)$ (see also our response to R1C1). Nevertheless, the transient changes in solvation-shell structure encoded in $\Delta S_{\text{SSR}}(q)$ are resolved at the atomic length scale, because the difference scattering signal is directly sensitive to changes in interatomic pair-distance distributions (see Figs. 6 and 7). To clarify this, we have added the following sentence to the Conclusions section: "The experiments provide a clear atomic-scale signature of the transient solvation shell. However, neither the time evolution of specific solvent motions underlying the solvation-shell rearrangement nor any accompanying solute structural change could be resolved.".

Scattering signals were recorded over a q-range of approximately 0.4–5 Å^{-1}. Supplementary Fig. S11 shows the isotropic and anisotropic difference scattering signals within this range. At high q values (above ∼3.8 Å^{-1}), background artifact contributions influenced the modeling of the data; therefore, a smaller q-range was selected for the final global fitting of the X-ray scattering data. A discussion of this is provided at the end of Supplementary Note 5.2. We point

out that this work focuses on the solvation-shell rearrangement in response to charge transfer in an organic molecule, for which the amplitude of this signal is largest at low q.

Supplementary Fig. S30 was updated to display the simulated difference scattering signals up to $q = 10$ Å^{-1}. While the solute–solvent term dominates the total simulated signal at lower q, the total simulated signal at larger q mainly reflects contributions from the structural response of the solute. However, the presence of background artifacts at higher q and the limited signal-to-noise ratio of the data did not allow for a determination of a solute structural change. Future studies aiming to resolve solute structural changes in photoexcited organic molecules in solution may benefit from a larger q-range.

> **R2C6** In a similar vein to question 5 above, but somewhat speculative, do the authors think that it might eventually become possible to measure the actual rearrangement of electrons that corresponds to the charge transfer in the excited state? There are examples in the gas-phase. Perhaps first to come to mind is the study of charge transfer in a molecule by Weber et al (10.1073/pnas.2021714118), which has some similarities to the current study in terms of the analysis and which succeeds to isolate the signature of the charge transfer in the scattering signal. Arguably other examples in the gas-phase include more recent papers such as 10.1103/53h3-vykl and 10.1038/s41467-020-15680-4 (although not involving charge transfer per se).

Response R2C6

In principle, X-ray scattering is sensitive to purely electronic rearrangements, as demonstrated for isolated molecules in the works cited by the reviewer. Extending this capability to solution-phase systems is more challenging because the signal associated with changes in the electron density is typically much smaller than the contributions from solute and solvent atomic reorganization. Nevertheless, we expect that direct observations of excited-state electronic redistribution in solution will become increasingly feasible as XFEL sources advance, particularly through improved time resolution, higher photon energy and repetition rates, and continued progress in analysis and simulation frameworks needed to separate electronic and nuclear components. To reflect this perspective, we have added the following sentence to the Conclusions section after our discussion of future experimental and theoretical developments: "Moreover, while in the present study the photoinduced charge transfer is inferred indirectly through the solvent response, we anticipate that the aforementioned advancements will make it possible to achieve direct X-ray scattering observations of pure electronic rearrangements in solution, as already demonstrated for isolated molecules [74-76].", where [74-76] are references to the articles mentioned by the reviewer.

> **R2C7** Speaking of the global analysis of the experimental data presented in Section 6 of the SI, could the authors comment further on the limitations of the 'staged' fitting algorithm that they have implemented, whereby some constraints are determined in the first step before the final global fit? What is the risk that this introduces bias in the fit? That being said, I found the overall analysis to be compelling. In particular, I like how Figures S18, S21 and S22 make it quite clear that both the twisting and the charge transfer in the solute molecule come into play to explain the observed signal.

Response R2C7

We tested the influence of the regularization applied to the q-profiles of the optical Kerr effect, solvation shell rearrangement, and vibrational relaxation components by repeating the global analysis without regularization. The results were unchanged within uncertainty. Moreover, we found that the final global fit is sufficiently stable to converge reliably without regularization. For

this reason, we have removed the regularization for these components from the analysis presented in the manuscript and the Supplementary Information. The q-profile of the bulk solvent heating remains fully constrained as the average at late time delays is expected to accurately represent this contribution. We have updated the Methods section and Supplementary Note 6.1 to reflect these changes to the analysis.

> **R2C8** From what I understand, following photoexcitation the system eventually returns to the electronic ground state where the solvent quenches the vibrational excitation. In terms of the final yields of the photoexcitation, do the authors know (or can estimate) the final quantum yield of the Z versus the E conformation of the molecule?

Response R2C8
The Z/E photoisomerization yield of HTI-J has been measured by Wiedbrauk et al. in various solvents (*J. Am. Chem. Soc.*, 138, 12219 (2016) DOI:10.1021/jacs.6b05981). For polar solvents, they report quantum yields of $< 1\%$. We have added this information in the Introduction.

> **R2C9** The authors assign the ΔS2 anisotropic component in the scattering to the optical Kerr effect and use this to confirm time zero. This all good and well, but is there no structural information that could be extracted from the anisotropic signal for the aligned molecules? It seems to me that it would be interesting to consider this, in addition to the ΔS0. In this context, can the authors confirm that the isotropic ΔS0 signal is obtained via Legendre decomposition, rather than overall averaging of the total signal?

Response R2C9
We thank Reviewer 2 for the suggestion of investigating the anisotropic signal, which is in line with comment 9 by Reviewer 1. As also explained in our response to R1C9, we have performed a singular value decomposition (SVD) of the anisotropic difference data, which is presented in a new section of the Supplementary Information, Supplementary Note 4.3. The SVD confirms that the anisotropic signal is dominated by the ultrafast optical Kerr effect (OKE) of the bulk solvent used to define time zero. In addition, it identifies a much weaker non-OKE component with a decay time consistent within uncertainty with the time constant of the solvation-shell structural rearrangement (SSR) extracted from the isotropic TR-XSS analysis and with the excited state lifetime from TAS. This suggests that the anisotropic data contain a solvation-related contribution. However, overlap with the timescale of slow OKE component and significant artifacts in the anisotropic data preclude a robust quantitative separation of solvation-shell dynamics from the bulk-solvent OKE, compared to the isotropic data.

We can confirm that the isotropic ΔS_0 signal is obtained via Legendre decomposition (not by simple averaging), and have specified this in the main article and Supplementary Note 4.2.

Reviewer 3

> The manuscript presents an in-depth study of a nonequilibrium response of a polar solvent acetonitrile which is caused by charge transfer in the small organic molecule hemithioindigo (HTI) chromophore following it's photoexcitation by short femtosecond laser pulse. The work consists of experiments in static and time-resolved pump-probe absorption (VIS) spectroscopy, time-resolved pump-probe X-ray solution scattering (Swiss XFEL) and extensive computational experiments, namely density functional theory (DFT) and molecular dynamics (MD) simulations to probe the structural and electronic changes in HTI and the subsequent response of acetonitrile polar

solvent. The work utilizes advanced and robust techniques for data processing, data analysis and statistical analysis of the data. The results presented in this manuscript are of interest to scientists working in the fields of chemistry, photonics, photobiology and solvation dynamics.

Significance.
Solution X-ray scattering is a contrast X-ray imaging technique in which measured X-ray scattering signal is proportional to difference of densities (contrast) of solute and solvent. Therefore, for small organic molecules, such as HTI chromophore, it is notoriously challenging to disentangle scattering from the solute molecule and its solvent, in this case acetonitrile. Time-Resolved X-ray Solution Scattering (TRXSS) has capability to probe simultaneously structural properties of a solute molecule and solvent molecules, and their interaction after the reaction initiation, in this case photoinitiation of the solute chromophore. The authors studied how photoexcitation of the HTI chromophore changes distribution of its intramolecular charges and its structural geometry, and how acetonitrile solvent responds to the changes. The charge transfer properties of HTI were amplified by substituting stilbene fragments by julolidine group (HTI-J) which caused increase of dipole moment of the molecule upon photoactivation from 5.3 D in ground state to 27.3 D in the excited state (DFT calculations) and therefore amplified electrostatic interaction of polar solvent with the photoexcited molecule of HTI-J. The authors observed experimentally, analyzed and determined the sequence of the processes which occur in the system upon HTI-J photoexcitation: polarization of the polar solvent by the electric field of the pump laser light (optical Kerr effect), changing energy of HTI-J from the ground to exited state and charge redistribution in the solute, vibrational energy transfer from solute to a solvent, solvent response and electrostatic interaction of the solute molecule and the polar solvent. While changing structure of HTI-J upon photoexcitation does not contribute significantly to scattering patterns observed in the experiment, vibrational energy exchange and electrostatic interaction between the exited state of HTI-J and polar solvent does. The significance of this work is in observing structural changes in solute and solvent, encoded in the scattering patterns, identifying the nature of chemical processes governing photoexcitation of HTI-J and its energy exchange with polar solvent and capturing time scales and sequences of those processes. This work contributes significantly to understanding mechanisms of solvation dynamics of polar solvents.

Novelty and originality.
The novelty of this work is in observing experimentally a change is the scattering pattern due to the dipolar rearrangement of the solvent molecules surrounding photoexcited HTI-J, capturing the formation of the transient solvation shell structure on a $\sim$0.5 ps timescale and its re-equilibration as the system returns to the ground state with a time constant of $\sim$2.6 ps, This is the first time when such structural dipolar rearrangement of the solvent molecules following HTI-J photoexcitation is experimentally captured in X-ray solution scattering patterns and those experimentally observed structural changes are modeled on the atomic scale.

Data and methodology. Validity of the results.
The manuscript presents state of the art research both experimentally, methodologically and computationally. It combines cutting edge technologies in TRXSS with static and time-resolved optical spectroscopy and extensive in-silico experiments. The combination of all those tools is required to identify, assign and model physical

processes which occur upon photoexcitation of a small organic chromophore in a polar solute including energy dissipation, relaxation and charge re-distribution processes. The disentanglement of those processes is accomplished by a robust stepwise global optimization procedure guided by methods of singular vector decomposition, DFT and MD simulations. Methods section in the manuscript, and Supplemental Material present in detail robustness of procedures of statistical analysis and errors handling, data acquisition, data processing, data analysis and simulations and ensure validity of the results.

Comments and suggestions.

R3C1 Data presentation should be improved. The time-resolved scattering patterns are dominated by the solute heating signal caused by vibrational cooling of solute molecules and heating of the solvent molecules. It is very important to see how data looks after global fitting and subtraction of the solvent heating signal. In this regard map representation does not give much information about actual data and noise levels contained in the data. In addition to the maps (Figure 4) heating signal subtracted data should be presented for selected time delays where a major contribution from specific processes is identified. The data should show experimental errors, and the quality of the fits, discussed in the manuscript.

Response R3C1

We have added a new figure (Fig. S23) in Supplementary Note 6.2 showing the background artifact- and heat-subtracted isotropic difference scattering signal at selected time delays together with the model from global fitting as well as the model contributions attributed to the optical Kerr effect (OKE), the solvation-shell rearrangement (SSR), and vibrational relaxation (VR). The selected time delays are chosen as representative times where these components contribute most strongly. Experimental uncertainties obtained as error bars from polynomial noise estimation are also included. A reference to this figure has been added to the main text: "Heat-subtracted isotropic difference scattering traces at selected delay times, chosen to highlight where specific processes dominate, are shown in Fig. S23 together with the corresponding individual model components.".

R3C2 It is not clear why authors use acetonitrile patten from a ref [15] in the data analysis. The experimental data were normalized according to established standards. 1 HTI-J molecule is surrounded by 6400 acetonitrile molecules (Supplemental Materials, Liquid unit cell scaling) and in the fitting procedure of scattering pattern from [15] authors dismiss very small contribution for HTI-J molecule anyway. Why authors need fitting of the acetonitrile data, collected at different experimental conditions previously, and then rescale already normalized data. Did authors try to process the data without this additional scaling? Did they perform static measurements of the acetonitrile and temperature jump measurements in this solvent along with HTI-J photoexcitation measurements?

Response R3C2

The reference acetonitrile signal from Kjær et al. (*Phys. Chem. Chem. Phys.* 15, 15003 (2013) DOI:10.1039/C3CP50751C) corresponds to a known temperature increase of 1 K in the bulk solvent. By scaling our bulk solvent heating contribution to this reference, we can express the time-dependent temperature change, $\Delta T(\Delta t)$, in units of Kelvin. Omitting this calibration step does not change the quality of the fit or the extracted time constants. We have clarified this point in the text of Supplementary Note 5.1 and the caption of Fig. S18. We did not perform static or temperature jump measurements of acetonitrile during the HTI-J experiment at SwissFEL.

R3C3 The authors did laser power titration measurements for the laser wavelength 470 nm and proved that the measurements are performed in the linear regime. How many photons were delivered per HTI-J chromophore? Did authors try to estimate what additional fitting procedure (discussed above) could contribute to errors in the heating signal increase (temperature jump) and authors conclusions about multiphoton excitation in the experiment?

Response R3C3
We have included a second order polynomial fit to the laser fluence scan (Supplementary Note 4.1), which suggests that a laser excitation energy of 12 μJ/pulse employed in the experiments is at the upper end of the linear-response region. Using the pump laser energy of 12 μJ and a concentration of the solution of 3 mM, we estimate that approximately 5 photons were delivered per HTI-J chromophore. Given this and the near-linear fluence dependence at 12 μJ/pulse, we attribute the higher-than-expected increase in temperature to excitation of a fraction of the excited state population into high-energy states via multiphoton excitation. We would like to refer the reviewer to our response to R1C6 and R4C1 for further discussion on this.

When using the average between 55 and 60 ps of the isotropic difference scattering signal as the bulk solvent heating component, $\Delta S_{\Delta \mathrm{T}}$, without the scaling to e.u./LUC/K, the kinetic profile of this component exhibits a similar bi-exponential increase, but the amplitude, $\Delta T(\Delta t)$, does not reflect a temperature increase in Kelvin in this case.

R3C4 Multiphoton absorption in organic chromophores may significantly impact energy transfer and relaxation processes and trigger different pathways and rates. Therefore, it may alter the photophysical properties of the system. The authors should discuss in the main text what changes (if any) in the identified in the manuscript relaxation processes this may cause besides mentioned in the manuscript fast non-radiative relaxation from higher excited state causing fast solvent heating (0.2 ps, Table 1).

Response R3C4
The weak non-linear behavior at the employed laser fluence shown by the laser fluence scans suggests that multiphoton excitation only leads to excitation of a fraction of the excited state population into higher-lying excited states. Consistent with this, the only observable consequence in our TR-XSS data is the additional ultrafast heating component assigned to rapid non-radiative relaxation from higher-lying states. As also discussed in detail in our response to R1C6 and R4C1, we find no evidence for additional pathways that would alter the relaxation dynamics. In particular, photoionization of either solvent or solute is unlikely under our conditions, as it would require 4- or 3-photon excitation based on known ionization thresholds and because previous studies with similar experimental conditions, report no evidence of such effects. We have expanded Supplementary Note 8 to include a more detailed discussion of multiphoton effects and added a short discussion to the main text.

R3C5 DFT calculations and MD simulations are very important for the atomic-scale interpretation of the data. How MD simulations are used for the determination of the final structures, however, requires additional explanations. The manuscript presents exciting results of interplay between molecule of solute and surrounding molecules of solvent (solvation dynamics), including solvent excluded volume changes, changes in its interatomic distances and changes in polarization of the solvent. In Methods it is emphasized that for equilibrium MD simulations, 400000 equidistant frames were collected over 400 ns. It is not clear how these 400000 trajectories were used in the final selection of the structures. How many runs were performed, what were criteria

for the final structure selections? This should be explained, and appropriate data should be presented.

Response R3C5
We performed four sets of equilibrium MD simulations in the NVT ensemble for HTI-J in acetonitrile. One in which HTI-J is modelled in its ground state and three where HTI-J is described with different excited state models: charge transfer and twist, only charge transfer, and only twist. In each case, the equilibrium geometry and partial charges of the solute are obtained from DFT calculations in the ground or excited state. For each system, we performed an NVT production run of 400 ns, from which one frame every ps is sampled. The resulting 400 000 frames represented an equilibrum ensemble from which the radial distribution functions (RDFs) are calculated. No additional structure selection was performed beyond equilibration and uniform sampling.

We have extensively rewritten the part of the Computational methods section presenting the equilibrium MD simulations to make this workflow more clear, clarifying the number of simulations and sampling procedure.

R3C6 It is confusing to read sections of the main text of the manuscript describing solvent heating, vibrational relaxation, and optical Kerr effect and solvation dynamics with the detailed explanations of physics, energy flow and timing of multiple processes. It would be much easier for a reader if this information is also presented in a visual schematic or a diagram which a reader can follow in addition to the text description.

Response R3C6
To make the sequence and relative timing of the different processes easier to follow, we have added a plot of the kinetic profiles of the individual signal components to Fig. 5. This provides a visual representation of their temporal evolution, and we refer to this figure explicitly in the relevant part of the main text.

R3C7 Sentence lines 95–98 is not clear, it reads like "Knowledge . . . could reduce energy loss through thermal dissipation", perhaps it should be rephrased.

Response R3C7
We have rewritten this sentence, which now reads: "Such knowledge could ultimately enable control over the lifetime of charge-separated states and help reduce energy loss due to thermal dissipation, thereby improving efficiency and stability in photosensitization and photoconversion.".

References:
The manuscript references previous literature adequately.

Clarity:
The manuscript is written in clear language, understandable to an expert and a general audience. Argumentation is clear with the appropriate references when required. Abstract, Introduction and Conclusions are appropriate. Methods and Supplemental materials are very helpful.

Reviewer expertise:
My expertise is in time-resolved X-ray solution scattering and supporting experiments (for example optical spectroscopy) and analysis of time-resolved and static X-ray solution scattering data, in particular for biomolecules in solutions. The topic

of this manuscript does align partially with the field of my expertise.

My assessment that the manuscript can be published in Nature Communications with revisions, which address above comments

Reviewer 3 (Remarks on code availability):
I reviewed the code but did not run it. The code provides step-by step guide for data analysis of TRXSS and TRA. Fits clearly show good quality. The code is usable resource for the community and nice training tool for those who start their career in the field. README file provides lists of tutorials on how to run the code. It would be helpful if authors also provided Conda environment (instructions) as running the code requires multiple python-based packages installed.

Response Reviewer 3 (Remarks on code availability)
We have added an 'environment.yml'-file for conda to handle the required dependencies, and updated the README.md of the code to provide conda-specific installation instructions to complement the pip/venv-based instructions we had initially supplied.

Reviewer 4

This is an important experimental advance – obtaining time-resolved solution scattering on an organic molecule. Time-resolved X-ray solution scattering on the femto-picosecond timescale is now a well-established technique at X-FELs. To date, these experiments have been performed on solute-solvent systems where the solute contains high Z atom(s), which have strong scattering cross-sections.

Before the work is published the authors should address the following comments:

R4C1 The issue of high optical pump fluence 12 μJ/pulse needs to be addressed. From Figure S8, this reviewer does not agree that the data was collected in the linear regime. The data could be easily fit by higher order terms.
(a) What was the optical pump fluence used in the optical transient absorption experiments? If different from the X-ray experiments, then it should be rationalized, or the TA experiments must be performed with the same fluence for a 1:1 comparison.
(b) The pump fluence dependent study for the transient X-ray was conducted at 20 ps. At that time delay, there is very little difference signal from the solute. It seems that the pump-fluence is measuring the solvent heating response. Is that correct?
(c) At some point in the main text or SI, the authors point to multi-photon effects in the solvent. How does that match with the difference signal being linear in pump-fluence.

Response R4C1
We have added a second-order polynomial fit to the laser fluence scan (Fig. S8, Supplementary Note 4.1), which suggests that the laser fluence dependency begins to deviate from the linear regime around 10–13 μJ/pulse. With an excitation energy of 12 μJ/pulse, we are at the upper end of the linear regime and we observe a higher energy deposition into the solvent than expected for one-photon excitation (discussed in Supplementary Note 8), indicating multiphoton excitation of the solute.

(a) The optical pump fluence used in the transient absorption spectroscopy (TAS) experiments was approximately 2.6 μJ/mm^2, which is lower than the $\sim$1.2 mJ/mm^2 fluence of the pump

laser in the time-resolved X-ray solution scattering (TR-XSS) experiment. A higher pump fluence of around 1 mJ/mm^2 is typically necessary due to the repetition rate of these experiments (approximately 100 Hz), whereas TAS experiments generally operate at repetition rates of a few kHz, allowing sufficient signal-to-noise ratios to be achieved at lower pump power. While the internal conversion time obtained from TAS was used as an initial guess for the global fitting of the TR-XSS signal for the solvation-shell rearrangement (SSR) component, the right singular vector of the second SVD component, after subtracting bulk solvent heating and vibrational relaxation from the difference scattering signal, shows a time evolution on a similar timescale as obtained in the final global fit. The corresponding left singular vector resembles the simulated signal for a solvation-shell rearrangement in response to charge transfer in the solute, as shown in Fig. S21. Given the similar timescales observed for the excited state lifetime in TAS at lower excitation fluence and the solvation-shell rearrangement in TR-XSS, we conclude that multiphoton excitation of the solute only leads to population of higher-energy states, followed by ultrafast internal conversion to the lowest excited state, which then decays on a timescale similar to the excited-state lifetime observed in TAS. Photoionization processes that would alter significantly the relaxation pathways are excluded as discussed extensively in our reply to R1C6. This is consistent with (1) the regime of weak non-linearity at the employed laser energy of 12 μJ/pulse, and (2) the observed fast component of the rise of the heating signal ($\tau^1_{\Delta T} \sim 0.2$ ps). To better reflect these important considerations, we have substantially revised Supplementary Note 8 and added a discussion in the main text (see also our response to R1C6).

(b) Yes, this is correct. Although desirable, analyzing and interpreting an energy-fluence scan for the solute response during data acquisition is presently unfeasible given the small magnitude of the solute-related signal.

(c) After revising the laser fluence scan discussed in Supplementary Note 4.1 and adding the second-order polynomial fit, it becomes evident that the chosen laser fluence was at the upper end of the linear-response regime. In combination with the discussion in Supplementary Note 8, this supports the interpretation that multiphoton excitation of the solute occurred as a consequence of the applied laser energy.

> **R4C2** The authors have detailed their data analysis and data reduction. However, one is left wondering if the data is over-fit. The multiple timescales and nomenclature used in Table 1 of the main text are confusing. It is also odd that the transient absorption data does not measure the longer timescales for solute vibrational cooling or the slow solvent heating. Why are these not the same thing?

Response R4C2

The scattering data were globally fitted using 11 parameters associated with four physically justified processes, with q-profiles and contributions to the scattering signal identified through a stepwise analysis, as detailed in Supplementary Note 5. As described in Supplementary Notes 5.3 and 5.4, modeling the data with only the bulk solvent heating and vibrational relaxation components and subtracting these contributions from the signal leaves residual features that appear at time zero and evolve on a picosecond timescale. As shown in Fig. S20, singular value decomposition (SVD) of this residual signal reveals two components with clear temporal evolution. The component with largest amplitude is assigned to the optical Kerr effect, while the second component strongly resembles the simulated signal for solvation-shell rearrangement due to charge transfer in the solute, calculated from MD simulations (Fig. S21). The corresponding right singular vector evolves on timescales similar to those obtained in the global fit of the complete dataset. For these reasons, we do not believe that the data are overfitted.

To make Table 1 easier to read, we added spacing to group parameters related to each of the

four physically justified signal components. In the caption, we also clarified that the internal conversion time from optical transient absorption spectroscopy is included alongside the time constants obtained from the global fit of the TR-XSS data. Additionally, we added kinetic profiles to Fig. 5 to visualize the temporal evolution of the signal components in the main article, which will help readers to better follow the discussion in the text.

We agree that observing vibrational cooling in the ground state in the optical transient absorption data would provide additional support to our assignment of the vibrational relaxation component in the TR-XSS data. However, in our TAS measurements, the laser excitation energy of 490 nm lies close to the absorption maximum, and pump scatter overlaps the spectral region where the ground state bleach is expected. As a result, the ground state bleach cannot be observed, and we cannot extract a vibrational cooling time from the TAS data. Therefore, we relied on reference studies reporting vibrational cooling times in acetonitrile in the range of 10–15 ps, as discussed in the paragraph on vibrational relaxation in the main article. To make this limitation of the TAS data clear, we have added the following statement to the caption of Supplementary Fig. S5: "Due to pump scatter within the range of the absorption spectrum of HTI-J (see Fig. S4), the ground state bleach is not observed, and vibrational cooling in the ground state could not be inferred from the TAS data.".

> **R4C3** The authors claim that their work provides atomic scale resolution of polar solvation dynamics. However, they do not provide any non-equilibrium RDFs of the solute-solvent interactions via MD simulations. Their MD simulations should include non-equilibrium solute-solvent interactions because they fit these timescales in their data. If non-equilibrium MD simulations are not included, then there should be a discussion in the main text about what is missing.

Response R4C3
While the transient solvation-shell structure is resolved at the atomic length scale, the present TR-XSS analysis does not yield the time evolution along specific solute or solute-solvent atomic coordinates (see also our response to R1C1), which would require simulating nonequilibrium radial distribution functions. We have added the following sentence to the Computational methods section to make it clear that nonequilibrium MD simulations are not included: "... These simulations correspond to the HTI-J molecule in the excited state with a fully formed transient solvation shell, and do not account for nonequilibrium solvent motions during the solvation shell rearrangement.".

> In conclusion, while this is an important experimental demonstration, the data analysis and quality of the data do not provide any new physically relevant information.

Other changes not directly connected to the reviewers' comments

1. In the Introduction, we have added citations to the following articles, which we had missed in the original version of the manuscript:
   - Krell-Jørgensen et al. *Chem. Commun.* 59, 563 (2023) DOI:10.1039/d2cc05548a. A paper reporting on recently developed hemithioindigo derivatives closely related to the one investigated in the present manuscript.
   - Vester et al. *J. Chem. Phys.* 157, 224201 (2022). DOI:10.1063/5.0107224. A recent time-resolved X-ray spectroscopy and scattering study of the solvent reorganization following photoabstraction of an electron from a halogen ion.

2. In Fig. 7, we replaced the optimized q-profile of the ΔS_{SSR} component from global fitting with an average of the data for $0 < \Delta t < 5$ ps after subtracting contributions from background artifact components, heat, vibrational relaxation and the optical Kerr effect.

3. We added a specification of the loss function to the description of the global fitting algorithm in the Methods section in the main article and in Algorithm 1 in the Supplementary Information.

4. We renamed "power titration" to "laser fluence scan" and replaced "laser power" with "laser energy" when referring to the pump laser energy of the TR-XSS experiment.

5. We fixed typos and made other minor changes to the text.

Response to Reviewers of Manuscript NCOMMS-25-77889A

Below, we provide a point-by-point response to the reviewers' comments. Changes made to the manuscript and Supplementary Information (SI) in response to the reviewers' comments are highlighted in blue in the annotated version of the revised manuscript and SI provided with this resubmission.

Reviewer 1

> **Request for quantitative analysis of solvation shell (final action item)**
> While the authors have clarified in the text that the experiment tracks the solvation-shell rearrangement signal rather than specific atomic trajectories, the support for "atomic-scale observation" currently relies on qualitative comparisons of MD-derived Radial Distribution Functions (RDFs) in Figure 6. To bridge the gap between the qualitative visual evidence and the quantitative claim of "atomic-scale" sensitivity, a specific breakdown of the structural changes is necessary.
>
> **Suggestion:**
> I suggest including a table (in the Main Text or SI) summarizing the quantitative structural parameters extracted from the MD simulations for the first and second solvation shells. Specifically:
> - Peak positions (rmax) for relevant atom pairs (e.g., Solute-N, Solute-Methyl) in the ground state vs. charge-transfer state.
> - Coordination numbers (CN) for these shells.
> - The quantitative magnitude of the change (Δr, ΔCN) upon excitation.
>
> Providing these numbers will concretely define what "atomic-scale" changes are responsible for the observed scattering signal.

Authors' response

We thank the reviewer for this suggestion. We have added a new quantitative analysis of the calculated solute-solvent radial distribution functions (RDF) in Suppl. Note 10.1. In particular, we have included two new figures, one showing representative solute–solvent RDFs from the molecular dynamics simulations of HTI-J in the ground and excited states, and a second showing the ratio of the cumulative coordination numbers for the nitrogen and methyl carbon sites of acetonitrile, computed from the corresponding solute–nitrogen and solute–methyl RDFs. In addition, we have added a new table summarizing key structural parameters extracted from the RDF analysis: (i) the position of the first RDF maximum, $r_{g_{\max}}$, (ii) the mean distance within the first solvation shell, $\langle r \rangle$, and (iii) the coordination number of the first solvation shell, CN. These additions provide a quantitative measure of the structural changes in the first solvation shell upon excitation and clarify which atomic-scale rearrangements underlie the observed scattering signal. References to the quantitative RDF analysis presented in Suppl. Note 10.1 are included in the main text of the manuscript when introducing the results of the molecular dynamics simulations (Fig. 6) and after comparing the simulated X-ray scattering signal to the experimental data in Fig. 7.

Reviewer 4

> Several reviewers have pointed out that this work does not provide an "atomic-scale observation" of polar solvation dynamics. The authors have chosen to address this by adding following statement in the conclusion: "The experiments provide a clear atomic-scale signature of the transient solvation shell. However, neither the time evolution of specific solvent motions underlying the solvation-shell rearrangement nor any accompanying solute structural change could be resolved." The authors should consider changing the title of the paper and the abstract to clear indicate the limitations of this work.

Authors' response

The title and abstract of the manuscript have now been rewritten, addressing the concerns of the reviewer. The new title reads "Tracking Polar Solvation Dynamics of a Photoexcited Organic Chromophore with Ultrafast X-ray Scattering".

Review of K. M. Mitterer *et al* submitted to *Nature Communications*

Title: "*Atomic-Scale Observation of Polar Solvation Dynamics Triggered by Photoinduced Charge Transfer in an Organic Chromophore*"

Photochemical and photophysical processes in molecules can play out quite differently in solution compared to the gas-phase. The reasons for this include steric effects, with solvent molecules hindering geometric changes in the solute, and the ability of the solvent to quench excited vibrations, allowing energy to dissipate from the solute into the solvent. This can have significant effect on the final quantum yields of reactions and makes fragmentation a much more important channel in the gas-phase since the excess energy in the ground electronic state is often sufficient to break chemical bonds. Another important example of solvent effects appears if the solvent is polar and the excited electronic state has charge transfer character, in which case the solvent can rearrange to stabilise the charge separation. This effect is predicted by theory and has been observed spectroscopically.

In the current manuscript by Mitterer *et al*, the authors provide convincing evidence for the rearrangement of a polar solvent in response to an excited charge-transfer state. They carry out time-resolved X-ray solution scattering experiments at the SwissFEL, supported by transient absorption measurements and simulations. By doing this, the team is able to infer the real-space rearrangements of atoms in the solute and the solvent following excitation by a 470 nm pulse. This is a significant achievement by itself, but the experiment also constitutes a notable demonstration of the improvement in solution-phase X-ray scattering experiments over time, as well as a textbook example of the current state-of-the-art in this domain (especially in terms of the analysis).

While time-resolved gas-phase X-ray scattering only appeared in 2015 (10.1103/PhysRevLett.114.255501), solution-phase measurements have a longer history with early experiments done with the help of a mechanical chopper at Michael Wulff's beamline ID09 at the ESRF already carried out in the early 2000s (10.1103/PhysRevLett.87.195508). The cited pioneering paper by Wulff *et al* investigates I_2 dissociation in solution, and catches a glimpse of the caging effect of the solvent. However, what this early solution phase paper has in common with later papers, despite very significant later improvements in the data analysis and improvements in the time-resolution following the arrival of XFELs, is the reliance on targets with strongly scattering atoms to achieve sufficient contrast to the solvent. Later examples include an impressive range of studies in organometallics from the current authors (*eg* $[Fe(bmip)_2]^{2+}$ in 10.1038/s41467-020-14468-w and Pt_2POP_4 in 10.1103/PhysRevLett.122.063001, *etc*) and work in systems such as $[Au(CN)_{2-}]^3$ by Ihee *et al* (see *eg* 10.1038/s41586-020-2417-3 and 10.1038/nature14163). However, the current work stands out by performing a study in an *organic* chromophore. Although this is arguably a somewhat technical point, it is nevertheless an important one, as it demonstrates the overall quality of the measurements that can now be achieved. In my view this is an important aspect of the current manuscript.

Overall, the paper is very well written and presents impressive results. I particularly appreciate the systematic and thorough analysis of the data - it is rare that the supplementary information is enjoyable to read, but in this instance, I gained a lot from reading the SI. I find that the paper is suitable for publication in Nature Communications, but, as always, there are some points that I would like the authors to clarify and/or expand upon first.

Questions and comments:

1. The kinetic model used to interpret the experimental scattering data benefits greatly from the complementary transient absorption spectroscopy (TAS) measurements. However, from what I can tell the TAS measurements use a 490 nm excitation pulse, while the X-ray scattering experiment uses

a 470 nm pulse. Could the authors please explain what consequences this discrepancy may have, and to what degree it may affect the obtained rate constants (or their assignment)?

2. When reading the manuscript, I was somewhat concerned that the X-ray signal may contain contributions from higher excited states or even ionisation. However, when looking in the SI, I saw that the authors performed a 'power titration' to establish the highest power commensurate with linear excitation. This is excellent, and I wonder if it may be worth including a (brief) reference in the main manuscript pointing readers to the SI on this topic?

3. The theoretical description of charge transfer states by DFT is not entirely unproblematic, unless one uses range-separated hybrid functionals, as the authors have indeed done here. Nevertheless, I wonder if the authors have carried out any benchmarking beyond what is already presented in the SI, which contains a test of a sequence of basis sets with the CAM-B3LYP functional. I appreciate that the size of the molecular target is somewhat prohibitive for higher level methods, but it would be encouraging to see some reference data obtained with a non-DFT method.

4. On page 14 in the SI, the authors state that approximately 30% of the images are rejected from the data. This is quite high. The process for excluding data is well described, but could the authors explain better why so much data had to be excluded to begin with? It would be good to make this as clear as possible given that this seems to be an example of rather aggressive curating of experimental data.

5. The authors make a strong point in the manuscript about 'atomistic resolution'. Although I fully appreciate that the experimental signal is reflective of the spatial arrangement of the atoms, I feel that with such a narrow q-range (essentially 1-3.5 $Å^{-1}$) and an instrument response function of 120 fs, perhaps one should be more careful in the manuscript to not overstate this, or at least acknowledge the limitations of the current data more clearly. I would also be curious to know what limited the q-range in the current measurements. Looking elsewhere in the literature, the authors' own measurements on Pt_2POP_4 at LCLS achieved q_{max} = 4.5 $Å^{-1}$ (10.1103/PhysRevLett.122.063001) and Ihee's measurements on [Au(CN)2–]3 at SACLA achieved q_{max} = 6 $Å^{-1}$ (10.1038/s41586-020-2417-3). What were the specific limitations here, and can these be overcome in the future? Could the authors perhaps include theoretical signals for a wider range of q, say up to 10 $Å^{-1}$, in the SI to help interested readers think about how much information may be present at those higher q-ranges?

6. In a similar vein to question 5 above, but somewhat speculative, do the authors think that it might eventually become possible to measure the actual rearrangement of electrons that corresponds to the charge transfer in the excited state? There are examples in the gas-phase. Perhaps first to come to mind is the study of charge transfer in a molecule by Weber *et al* (10.1073/pnas.2021714118), which has some similarities to the current study in terms of the analysis and which succeeds to isolate the signature of the charge transfer in the scattering signal. Arguably other examples in the gas-phase include more recent papers such as 10.1103/53h3-vykl and 10.1038/s41467-020-15680-4 (although not involving charge transfer *per se*).

7. Speaking of the global analysis of the experimental data presented in Section 6 of the SI, could the authors comment further on the limitations of the 'staged' fitting algorithm that they have implemented, whereby some constraints are determined in the first step before the final global fit? What is the risk that this introduces bias in the fit? That being said, I found the overall analysis to be compelling. In particular, I like how Figures S18, S21 and S22 make it quite clear that both the twisting and the charge transfer in the solute molecule come into play to explain the observed signal.

8. From what I understand, following photoexcitation the system eventually returns to the electronic ground state where the solvent quenches the vibrational excitation. In terms of the final yields of the

photoexcitation, do the authors know (or can estimate) the final quantum yield of the Z *versus* the E conformation of the molecule?

9. The authors assign the ΔS_2 anisotropic component in the scattering to the optical Kerr effect and use this to confirm time zero. This all good and well, but is there no structural information that could be extracted from the anisotropic signal for the aligned molecules? It seems to me that it would be interesting to consider this, in addition to the ΔS_0. In this context, can the authors confirm that the isotropic ΔS_0 signal is obtained *via* Legendre decomposition, rather than overall averaging of the total signal?
